# Serum albumin and cardiovascular disease: a Mendelian randomization study

**DOI:** 10.1186/s12872-024-03873-4

**Published:** 2024-04-05

**Authors:** Taoke Huang, Zhifeng An, Ziru Huang, Weiyang Gao, Benchuan Hao, Juan Xu

**Affiliations:** 1 Medical School of Chinese PLA, Beijing, 100853 China; 2 Department of Emergency Medicine, The 969th Hospital of the Joint Logistics Support Force of PLA, Hohhot, 010051 China; 3 School of Medicine, Nantong University, Nantong, 226000 China; 4 Department of Cardiology, The Second Medical Centre, Chinese PLA General Hospital, Beijing, 100853 China; 5 Department of General Surgery, Affiliated Xiaoshan Hospital, Hangzhou Normal University, Hangzhou, 311202 China

**Keywords:** Cardiovascular disease, Serum albumin, Mendelian Randomization

## Abstract

**Background:**

An increasing body of evidence suggests that serum albumin levels play a role in cardiovascular diseases. However, the specific causal relationship between serum albumin levels and cardiovascular disease remains partially unknown.

**Methods:**

Mendelian randomization (MR) was employed in this study to examine potential causal relationships between instrumental variables and cardiovascular diseases. Specifically, we utilized genetic variants of serum albumin levels within the reference range as our instrumental variables. To acquire data on genetic associations with cardiovascular diseases, we sourced information from renowned genome-wide association studies such as UK BioBank, EMBL-EBI, and FinnGen. Notably, our study leveraged summary statistics from large cohorts that have been previously described.

**Results:**

We explored the association between serum albumin levels and various conditions, including heart failure (HF), venous thromboembolism (VTE), stroke, atrial fibrillation (AF), coronary artery disease (CAD), type 2 diabetes (T2DM), and pulmonary heart disease (PHD). Genetically predicted serum albumin levels were associated with PHD (odds ratio = 0.737, 95% CI = 0.622 − 0.874, *P* < 0.001), AF (odds ratio = 0.922, 95% CI = 0.870 − 0.977, *P* = 0.006), VTE (odds ratio = 0.993, 95% CI = 0.991 − 0.995, *P* < 0.001), and Stroke (odds ratio = 0.997, 95% CI = 0.995 − 0.999, *P* = 0.002). However, genetically predicted serum albumin level traits were not associated with HF, CAD and T2DM.

**Conclusion:**

Our study demonstrates a significant association between serum albumin levels and cardiovascular disease, underscoring the crucial role of low serum albumin as a predictive factor in patients with cardiovascular disease.

**Supplementary Information:**

The online version contains supplementary material available at 10.1186/s12872-024-03873-4.

## Introduction

Cardiovascular diseases (CVDs) have been a significant cause of mortality globally, resulting in 17.8 million deaths worldwide in 2017. The etiology of CVD cannot be explained by any single cause and results from a combination of multiple outcomes [1]. However, low serum albumin remains an overlooked predictor in patients with CVD [2].

Observational findings have highlighted the involvement of serum albumin level in the occurrence and development of CVDs [3]. A recent prospective study published in 2020, which monitored over 100,000 individuals for 8.5 years, presented consistent and confirmatory findings. It revealed that a low serum albumin level independently predicted the occurrence of ischemic heart disease, myocardial infarction, and ischemic stroke [4]. Additionally, the Japanese JASPER registry, released in 2018, tracked 535 hospitalized patients with acute diastolic heart failure for 731 days and confirmed that hypoalbuminemia upon admission was a robust and independent predictor of death and admission due to worsening heart failure [5]. Albumin is the main protein in the blood, accounting for 50% of the plasma protein content, and plays a vital role in binding and transporting various drugs and substances in the plasma. Furthermore, it serves a vital function in regulating the oncotic (colloid-osmotic) pressure of blood, thereby influencing the physiological processes of the circulatory system [3, 6–8]. Low serum albumin levels can be caused by liver impairment resulting from acute phase inflammatory processes, increased excretion through the kidney, malnutrition, increased catabolism, enteral loss, severe volume overload, or escape into the interstitial space [9]. Decreased levels of serum albumin, known as hypoalbuminemia, have been observed in patients with severe forms of myocardial infarction (MI) or injury, heart failure (HF), stroke, hip fracture, malignancy, and renal disease [8, 10, 11]. Hypoalbuminemia has been associated with increased morbidity and mortality in hospitalized patients, regardless of the presence of comorbidities [11]. Further investigation is warranted to explore the association between serum albumin levels and CVDs. However, it is crucial to acknowledge that the existing evidence from observational studies may be influenced by confounding or reverse causation bias. Moreover, the causal relationship between serum albumin levels and CVDs remains incompletely understood.

Mendelian randomization (MR) employs genetic variants as proxies for the exposure of interest, simulating a randomized controlled trial (RCT) in which genetic alleles are randomly assigned during conception [12]. MR analysis relies on Mendel's second law and the random distribution of genetic variants at conception, making them unlikely to be associated with potential confounders. Therefore, MR can offer evidence of causality that is less susceptible to confounding or reverse causation when specific assumptions are met. These genetic variants exhibit a robust association with the exposure of interest but lack associations with confounders influencing the exposure-outcome relationship, and do not influence the outcome through pathways other than the exposure of interest [13]. Serum albumin, known for its affordability and convenience in clinical testing, holds promise in improving the prognosis of cardiovascular diseases by elucidating its potential causal relationship. This insight not only enhances the foundation for clinical decision-making but also offers a guiding framework for further cardiovascular disease research.

Highly powered, genome-wide association studies (GWAS) have successfully identified numerous single-nucleotide polymorphisms (SNPs) that are correlated with serum albumin levels and CVDs. This wealth of data enables the investigation of genetic links and potential causal relationships between serum albumin levels and CVDs using the MR approach.

Recent MR studies have examined the causal effects of serum albumin concentrations on atrial fibrillation (AF), venous thromboembolism (VTE) and heart failure (HF) [14–16]. Nevertheless, it remains partly unknown whether hypoalbuminemia is a causal connection of other CVDs, such as stroke, coronary artery disease (CAD), Type 2 diabetes (T2DM) and pulmonary heart disease (PHD). Therefore, we performed a two-sample MR analysis to examine whether serum albumin levels have causal associations with CVDs.

## Methods

### Study design

**Fig. 1:**
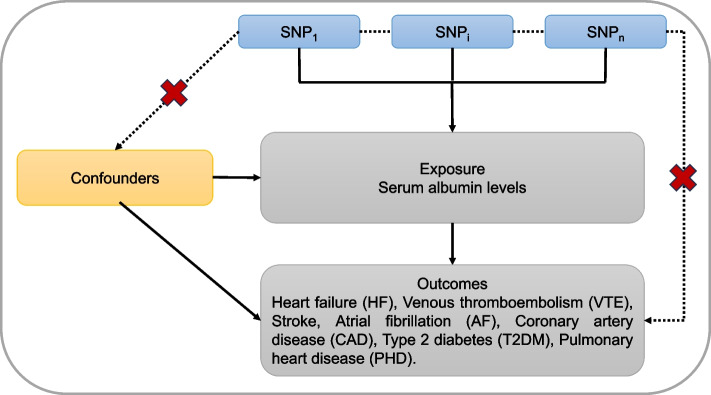
Principles of MR analysis for serum albumin level traits and risk of cardiovascular diseases outcomes and assumptions that need to be met to obtain unbiased estimates of causal effects. Broken lines represent potential pleiotropic or direct causal effects between variables that would violate MR assumptions. Three assumptions of MR: (1) genetic variants must be associated with serum albumin levels; (2) genetic variants must not be associated with confounders; (3) genetic variants must influence the risk of cardiovascular traits and disease outcomes only through serum albumin levels and not through any alternative pathways. MR: Mendelian randomization

The MR approach employed in this study relies on three key assumptions (Fig. 1). Firstly, the selected genetic variants serving as instrumental variables (IVs) should exhibit associations with serum albumin concentration. Secondly, these genetic variants should not be linked to any confounding factors. Lastly, the genetic variants should solely influence cardiovascular-related traits and events through serum albumin level, excluding alternative pathways [13]. The second and third assumptions are commonly referred to as the independence from pleiotropy. For our present MR analysis, we implemented a study design encompassing 7 disease outcomes, which include HF, VTE, stroke, AF, CAD, T2DM and PHD. All studies had been approved by a relevant ethics review board and participants provided informed consent.

### Selection and validation of IVs

We selected the lead SNPs (*P* < 5 × 10^−8^) for all genetic loci that have been shown to independently associate with the serum albumin level as IVs from the largest GWAS among individuals of European ancestry to date. Summary-level data of the serum albumin levels were obtained from the UK BioBank cohort with a sample size of 315,268 and a European population (https://www.ukbiobank.ac.uk/) [17]. The *p* values of the IVs are reported in Supplementary Table S1.

To ensure compliance with assumptions 1 and 2, our study performed several analyses. Firstly, we investigated the genetic association between the selected SNPs and each serum albumin level trait. Additionally, SNPs with R^2^ > 0.01 and within 10,000 kb distance were identified as in linkage disequilibrium and were excluded from the study. For serum albumin levels, 203 SNPs were extracted and used as IVs. The number of used SNPs and details of SNPs included are shown in Supplementary Table S1. By following this selection process, we maintained the integrity of assumption 2. It is worth noting that all SNPs associated with the same serum albumin level trait exhibited a significant association, as indicated by an F-statistic greater than 10. This high strength of the instrument confirms that assumption 1 was met.

### Cardiovascular diseases and data sources

For disease outcomes, summary-level data were extracted from the EMBL's European Bioinformatics Institute (https://www.ebi.ac.uk/) for AF (*n* = 1,030,836), T2DM (*n* = 655,666), CAD (*n* = 141,217), the FinnGen consortium (https://www.finngen.fi/en) for PHD (*n* = 95,023) and the UK BioBank cohort (https://www.ukbiobank.ac.uk/) for HF (*n* = 361,194), Stroke (*n* = 361,194), VTE (*n* = 361,194) [17–20]. Details on GWASs from which we extracted summary-level data are presented in Table 1. The summary of genetic associations datasets are reported in the Supplement (Table S2 − Table S8).

**Table 1:** Description of cardiovascular diseases

Outcomes	Consortium or study	Sample size	Population	Year	ID
HF	UK BioBank	361,194	Europeans	2018	ukb-d-HEARTFAIL
VTE	UK BioBank	361,194	Europeans	2018	ukb-d-I9_VTE
Stroke	UK BioBank	361,194	Europeans	2018	ukb-d-C_STROKE
AF	EMBL-EBI	1,030,836	Europeans	2018	ebi-a-GCST006414
CAD	EMBL-EBI	141,217	Europeans	2015	ebi-a-GCST003116
T2DM	EMBL-EBI	655,666	Europeans	2018	ebi-a-GCST006867
PHD	FinnGen	95,023	Europeans	2021	finn-b-I9_PULMHEART

### Statistical analysis

The main method used to assess the causal associations between serum albumin level and CVD was the inverse-variance weighted (IVW) method. This approach was supported by multiple sensitivity analyses, including MR-Egger and weighted median methods. MR-PRESSO (Mendelian Randomization Pleiotropy RESidual Sum and Outlier) method was used for detecting and correcting for the potential outliers [21]. The weighted median method has the advantage of providing consistent causal estimates, even when a substantial portion of the weight in the MR analysis is derived from invalid instrumental variables [22]. The MR-Egger regression method plots the effect of IV on the exposure against its effect on the outcome, and a non-zero intercept indicates the presence of pleiotropic effects. Additionally, the slope of the MR-Egger regression provides pleiotropy-corrected causal estimates [23]. To assess the stability of these genetic variants, we employed a leave-one-out analysis, systematically excluding one individual SNP at a time.

Two-sample MR analyses and MR-PRESSO method were conducted using “TwoSampleMR,” and “MR-PRESSO,” R packages for such analyses. It was performed using R version 4.2.3 (RStudio, PBC, USA). We used the threshold of statistical significance of *P* ≤ 0.007 (0.05/7) after Bonferroni correction. *P* ≤ 0.05 but above the Bonferroni corrected significance threshold was considered suggestive of evidence for a potential association.

## Results

**Fig. 2:**
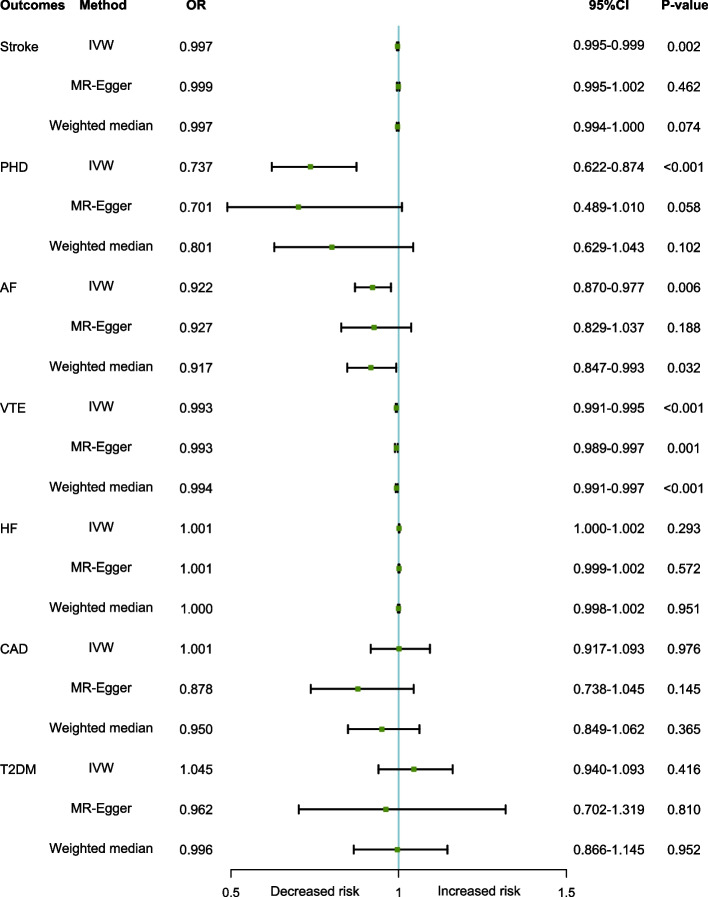
Mendelian randomization estimates of serum albumin concentration on the risk for CVD. OR, Odds ratio; CI, Confidence interval; IVW, inverse-variance weighted; PHD, pulmonary heart disease; AF, atrial fibrillation; VTE, Venous Thromboembolism; HF, heart failure; CAD, coronary artery disease; T2DM, type 2 diabetes

The association between genetically predicted serum albumin level and CVDs is displayed in Fig. 2 and Supplementary Table S8. MR-PRESSO detected some outliers in the analyses and we eliminated these outliers and used the IVW method. Per SD increase in serum albumin levels was suggestively associated with a 26.3% lower risk of PHD (OR = 0.737, 95% CI [0.622 − 0.874], *P* < 0.001), 0.78% lower risk of AF (OR = 0.922, 95% CI [0.870 − 0.977], *P* = 0.006), and can translate into lower risk of VTE (OR = 0.993, 95% CI [0.991 − 0.995], *P* < 0.001), and Stroke (OR = 0.997, 95% CI [0995 − 0.999], *P* = 0.002), but not with HF (OR = 1.001, 95% CI [1.000 − 1.002], *P* = 0.293), CAD (OR = 1.001, 95% CI [0.917 − 1.093], *P* = 0.976), and T2DM (OR = 1.045, 95% CI [0.940 − 1.161], *P* = 0.416). No horizontal pleiotropy was identified with MR-egger method. Scatter plots for the associations including the exposure with HF, VTE, stroke, AF, CAD, T2DM, and PHD are shown in Supplementary Figures S1–7. In leave-one-out analyses, shown in Supplementary Figures S8–14, we found that no single genetic variants had a significant impact on the results for CVDs.

## Discussion

In this study, we employed MR for the first time to extensively investigate the potential causal effects between serum albumin levels and CVD risk. Our findings demonstrate that individuals with a genetic predisposition towards low serum albumin levels face an elevated risk of stroke, PHD, AF, and VTE. Furthermore, no MR evidence suggests the presence of a potential causal relationship between genetic susceptibility to HF, CAD, and T2DM risk.

To the best of our knowledge, this study represents the first application of MR study to investigate the causal relationship between serum albumin levels and stroke, PHD, CAD, and T2DM. Previous research has established a significant association between serum albumin levels and CVDs [2]. The potential role of serum albumin levels in CVD primarily stems from its anti-inflammatory, antioxidant, and antithrombotic activities [3, 7, 24–26]. While the anti-inflammatory properties of serum albumin are not yet fully understood in the clinical context, its antioxidant effects in CVD have been well established [3]. Hypoalbuminemia can also play a part in other pathophysiological mechanisms, including the promotion of pulmonary edema and fluid retention. These outcomes are particularly detrimental to patients with established or high risk of HF and may additionally contribute to the deterioration of ischemic heart disease by promoting myocardial edema, thus exacerbating myocardial dysfunction [3].

Previous observational studies provided evidence about the associations of low serum albumin concentration and the risk factors of stroke [27, 28]. Our study, for the first time, by using MR method, demonstrated that the higher levels of serum albumin show a causal protective effect on the risk of stroke. Importantly, our results are less prone to biases stemming from confounding factors and reverse causation. This holds particular significance in the context of disease conditions, where considerable alterations occur in circulating metabolites within the human microenvironment. As such, our data provide further evidence supporting a causal association between serum albumin and the susceptibility to stroke. Similarly, we also analyzed associations between serum albumin level and PHD. Previous research has demonstrated that the serum albumin concentration serves as an independent prognostic indicator for patients with pulmonary hypertension [29]. Prior to our study, no research has explored the causal relationship between serum albumin levels and PHD. Our study is the first to demonstrate the protective effect of serum albumin in patients with PHD from a causal perspective. In light of our findings, it may be imperative to evaluate serum albumin level in patients with PHD.

In a recent retrospective case–control study, the relationship between serum albumin levels and AF was investigated. The study included 950 patients with AF and 963 age- and sex-matched non-AF patients who were in sinus rhythm [30]. The results showed that albumin levels were significantly lower in both men and women with AF (*P* < 0.05), particularly in cases of paroxysmal AF. Subsequent adjustment for confounding factors revealed an independent, negative association between albumin levels and AF in men (OR = 0.89, *P* < 0.05). However, a previous MR analysis did not support the causal role of serum albumin in the etiology of AF [14]. In our MR study, more SNPs were included in the analysis by using the latest large GWAS datasets, improving the reliability of the results and demonstrating that elevated serum albumin levels have a causal protective effect on the development of AF. Moreover, several observational cohort studies have shown that individuals with lower serum albumin levels are at an increased risk of VTE [31, 32]. A Mendelian randomization study has demonstrated a causal protective effect on the risk of VTE, indicating that higher levels of serum albumin and total protein are associated with lower risk [16]. Our study reaffirms the presence of these effects in a significantly larger population.

Although the potential association between serum albumin and the risk of HF remains uncertain, further investigation is warranted to elucidate its nature and magnitude. A study involving 5795 elderly individuals, tracked over a period of 9.6 years, confirms that the incidence of new onset heart failure was significantly associated with low serum albumin concentration, independent of risk factors, body mass index, and inflammation [33]. Nevertheless, neither our study nor previous MR studies found any evidence of a causal relationship between serum albumin levels and HF [15]. This indicates that the inverse associations observed in both the current and previous observational studies may be influenced by biases, such as unmeasured confounding or reverse causation. Previous clinical studies have established that low serum albumin is an independent risk factor for coronary artery disease. In the “Framingham Offspring” study involving 4,506 individuals followed for 22 years, low serum albumin was an independent predictor of first myocardial infarction after adjustment for the usual risk factors [34]. Similarly, in a separate study involving 7,647 individuals, low serum albumin displayed a robust and independent association with the occurrence of a first episode of myocardial infarction, irrespective of traditional risk factors [35]. Our results are inconsistent with most previous studies in terms of the association between serum albumin level and CAD risk. This may be because the onset of CAD is triggered by genetic and environmental factors, and we evaluated the association between serum albumin level and CAD from a genetic perspective. In addition, the MR study considered lifetime effects rather than short-term effects, which might explain the differences between our findings and previous literature. CVDs are the leading cause of death in patients with T2DM. A cohort study encompassing 1785 participants revealed a nearly linear, positive, and independent correlation between serum albumin levels and T2DM. However, it is noteworthy that this association did not yield improvements in event discrimination [36]. In our study, no evidence of a causal relationship between serum albumin levels and T2DM was found.

This study has several limitations that should be considered. Firstly, MR relies on stringent core assumptions. While we carefully selected SNPs at the genome-wide significance level, and F-statistics indicated a robust genetic association with serum albumin levels, it is important to acknowledge that our findings could potentially be influenced by weak instrument bias. Secondly, due to resource constraints, we were unable to perform population stratification analysis. Consequently, it is important to note that our conclusions might be compromised if there are significant differences in allele frequency between diverse populations. Thirdly, it is worth noting that the results of this study are based solely on individuals of European ancestry. To provide a more comprehensive understanding, future studies should investigate mixed populations or other ethnicities to expand upon our conclusions. Fourthly, the OR values for VTE and Stroke are relatively low in terms of reducing the percentage risk and should be carefully interpreted. By acknowledging and addressing these limitations, we aim to ensure a clear and accurate representation of our study's findings. Our study on MR is observational, and further research is necessary to validate the potential causal relationship we have uncovered.

## Conclusions

In summary, our MR study has showcased the causal influence of genetically proxied lower levels of serum albumin on the risk of developing stroke, PHD, AF, and VTE. These findings suggest that targeting these factors could serve as a promising strategy for the prevention of CVD.

### Supplementary Information

**Supplementary Material 1.**

## Data Availability

No datasets were generated or analysed during the current study.
