# Supplementary material for: Serum albumin and cardiovascular disease: a Mendelian randomization study
Source: BMC Cardiovasc Disord. 2024 Apr 5;24:196. doi: 10.1186/s12872-024-03873-4 (PMC10996126; doi:10.1186/s12872-024-03873-4)

**Supporting information for**

**Serum Albumin and cardiovascular disease: A Mendelian Randomization Study**

*Taoke Huang, Zhifeng An, Ziru Huang, Weiyang Gao, Benchuan Hao, Juan Xu*

**Contents**

1. **Supplementary Table S1.** Genetic instruments for serum albumin
2. **Supplementary Table S2.** SNP serum albumin and SNP HF associations (per Effect Allele) of the instruments of serum albumin after data harmonization and outlier removal
3. **Supplementary Table S3.** SNP serum albumin and SNP VTE associations (per Effect Allele) of the instruments of serum albumin after data harmonization and outlier removal
4. **Supplementary Table S4.** SNP serum albumin and SNP Stroke associations (per Effect Allele) of the instruments of serum albumin after data harmonization and outlier removal
5. **Supplementary Table S5.** SNP serum albumin and SNP AF associations (per Effect Allele) of the instruments of serum albumin after data harmonization and outlier removal
6. **Supplementary Table S6.** SNP serum albumin and SNP CAD associations (per Effect Allele) of the instruments of serum albumin after data harmonization and outlier removal
7. **Supplementary Table S7.** SNP serum albumin and SNP T2DM associations (per Effect Allele) of the instruments of serum albumin after data harmonization and outlier removal
8. **Supplementary Table S8.** SNP serum albumin and SNP PHD associations (per Effect Allele) of the instruments of serum albumin after data harmonization and outlier removal
9. **Supplementary Table S9.** MR results and pleiotropy test
10. **Supplementary Figure S1.** Scatter plot: Serum albumin and HF
11. **Supplementary Figure S2.** Scatter plot: Serum albumin and VTE
12. **Supplementary Figure S3.** Scatter plot: Serum albumin and Stroke
13. **Supplementary Figure S4.** Scatter plot: Serum albumin and AF
14. **Supplementary Figure S5.** Scatter plot: Serum albumin and CAD
15. **Supplementary Figure S6.** Scatter plot: Serum albumin and T2DM
16. **Supplementary Figure S7.** Scatter plot: Serum albumin and PHD
17. **Supplementary Figure S8.** Leave-one-out sensitivity analysis: Serum albumin and HF
18. **Supplementary Figure S9.** Leave-one-out sensitivity analysis: Serum albumin and VTE
19. **Supplementary Figure S10.** Leave-one-out sensitivity analysis: Serum albumin and Stroke
20. **Supplementary Figure S11.** Leave-one-out sensitivity analysis: Serum albumin and AF
21. **Supplementary Figure S12.** Leave-one-out sensitivity analysis: Serum albumin and CAD
22. **Supplementary Figure S13.** Leave-one-out sensitivity analysis: Serum albumin and T2DM
23. **Supplementary Figure S14.** Leave-one-out sensitivity analysis: Serum albumin and PHD

**Supplementary Table S1.** Genetic instruments for serum albumin

| SNP | CHR | EA | OA | EAF | BETA | SE | P-value | F-statistic |
| --- | --- | --- | --- | --- | --- | --- | --- | --- |
| rs7366884 | 1 | C | T | 0.27 | 0.019 | 0.003 | 1.56E-11 | 45.46 |
| rs12563096 | 1 | A | G | 0.66 | 0.014 | 0.003 | 4.77E-08 | 29.81 |
| rs4970834 | 1 | T | C | 0.19 | -0.025 | 0.003 | 1.13E-15 | 64.19 |
| rs11589479 | 1 | A | G | 0.16 | 0.046 | 0.003 | 6.8E-43 | 188.54 |
| rs56188865 | 1 | C | T | 0.37 | 0.016 | 0.003 | 2.07E-10 | 40.40 |
| rs10863570 | 1 | C | T | 0.26 | 0.022 | 0.003 | 1.77E-15 | 63.31 |
| rs1986133 | 1 | T | C | 0.69 | 0.016 | 0.003 | 1.32E-09 | 36.79 |
| rs11208706 | 1 | G | T | 0.37 | 0.022 | 0.003 | 7.29E-18 | 74.14 |
| rs61817641 | 1 | T | C | 0.27 | -0.018 | 0.003 | 6.01E-11 | 42.82 |
| rs28688002 | 1 | A | T | 0.22 | 0.027 | 0.003 | 3.02E-20 | 84.99 |
| rs6682695 | 1 | C | G | 0.83 | -0.024 | 0.003 | 5.31E-13 | 52.09 |
| rs1497406 | 1 | G | A | 0.58 | 0.025 | 0.002 | 8.55E-24 | 101.16 |
| rs3768321 | 1 | T | G | 0.20 | -0.028 | 0.003 | 4.75E-20 | 84.10 |
| rs1782455 | 1 | A | G | 0.84 | 0.020 | 0.003 | 3.55E-09 | 34.86 |
| rs34596921 | 1 | TA | T | 0.42 | -0.019 | 0.003 | 1.47E-13 | 54.61 |
| rs11264233 | 1 | A | G | 0.49 | 0.019 | 0.002 | 3.83E-15 | 61.79 |
| rs10919543 | 1 | G | A | 0.32 | -0.019 | 0.003 | 6.56E-13 | 51.67 |
| rs6693993 | 1 | C | G | 0.53 | 0.024 | 0.002 | 1.57E-21 | 90.83 |
| rs2820446 | 1 | G | C | 0.30 | -0.024 | 0.003 | 4.11E-19 | 79.83 |
| rs76006845 | 2 | G | GT | 0.73 | -0.016 | 0.003 | 1.25E-08 | 32.41 |
| rs6734238 | 2 | G | A | 0.40 | -0.016 | 0.003 | 1.74E-10 | 40.74 |
| rs71010816 | 2 | GT | G | 0.75 | -0.017 | 0.003 | 1E-08 | 32.84 |
| rs59916403 | 2 | T | G | 0.35 | -0.024 | 0.003 | 3.99E-21 | 88.99 |
| rs11895352 | 2 | T | C | 0.48 | -0.014 | 0.002 | 3.11E-08 | 30.64 |
| rs55724869 | 2 | C | G | 0.41 | 0.014 | 0.003 | 1.46E-08 | 32.11 |
| rs7591567 | 2 | C | T | 0.29 | 0.016 | 0.003 | 7.66E-09 | 33.36 |
| rs1260326 | 2 | C | T | 0.61 | -0.059 | 0.003 | 1.5E-121 | 550.14 |
| rs4499445 | 2 | T | G | 0.65 | -0.022 | 0.003 | 6.94E-17 | 69.70 |
| rs2972145 | 2 | C | T | 0.65 | 0.021 | 0.003 | 8.16E-17 | 69.38 |
| rs17023530 | 2 | A | T | 0.05 | -0.038 | 0.006 | 2.53E-11 | 44.52 |
| rs13389219 | 2 | T | C | 0.39 | -0.029 | 0.003 | 2.07E-31 | 135.96 |
| rs1801282 | 3 | G | C | 0.12 | -0.051 | 0.004 | 4.57E-41 | 180.16 |
| rs6549406 | 3 | G | A | 0.73 | -0.019 | 0.003 | 1E-11 | 46.32 |
| rs113177823 | 3 | A | G | 0.05 | 0.043 | 0.005 | 6.59E-15 | 60.72 |
| rs6793835 | 3 | A | G | 0.26 | 0.016 | 0.003 | 4.69E-09 | 34.32 |
| rs11928797 | 3 | A | C | 0.12 | 0.028 | 0.004 | 3.35E-13 | 52.99 |
| rs74780677 | 3 | G | A | 0.02 | 0.093 | 0.010 | 3.21E-20 | 84.87 |
| rs6794370 | 3 | C | A | 0.81 | -0.019 | 0.003 | 7.27E-10 | 37.95 |
| rs9917677 | 3 | C | T | 0.25 | -0.016 | 0.003 | 4.25E-08 | 30.03 |
| rs900400 | 3 | C | T | 0.40 | 0.023 | 0.003 | 3.37E-19 | 80.22 |
| rs667172 | 3 | A | G | 0.29 | 0.019 | 0.003 | 3.81E-12 | 48.23 |
| rs234043 | 3 | C | T | 0.72 | 0.017 | 0.003 | 6.08E-10 | 38.30 |
| rs77849807 | 4 | G | A | 0.02 | 0.114 | 0.010 | 1.31E-30 | 132.28 |
| rs10004084 | 4 | C | T | 0.17 | 0.030 | 0.003 | 1.51E-19 | 81.81 |
| rs13107325 | 4 | T | C | 0.07 | -0.056 | 0.005 | 7.81E-33 | 142.47 |
| rs4833945 | 4 | C | T | 0.12 | 0.022 | 0.004 | 1.79E-08 | 31.71 |
| rs150783681 | 4 | C | G | 0.02 | -0.119 | 0.009 | 2.77E-42 | 185.76 |
| rs1229984 | 4 | C | T | 0.98 | 0.060 | 0.008 | 7.87E-13 | 51.32 |
| rs13108218 | 4 | G | A | 0.62 | -0.044 | 0.003 | 3.01E-67 | 300.32 |
| rs2702571 | 4 | A | T | 0.65 | 0.017 | 0.003 | 8.12E-11 | 42.23 |
| rs2200061 | 4 | A | G | 0.21 | -0.017 | 0.003 | 4.33E-08 | 30.00 |
| rs13111128 | 4 | A | G | 0.30 | -0.019 | 0.003 | 6.99E-12 | 47.04 |
| rs28687959 | 4 | T | C | 0.56 | 0.016 | 0.002 | 3.4E-11 | 43.93 |
| rs71587365 | 4 | ATT | A | 0.59 | -0.019 | 0.003 | 1.43E-13 | 54.67 |
| rs1593357 | 4 | T | C | 0.25 | -0.020 | 0.003 | 1.18E-12 | 50.52 |
| rs390801 | 4 | C | T | 0.26 | 0.021 | 0.003 | 3.22E-14 | 57.60 |
| rs11736842 | 4 | T | A | 0.64 | 0.016 | 0.003 | 4.79E-10 | 38.76 |
| rs6871748 | 5 | C | T | 0.28 | 0.015 | 0.003 | 3.36E-08 | 30.49 |
| rs459193 | 5 | G | A | 0.75 | 0.023 | 0.003 | 1.5E-14 | 59.11 |
| rs10213692 | 5 | C | T | 0.24 | 0.020 | 0.003 | 7.54E-12 | 46.88 |
| rs2227827 | 5 | T | C | 0.05 | -0.051 | 0.006 | 5.99E-19 | 79.08 |
| rs34783842 | 5 | TA | T | 0.47 | 0.041 | 0.002 | 2.38E-61 | 273.24 |
| rs7731045 | 5 | C | T | 0.39 | -0.014 | 0.003 | 2.23E-08 | 31.29 |
| rs61552236 | 5 | ACT | A | 0.27 | -0.016 | 0.003 | 6.64E-09 | 33.64 |
| rs72818989 | 5 | C | T | 0.54 | 0.016 | 0.002 | 2.21E-10 | 40.27 |
| rs35676551 | 5 | A | C | 0.06 | -0.038 | 0.006 | 7.92E-12 | 46.79 |
| rs1500187 | 5 | G | A | 0.46 | -0.024 | 0.002 | 9.44E-22 | 91.85 |
| rs6860245 | 5 | C | G | 0.25 | -0.021 | 0.003 | 6.54E-14 | 56.20 |
| rs10042492 | 5 | T | C | 0.44 | -0.015 | 0.002 | 2.7E-09 | 35.39 |
| rs6897617 | 5 | A | G | 0.29 | 0.018 | 0.003 | 1.84E-11 | 45.14 |
| rs1998528 | 6 | A | G | 0.35 | 0.015 | 0.003 | 3.98E-09 | 34.63 |
| rs9265945 | 6 | G | A | 0.43 | -0.023 | 0.003 | 6.83E-21 | 87.93 |
| rs59842359 | 6 | T | C | 0.20 | -0.023 | 0.003 | 2.07E-13 | 53.95 |
| rs12215904 | 6 | T | C | 0.18 | -0.020 | 0.003 | 4.51E-10 | 38.88 |
| rs9391997 | 6 | G | A | 0.53 | -0.016 | 0.002 | 5.93E-11 | 42.85 |
| rs4327724 | 6 | T | C | 0.08 | 0.031 | 0.005 | 9.62E-12 | 46.41 |
| rs10456852 | 6 | T | C | 0.13 | 0.026 | 0.004 | 1.12E-12 | 50.62 |
| rs72959041 | 6 | A | G | 0.05 | 0.051 | 0.006 | 2.94E-19 | 80.49 |
| rs9262066 | 6 | T | G | 0.62 | 0.015 | 0.003 | 7.53E-09 | 33.40 |
| rs4711399 | 6 | T | C | 0.78 | 0.022 | 0.003 | 1.29E-13 | 54.87 |
| rs1331309 | 6 | G | T | 0.26 | 0.021 | 0.003 | 1.23E-13 | 54.96 |
| rs6912315 | 6 | T | C | 0.05 | 0.031 | 0.005 | 1.16E-08 | 32.55 |
| rs4946811 | 6 | C | A | 0.36 | 0.016 | 0.003 | 5.92E-10 | 38.35 |
| rs2115868 | 6 | A | T | 0.16 | -0.024 | 0.003 | 3.03E-13 | 53.19 |
| rs1880241 | 7 | G | A | 0.49 | 0.016 | 0.002 | 2.85E-10 | 39.77 |
| rs2710804 | 7 | C | T | 0.38 | -0.016 | 0.003 | 9.55E-11 | 41.91 |
| rs4410790 | 7 | C | T | 0.63 | 0.015 | 0.003 | 4.14E-09 | 34.56 |
| rs60644673 | 7 | T | G | 0.19 | -0.018 | 0.003 | 1.92E-08 | 31.58 |
| rs114949263 | 7 | C | T | 0.11 | 0.057 | 0.004 | 7.29E-48 | 211.33 |
| rs149092986 | 7 | C | T | 0.02 | -0.055 | 0.008 | 5.07E-11 | 43.16 |
| rs157936 | 7 | G | T | 0.30 | -0.019 | 0.003 | 1.61E-12 | 49.91 |
| rs10236582 | 7 | C | T | 0.28 | -0.022 | 0.003 | 9.96E-16 | 64.44 |
| rs6970593 | 7 | A | G | 0.49 | -0.026 | 0.002 | 5.84E-25 | 106.49 |
| rs9638180 | 7 | G | A | 0.20 | -0.024 | 0.003 | 2.47E-15 | 62.66 |
| rs1229492 | 7 | C | T | 0.73 | -0.017 | 0.003 | 1.08E-09 | 37.18 |
| rs73234873 | 7 | T | C | 0.29 | -0.015 | 0.003 | 3.22E-08 | 30.57 |
| rs2072442 | 7 | G | C | 0.57 | 0.015 | 0.002 | 5.51E-09 | 34.00 |
| rs67694436 | 8 | T | C | 0.36 | 0.015 | 0.003 | 5E-09 | 34.19 |
| rs800545 | 8 | G | A | 0.75 | -0.016 | 0.003 | 1.15E-08 | 32.57 |
| rs58579887 | 8 | C | T | 0.40 | 0.018 | 0.003 | 3.43E-13 | 52.95 |
| rs2169387 | 8 | G | A | 0.90 | 0.058 | 0.004 | 1.4E-44 | 196.28 |
| rs55846720 | 8 | A | G | 0.57 | -0.015 | 0.002 | 2.4E-09 | 35.62 |
| rs28601761 | 8 | G | C | 0.42 | -0.020 | 0.003 | 2.53E-15 | 62.61 |
| rs56094005 | 8 | G | A | 0.04 | -0.047 | 0.006 | 1.08E-14 | 59.75 |
| rs4458838 | 8 | A | G | 0.37 | -0.016 | 0.003 | 1.3E-09 | 36.82 |
| rs73263719 | 8 | A | G | 0.17 | 0.019 | 0.003 | 2.68E-08 | 30.93 |
| rs1079290 | 9 | A | T | 0.42 | 0.015 | 0.002 | 5.75E-10 | 38.41 |
| rs12377600 | 9 | G | A | 0.35 | -0.018 | 0.003 | 2.11E-12 | 49.39 |
| rs930340 | 9 | A | G | 0.81 | -0.019 | 0.003 | 2.32E-09 | 35.69 |
| rs378740 | 9 | A | C | 0.27 | 0.016 | 0.003 | 1.78E-08 | 31.72 |
| rs7031621 | 9 | A | G | 0.50 | -0.014 | 0.002 | 5.18E-09 | 34.12 |
| rs473919 | 10 | G | C | 0.20 | -0.019 | 0.003 | 1.5E-09 | 36.54 |
| rs74502455 | 10 | G | C | 0.48 | -0.021 | 0.002 | 2.49E-17 | 71.72 |
| rs3099371 | 10 | T | C | 0.59 | 0.016 | 0.003 | 2.65E-10 | 39.92 |
| rs11012732 | 10 | G | A | 0.33 | -0.023 | 0.003 | 5.39E-19 | 79.29 |
| rs36090025 | 10 | C | A | 0.30 | 0.017 | 0.003 | 9.74E-11 | 41.88 |
| rs10793127 | 11 | A | G | 0.09 | 0.025 | 0.004 | 8.05E-09 | 33.26 |
| rs11217135 | 11 | T | C | 0.45 | -0.024 | 0.002 | 5.01E-23 | 97.67 |
| rs117127664 | 11 | T | C | 0.04 | 0.038 | 0.006 | 1.65E-09 | 36.35 |
| rs631695 | 11 | G | T | 0.58 | 0.015 | 0.002 | 5.28E-10 | 38.57 |
| rs2060658 | 11 | C | T | 0.55 | 0.017 | 0.002 | 2.16E-11 | 44.83 |
| rs3740688 | 11 | T | G | 0.54 | 0.024 | 0.002 | 1.47E-22 | 95.53 |
| rs74538877 | 11 | C | G | 0.06 | 0.031 | 0.005 | 1.51E-08 | 32.05 |
| rs1791936 | 11 | A | G | 0.61 | 0.021 | 0.003 | 2.31E-17 | 71.87 |
| rs2785172 | 11 | A | G | 0.61 | -0.019 | 0.003 | 3.49E-14 | 57.45 |
| rs198426 | 11 | T | C | 0.34 | -0.017 | 0.003 | 8.75E-11 | 42.08 |
| rs673751 | 11 | C | A | 0.68 | -0.015 | 0.003 | 6.77E-09 | 33.60 |
| rs45439091 | 11 | T | G | 0.07 | 0.032 | 0.005 | 2.38E-11 | 44.64 |
| rs55696240 | 11 | A | G | 0.38 | 0.017 | 0.003 | 2.42E-11 | 44.61 |
| rs11609805 | 12 | A | G | 0.24 | -0.025 | 0.003 | 7.52E-19 | 78.63 |
| rs3184504 | 12 | C | T | 0.52 | 0.021 | 0.002 | 3.81E-18 | 75.43 |
| rs79295634 | 12 | G | A | 0.07 | 0.031 | 0.005 | 2.22E-10 | 40.26 |
| rs12815728 | 12 | C | T | 0.54 | 0.018 | 0.002 | 2.92E-13 | 53.26 |
| rs76895963 | 12 | G | T | 0.02 | -0.071 | 0.009 | 4.8E-14 | 56.82 |
| rs78444263 | 12 | T | C | 0.07 | -0.035 | 0.005 | 2.11E-13 | 53.90 |
| rs2638315 | 12 | C | G | 0.18 | 0.035 | 0.003 | 1.07E-28 | 123.56 |
| rs10431419 | 12 | A | C | 0.22 | -0.017 | 0.003 | 2.21E-08 | 31.31 |
| rs11057273 | 12 | C | T | 0.91 | -0.029 | 0.004 | 1.55E-11 | 45.48 |
| rs992367 | 13 | G | A | 0.60 | 0.015 | 0.003 | 1.04E-09 | 37.25 |
| rs6490409 | 13 | G | A | 0.14 | -0.019 | 0.004 | 3.17E-08 | 30.61 |
| rs10143115 | 14 | A | G | 0.54 | -0.015 | 0.003 | 4.62E-09 | 34.35 |
| rs75070309 | 14 | A | C | 0.18 | 0.032 | 0.003 | 4.02E-23 | 98.10 |
| rs12881869 | 14 | T | C | 0.07 | -0.049 | 0.005 | 4.9E-25 | 106.83 |
| rs28929474 | 14 | T | C | 0.02 | 0.265 | 0.009 | 1E-200 | 921.58 |
| rs17580 | 14 | A | T | 0.05 | 0.095 | 0.006 | 7.81E-62 | 275.45 |
| rs72694393 | 14 | T | G | 0.48 | 0.018 | 0.002 | 1.6E-13 | 54.44 |
| rs61983272 | 14 | C | G | 0.32 | -0.033 | 0.003 | 7.26E-31 | 133.47 |
| rs139974673 | 15 | C | T | 0.03 | 0.151 | 0.008 | 4.34E-82 | 368.55 |
| rs1532085 | 15 | G | A | 0.61 | 0.014 | 0.003 | 2.06E-08 | 31.44 |
| rs59431480 | 15 | G | C | 0.01 | 0.086 | 0.013 | 1.6E-11 | 45.41 |
| rs2869876 | 15 | A | C | 0.18 | 0.018 | 0.003 | 1.86E-08 | 31.64 |
| rs7402977 | 15 | A | G | 0.27 | -0.016 | 0.003 | 6.44E-09 | 33.70 |
| rs58558667 | 15 | T | G | 0.37 | 0.023 | 0.003 | 1.81E-19 | 81.45 |
| rs8041057 | 15 | T | C | 0.71 | -0.023 | 0.003 | 1.45E-17 | 72.79 |
| rs872926 | 15 | A | G | 0.21 | -0.036 | 0.003 | 4.05E-32 | 139.19 |
| rs72789541 | 16 | A | T | 0.30 | 0.018 | 0.003 | 1.52E-11 | 45.51 |
| rs78961851 | 16 | C | T | 0.10 | -0.025 | 0.004 | 1.18E-09 | 37.01 |
| rs879620 | 16 | T | C | 0.62 | -0.014 | 0.003 | 2.33E-08 | 31.20 |
| rs72793380 | 16 | A | C | 0.11 | 0.022 | 0.004 | 2.47E-08 | 31.09 |
| rs4782568 | 16 | G | C | 0.45 | -0.018 | 0.002 | 1.31E-13 | 54.84 |
| rs12924886 | 16 | T | A | 0.19 | 0.029 | 0.003 | 7.37E-21 | 87.78 |
| rs11074901 | 16 | A | G | 0.31 | 0.015 | 0.003 | 3.22E-08 | 30.57 |
| rs16950612 | 16 | G | A | 0.11 | -0.025 | 0.004 | 1.1E-10 | 41.63 |
| rs4796307 | 17 | A | T | 0.79 | -0.028 | 0.003 | 6.7E-21 | 87.96 |
| rs117820542 | 17 | G | C | 0.03 | -0.041 | 0.007 | 3.29E-08 | 30.53 |
| rs1030098 | 17 | C | T | 0.38 | 0.020 | 0.003 | 1.19E-15 | 64.09 |
| rs34562254 | 17 | A | G | 0.10 | -0.038 | 0.004 | 1.2E-19 | 82.26 |
| rs11656541 | 17 | C | G | 0.65 | 0.027 | 0.003 | 3.69E-25 | 107.39 |
| rs4790875 | 17 | T | C | 0.59 | -0.021 | 0.003 | 1.71E-16 | 67.92 |
| rs8072215 | 17 | G | A | 0.23 | -0.034 | 0.003 | 2.96E-31 | 135.25 |
| rs111443054 | 17 | TCACACA | T | 0.18 | -0.028 | 0.003 | 4.04E-19 | 79.86 |
| rs77542162 | 17 | G | A | 0.02 | -0.193 | 0.008 | 1.8E-122 | 554.39 |
| rs72631343 | 17 | G | C | 0.13 | 0.022 | 0.004 | 4.63E-09 | 34.34 |
| rs11078597 | 17 | C | T | 0.19 | 0.065 | 0.003 | 1.65E-95 | 430.25 |
| rs62053895 | 17 | A | G | 0.41 | -0.014 | 0.003 | 3.83E-08 | 30.23 |
| rs201931078 | 17 | GTGT | G | 0.30 | -0.017 | 0.003 | 4.89E-10 | 38.72 |
| rs34931250 | 17 | T | C | 0.06 | 0.033 | 0.005 | 7.66E-11 | 42.35 |
| rs854796 | 17 | A | G | 0.68 | 0.015 | 0.003 | 5.59E-09 | 33.98 |
| rs6567095 | 18 | A | G | 0.55 | 0.019 | 0.002 | 5.46E-15 | 61.09 |
| rs55722786 | 18 | T | G | 0.29 | 0.028 | 0.003 | 7.79E-25 | 105.91 |
| rs147651823 | 19 | A | C | 0.05 | -0.035 | 0.006 | 3.03E-09 | 35.17 |
| rs45512696 | 19 | T | C | 0.18 | 0.080 | 0.003 | 2.7E-135 | 613.47 |
| rs138833981 | 19 | G | C | 0.02 | -0.086 | 0.010 | 4.19E-18 | 75.24 |
| rs2304130 | 19 | G | A | 0.09 | 0.027 | 0.004 | 7.3E-10 | 37.94 |
| rs73038384 | 19 | T | C | 0.03 | -0.052 | 0.007 | 1.18E-13 | 55.05 |
| rs4805129 | 19 | C | T | 0.63 | -0.021 | 0.003 | 1.13E-16 | 68.74 |
| rs4805881 | 19 | C | A | 0.67 | -0.027 | 0.003 | 3.6E-25 | 107.44 |
| rs10419198 | 19 | T | C | 0.25 | -0.074 | 0.003 | 5.6E-149 | 676.48 |
| rs4804413 | 19 | T | C | 0.43 | 0.018 | 0.002 | 1.54E-12 | 49.99 |
| rs2303695 | 19 | T | C | 0.35 | -0.026 | 0.003 | 2.81E-23 | 98.80 |
| rs139278099 | 19 | T | C | 0.04 | 0.063 | 0.006 | 4.47E-24 | 102.45 |
| rs6031847 | 20 | T | C | 0.27 | -0.021 | 0.003 | 1.88E-14 | 58.65 |
| rs2267867 | 20 | G | A | 0.24 | -0.016 | 0.003 | 2.75E-08 | 30.88 |
| rs3810484 | 20 | G | A | 0.44 | 0.015 | 0.002 | 2.43E-09 | 35.60 |
| rs1886839 | 20 | G | A | 0.60 | 0.014 | 0.003 | 2.04E-08 | 31.46 |
| rs6065295 | 20 | T | C | 0.42 | 0.015 | 0.002 | 1.65E-09 | 36.35 |
| rs35123414 | 20 | C | T | 0.13 | 0.022 | 0.004 | 2.1E-09 | 35.89 |
| rs11088253 | 21 | T | C | 0.46 | 0.014 | 0.002 | 2.4E-08 | 31.14 |
| rs9976946 | 21 | T | C | 0.96 | 0.039 | 0.006 | 1.47E-09 | 36.58 |
| rs1110659 | 22 | C | T | 0.24 | 0.022 | 0.003 | 2.39E-14 | 58.19 |
| rs12710562 | 22 | G | A | 0.18 | 0.026 | 0.003 | 1.51E-16 | 68.16 |
| rs62235071 | 22 | T | C | 0.06 | -0.040 | 0.005 | 1.48E-14 | 59.13 |
| rs1043312 | 22 | G | T | 0.35 | 0.014 | 0.003 | 3.89E-08 | 30.21 |

SNPs, single nucleotide polymorphisms; CHR, chromosome; EA, effect allele; OA, other allele; EAF, EA frequency; BETA, change in standardized serum albumin measure by allele; SE, standard error

**Supplementary Table S2.** SNP serum albumin and SNP HF associations (per Effect Allele) of the instruments of serum albumin after data harmonization and outlier removal

| SNP | SNP-Serum Albumin | | | | SNP-HF | | | |
| --- | --- | --- | --- | --- | --- | --- | --- | --- |
|  | EA | OA | Beta | SE | EA | OA | Beta | SE |
| rs10004084 | C | T | 0.030092 | 0.003327 | C | T | -6.5E-05 | 0.000198 |
| rs10042492 | T | C | -0.014739 | 0.0024776 | T | C | -4E-05 | 0.000147 |
| rs10143115 | A | G | -0.014661 | 0.0025016 | A | G | 6.89E-05 | 0.000149 |
| rs10213692 | C | T | 0.019841 | 0.0028977 | C | T | -0.0002 | 0.000172 |
| rs10236582 | C | T | -0.022167 | 0.0027613 | C | T | 0.000109 | 0.000164 |
| rs1030098 | C | T | 0.020357 | 0.0025429 | C | T | 0.000153 | 0.000151 |
| rs10419198 | T | C | -0.073908 | 0.0028416 | T | C | -5.7E-05 | 0.000169 |
| rs10431419 | A | C | -0.016538 | 0.0029557 | A | C | 0.000139 | 0.000176 |
| rs1043312 | G | T | 0.014211 | 0.0025857 | G | T | -1.4E-05 | 0.000154 |
| rs10456852 | T | C | 0.026322 | 0.0036997 | T | C | -0.0003 | 0.00022 |
| rs1079290 | A | T | 0.015474 | 0.0024969 | A | T | -1.6E-05 | 0.000148 |
| rs10793127 | A | G | 0.024629 | 0.0042703 | A | G | -1.2E-05 | 0.000254 |
| rs10863570 | C | T | 0.022492 | 0.0028268 | C | T | -9.6E-05 | 0.000168 |
| rs10919543 | G | A | -0.019189 | 0.0026694 | G | A | 3.27E-05 | 0.000159 |
| rs11012732 | G | A | -0.023341 | 0.0026213 | G | A | 9.08E-06 | 0.000156 |
| rs11057273 | C | T | -0.028641 | 0.0042471 | C | T | -0.00035 | 0.000252 |
| rs11074901 | A | G | 0.01473 | 0.0026641 | A | G | -9.4E-05 | 0.000158 |
| rs11078597 | C | T | 0.065424 | 0.0031541 | C | T | -7.9E-05 | 0.000188 |
| rs11088253 | T | C | 0.013768 | 0.0024673 | T | C | 0.000278 | 0.000147 |
| rs1110659 | C | T | 0.02205 | 0.0028907 | C | T | -0.00018 | 0.000172 |
| rs111443054 | TCACACA | T | -0.028485 | 0.0031875 | AGT | A | -4.7E-05 | 0.000265 |
| rs111443054 | TCACACA | T | -0.028485 | 0.0031875 | TCACACA | T | -7.7E-05 | 0.00019 |
| rs11208706 | G | T | 0.021997 | 0.0025546 | G | T | 0.000229 | 0.000152 |
| rs11217135 | T | C | -0.024493 | 0.0024784 | T | C | -0.00031 | 0.000147 |
| rs11264233 | A | G | 0.019323 | 0.0024581 | A | G | -0.00015 | 0.000146 |
| rs113177823 | A | G | 0.0428 | 0.0054926 | A | G | 0.000368 | 0.000326 |
| rs114949263 | C | T | 0.056977 | 0.0039194 | C | T | 0.00028 | 0.000233 |
| rs11589479 | A | G | 0.045527 | 0.0033156 | A | G | -0.00022 | 0.000197 |
| rs11609805 | A | G | -0.02541 | 0.0028656 | A | G | -0.00052 | 0.00017 |
| rs11656541 | C | G | 0.026835 | 0.0025895 | C | G | -0.00021 | 0.000154 |
| rs117127664 | T | C | 0.037526 | 0.0062244 | T | C | -0.00094 | 0.00037 |
| rs11736842 | T | A | 0.015911 | 0.0025556 | T | A | -3E-05 | 0.000152 |
| rs117820542 | G | C | -0.041143 | 0.0074459 | G | C | 0.000545 | 0.000443 |
| rs11895352 | T | C | -0.013656 | 0.0024671 | T | C | 0.000149 | 0.000147 |
| rs11928797 | A | C | 0.027873 | 0.0038289 | A | C | 0.000109 | 0.000228 |
| rs12215904 | T | C | -0.01996 | 0.0032011 | T | C | -2.9E-05 | 0.00019 |
| rs1229492 | C | T | -0.017075 | 0.0028003 | C | T | -0.00016 | 0.000167 |
| rs1229984 | C | T | 0.059701 | 0.0083338 | C | T | 0.000472 | 0.000495 |
| rs12377600 | G | A | -0.018159 | 0.002584 | G | A | -0.00012 | 0.000154 |
| rs12563096 | A | G | 0.01427 | 0.0026138 | A | G | 4.93E-06 | 0.000155 |
| rs1260326 | C | T | -0.059001 | 0.0025155 | C | T | -0.00017 | 0.00015 |
| rs12710562 | G | A | 0.026315 | 0.0031873 | G | A | 0.000269 | 0.00019 |
| rs12815728 | C | T | 0.018075 | 0.0024767 | C | T | -5.9E-05 | 0.000147 |
| rs12881869 | T | C | -0.04948 | 0.0047873 | T | C | -0.00037 | 0.000285 |
| rs12924886 | T | A | 0.029495 | 0.0031482 | T | A | -0.00014 | 0.000187 |
| rs13107325 | T | C | -0.055886 | 0.0046821 | T | C | 0.000115 | 0.000278 |
| rs13108218 | G | A | -0.044248 | 0.0025533 | G | A | -0.0002 | 0.000152 |
| rs13111128 | A | G | -0.018612 | 0.0027138 | A | G | -4.9E-05 | 0.000161 |
| rs1331309 | G | T | 0.020886 | 0.0028173 | G | T | -5.2E-05 | 0.000167 |
| rs13389219 | T | C | -0.029364 | 0.0025183 | T | C | 1.92E-05 | 0.00015 |
| rs138833981 | G | C | -0.085809 | 0.0098927 | G | C | -0.00026 | 0.000589 |
| rs139278099 | T | C | 0.062656 | 0.0061903 | T | C | -0.00042 | 0.000368 |
| rs139974673 | C | T | 0.15127 | 0.0078796 | C | T | -8.3E-05 | 0.00047 |
| rs147651823 | A | C | -0.034933 | 0.0058906 | A | C | 0.000233 | 0.00035 |
| rs149092986 | C | T | -0.054896 | 0.0083565 | C | T | -0.00049 | 0.000497 |
| rs1497406 | G | A | 0.025056 | 0.0024912 | G | A | 0.000139 | 0.000148 |
| rs1500187 | G | A | -0.023694 | 0.0024723 | G | A | 8.48E-05 | 0.000147 |
| rs150783681 | C | G | -0.11931 | 0.0087539 | C | G | 0.000205 | 0.000521 |
| rs1532085 | G | A | 0.014174 | 0.0025278 | G | A | -0.00011 | 0.00015 |
| rs157936 | G | T | -0.019043 | 0.0026956 | G | T | -0.00045 | 0.00016 |
| rs1593357 | T | C | -0.020357 | 0.002864 | T | C | 0.000106 | 0.00017 |
| rs16950612 | G | A | -0.025444 | 0.0039433 | G | A | 0.000211 | 0.000235 |
| rs17023530 | A | T | -0.038379 | 0.0057521 | A | T | 8.92E-07 | 0.000342 |
| rs17580 | A | T | 0.094752 | 0.0057091 | A | T | -0.00025 | 0.00034 |
| rs1782455 | A | G | 0.019719 | 0.0033399 | A | G | 0.000105 | 0.000199 |
| rs1791936 | A | G | 0.021346 | 0.002518 | A | G | 1.92E-05 | 0.00015 |
| rs1801282 | G | C | -0.050658 | 0.0037741 | G | C | 0.000109 | 0.000224 |
| rs1880241 | G | A | 0.015573 | 0.0024693 | G | A | 4.53E-06 | 0.000147 |
| rs1886839 | G | A | 0.014096 | 0.0025133 | G | A | 0.000348 | 0.000149 |
| rs198426 | T | C | -0.016913 | 0.0026071 | T | C | -3.6E-05 | 0.000155 |
| rs1986133 | T | C | 0.016108 | 0.0026558 | T | C | -0.00012 | 0.000158 |
| rs1998528 | A | G | 0.015217 | 0.0025857 | A | G | -6.9E-05 | 0.000154 |
| rs201931078 | GTGT | G | -0.016835 | 0.0027054 | GTGT | G | -4.1E-06 | 0.000161 |
| rs2060658 | C | T | 0.016588 | 0.0024776 | C | T | 0.000131 | 0.000147 |
| rs2072442 | G | C | 0.014529 | 0.0024917 | G | C | -0.00013 | 0.000148 |
| rs2115868 | A | T | -0.024382 | 0.003343 | A | T | 0.000165 | 0.000199 |
| rs2169387 | G | A | 0.057547 | 0.0041076 | G | A | -4.3E-05 | 0.000244 |
| rs2200061 | A | G | -0.016504 | 0.0030134 | A | G | -8.1E-06 | 0.000179 |
| rs2227827 | T | C | -0.051242 | 0.0057622 | T | C | 0.000247 | 0.000342 |
| rs2267867 | G | A | -0.016091 | 0.0028957 | G | A | 1.97E-05 | 0.000172 |
| rs2303695 | T | C | -0.025668 | 0.0025823 | T | C | 0.000202 | 0.000154 |
| rs2304130 | G | A | 0.02707 | 0.0043948 | G | A | -0.00049 | 0.000261 |
| rs234043 | C | T | 0.016912 | 0.0027328 | C | T | 2.14E-05 | 0.000162 |
| rs2638315 | C | G | 0.035351 | 0.0031803 | C | G | -0.0003 | 0.000189 |
| rs2702571 | A | T | 0.016766 | 0.0025799 | A | T | -0.00024 | 0.000153 |
| rs2710804 | C | T | -0.016474 | 0.0025446 | C | T | -1.8E-05 | 0.000151 |
| rs2785172 | A | G | -0.019156 | 0.0025274 | A | G | 0.000136 | 0.00015 |
| rs28601761 | G | C | -0.019962 | 0.0025228 | G | C | -0.00049 | 0.00015 |
| rs28687959 | T | C | 0.016421 | 0.0024775 | T | C | -0.00035 | 0.000147 |
| rs28688002 | A | T | 0.027203 | 0.0029507 | A | T | 0.00013 | 0.000175 |
| rs2869876 | A | C | 0.017918 | 0.0031857 | A | C | -0.0002 | 0.000189 |
| rs28929474 | T | C | 0.26514 | 0.0087339 | T | C | -0.00031 | 0.000519 |
| rs2972145 | C | T | 0.021428 | 0.0025726 | C | T | 0.000339 | 0.000153 |
| rs3099371 | T | C | 0.015872 | 0.002512 | T | C | -0.0001 | 0.000149 |
| rs3184504 | C | T | 0.021388 | 0.0024627 | C | T | -4.6E-05 | 0.000146 |
| rs34562254 | A | G | -0.037537 | 0.0041386 | A | G | 0.000344 | 0.000246 |
| rs34596921 | TA | T | -0.018653 | 0.0025241 | TA | T | -0.00017 | 0.00015 |
| rs34783842 | TA | T | 0.040771 | 0.0024665 | TA | T | 3.93E-05 | 0.000147 |
| rs34931250 | T | C | 0.03341 | 0.0051342 | T | C | 0.000794 | 0.000305 |
| rs35123414 | C | T | 0.021697 | 0.0036219 | C | T | 0.000291 | 0.000216 |
| rs35676551 | A | C | -0.037895 | 0.00554 | A | C | 0.000559 | 0.000329 |
| rs36090025 | C | A | 0.017388 | 0.0026869 | C | A | 2.82E-05 | 0.00016 |
| rs3740688 | T | G | 0.024193 | 0.0024753 | T | G | -0.00016 | 0.000147 |
| rs3768321 | T | G | -0.028418 | 0.0030989 | T | G | -0.00016 | 0.000184 |
| rs378740 | A | C | 0.01565 | 0.0027789 | A | C | -2.3E-06 | 0.000165 |
| rs3810484 | G | A | 0.014777 | 0.0024766 | G | A | -0.00017 | 0.000147 |
| rs390801 | C | T | 0.021268 | 0.0028023 | C | T | 5.03E-05 | 0.000167 |
| rs4327724 | T | C | 0.031296 | 0.0045941 | T | C | 5.88E-05 | 0.000273 |
| rs4410790 | C | T | 0.015033 | 0.0025572 | C | T | 7.68E-05 | 0.000152 |
| rs4458838 | A | G | -0.015661 | 0.0025809 | A | G | 3.35E-05 | 0.000153 |
| rs4499445 | T | G | -0.021739 | 0.0026039 | T | G | -0.00017 | 0.000155 |
| rs45439091 | T | G | 0.031713 | 0.0047467 | T | G | -8.1E-05 | 0.000282 |
| rs45512696 | T | C | 0.080056 | 0.0032322 | T | C | 6.01E-05 | 0.000192 |
| rs459193 | G | A | 0.022894 | 0.0029778 | G | A | 0.000318 | 0.000177 |
| rs4711399 | T | C | 0.021968 | 0.0029656 | T | C | 0.000103 | 0.000176 |
| rs473919 | G | C | -0.018882 | 0.0031236 | G | C | 0.0001 | 0.000186 |
| rs4782568 | G | C | -0.018428 | 0.0024885 | G | C | 9.7E-06 | 0.000148 |
| rs4790875 | T | C | -0.020709 | 0.0025128 | T | C | -0.00016 | 0.000149 |
| rs4796307 | A | T | -0.028149 | 0.0030013 | A | T | 2E-06 | 0.000178 |
| rs4804413 | T | C | 0.017594 | 0.0024883 | T | C | 0.000259 | 0.000148 |
| rs4805129 | C | T | -0.021111 | 0.0025462 | C | T | 0.000146 | 0.000151 |
| rs4805881 | C | A | -0.02704 | 0.0026087 | C | A | 0.000258 | 0.000155 |
| rs4833945 | C | T | 0.021705 | 0.0038543 | C | T | 1.01E-05 | 0.000229 |
| rs4946811 | C | A | 0.015897 | 0.002567 | C | A | -0.00012 | 0.000153 |
| rs4970834 | T | C | -0.025389 | 0.0031689 | T | C | -0.0003 | 0.000188 |
| rs55696240 | A | G | 0.017035 | 0.0025506 | A | G | 6.13E-05 | 0.000152 |
| rs55722786 | T | G | 0.028148 | 0.0027352 | T | G | -7.5E-05 | 0.000163 |
| rs55724869 | C | G | 0.014174 | 0.0025012 | C | G | -9.1E-05 | 0.000149 |
| rs55846720 | A | G | -0.014796 | 0.0024791 | A | G | -0.00027 | 0.000147 |
| rs56094005 | G | A | -0.046542 | 0.0060213 | G | A | 0.000336 | 0.000358 |
| rs56188865 | C | T | 0.016247 | 0.002556 | C | T | -0.0001 | 0.000152 |
| rs58558667 | T | G | 0.023041 | 0.002553 | T | G | 7.53E-06 | 0.000152 |
| rs58579887 | C | T | 0.018348 | 0.0025215 | C | T | -5.3E-05 | 0.00015 |
| rs59431480 | G | C | 0.085838 | 0.012738 | G | C | 0.000221 | 0.000757 |
| rs59842359 | T | C | -0.022571 | 0.003073 | T | C | 0.000127 | 0.000183 |
| rs59916403 | T | G | -0.024299 | 0.0025758 | T | G | -0.00016 | 0.000153 |
| rs6031847 | T | C | -0.021265 | 0.0027766 | T | C | -9.3E-05 | 0.000165 |
| rs60644673 | T | G | -0.017542 | 0.0031218 | T | G | -0.00023 | 0.000186 |
| rs6065295 | T | C | 0.015019 | 0.0024912 | T | C | 6.73E-05 | 0.000148 |
| rs61552236 | ACT | A | -0.0161 | 0.0027759 | ACT | A | 0.000265 | 0.000165 |
| rs61817641 | T | C | -0.018206 | 0.0027823 | T | C | 5.26E-05 | 0.000165 |
| rs61983272 | C | G | -0.032856 | 0.002844 | C | G | 0.000124 | 0.000169 |
| rs62053895 | A | G | -0.013849 | 0.0025187 | A | G | -6.2E-06 | 0.00015 |
| rs62235071 | T | C | -0.040139 | 0.00522 | T | C | 0.000174 | 0.00031 |
| rs631695 | G | T | 0.015489 | 0.002494 | G | T | -6.9E-05 | 0.000148 |
| rs6490409 | G | A | -0.019425 | 0.0035112 | G | A | 9.87E-05 | 0.000209 |
| rs6549406 | G | A | -0.018975 | 0.0027879 | G | A | 1.67E-05 | 0.000166 |
| rs6567095 | A | G | 0.01941 | 0.0024834 | A | G | -8.4E-05 | 0.000148 |
| rs667172 | A | G | 0.018923 | 0.0027249 | A | G | 0.000185 | 0.000162 |
| rs6682695 | C | G | -0.023971 | 0.0033213 | C | G | -0.00031 | 0.000197 |
| rs6693993 | C | G | 0.023539 | 0.0024698 | C | G | -9.3E-05 | 0.000147 |
| rs6734238 | G | A | -0.016003 | 0.0025073 | G | A | -3.1E-05 | 0.000149 |
| rs673751 | C | A | -0.015293 | 0.0026383 | C | A | -0.0004 | 0.000157 |
| rs67694436 | T | C | 0.01512 | 0.0025859 | T | C | 5.73E-05 | 0.000154 |
| rs6793835 | A | G | 0.016418 | 0.0028027 | A | G | -0.00012 | 0.000167 |
| rs6794370 | C | A | -0.019261 | 0.0031267 | C | A | 5E-05 | 0.000186 |
| rs6860245 | C | G | -0.021427 | 0.0028581 | C | G | -0.0001 | 0.00017 |
| rs6871748 | C | T | 0.015224 | 0.0027571 | C | T | -5.5E-05 | 0.000164 |
| rs6897617 | A | G | 0.018249 | 0.0027162 | A | G | -6.9E-05 | 0.000161 |
| rs6912315 | T | C | 0.031344 | 0.005494 | T | C | -0.00026 | 0.000327 |
| rs6970593 | A | G | -0.025504 | 0.0024715 | A | G | 4.2E-06 | 0.000147 |
| rs7031621 | A | G | -0.014376 | 0.002461 | A | G | 0.000196 | 0.000146 |
| rs71010816 | GT | G | -0.016915 | 0.0029519 | GT | G | 0.000347 | 0.000175 |
| rs71587365 | ATT | A | -0.01932 | 0.0026129 | ATT | A | 0.000171 | 0.000155 |
| rs72631343 | G | C | 0.021597 | 0.0036854 | G | C | 5.42E-05 | 0.000219 |
| rs72694393 | T | G | 0.018287 | 0.0024785 | T | G | -3E-05 | 0.000147 |
| rs72789541 | A | T | 0.018236 | 0.0027032 | A | T | 5.57E-05 | 0.000161 |
| rs72793380 | A | C | 0.02248 | 0.0040318 | A | C | -0.00013 | 0.00024 |
| rs72818989 | C | T | 0.015766 | 0.0024844 | C | T | 3.92E-05 | 0.000148 |
| rs72959041 | A | G | 0.051281 | 0.005716 | A | G | -0.00058 | 0.00034 |
| rs73038384 | T | C | -0.052059 | 0.0070163 | T | C | -0.0003 | 0.000418 |
| rs73234873 | T | C | -0.014986 | 0.0027104 | T | C | -8.1E-05 | 0.000161 |
| rs73263719 | A | G | 0.019352 | 0.0034796 | A | G | -9.2E-05 | 0.000207 |
| rs7366884 | C | T | 0.018621 | 0.0027619 | C | T | -7.4E-05 | 0.000164 |
| rs7402977 | A | G | -0.016271 | 0.002803 | A | G | -1.9E-05 | 0.000167 |
| rs74502455 | G | C | -0.02087 | 0.0024643 | G | C | 0.000284 | 0.000147 |
| rs74538877 | C | G | 0.031123 | 0.0054978 | C | G | 0.000156 | 0.000327 |
| rs74780677 | G | A | 0.093012 | 0.010096 | G | A | 0.000271 | 0.0006 |
| rs75070309 | A | C | 0.03197 | 0.0032278 | A | C | -0.00048 | 0.000192 |
| rs7591567 | C | T | 0.015616 | 0.0027036 | C | T | 0.000178 | 0.000161 |
| rs76006845 | G | GT | -0.016361 | 0.0028739 | G | GT | 0.000313 | 0.000171 |
| rs76895963 | G | T | -0.071224 | 0.009449 | G | T | -0.00067 | 0.000563 |
| rs7731045 | C | T | -0.014222 | 0.0025426 | C | T | -0.00039 | 0.000151 |
| rs77542162 | G | A | -0.19345 | 0.008216 | G | A | -0.00121 | 0.000488 |
| rs77849807 | G | A | 0.11375 | 0.00989 | G | A | 0.001027 | 0.000587 |
| rs78444263 | T | C | -0.03521 | 0.0047959 | T | C | -0.00064 | 0.000285 |
| rs78961851 | C | T | -0.024799 | 0.0040766 | C | T | 5.4E-06 | 0.000242 |
| rs800545 | G | A | -0.016169 | 0.002833 | G | A | -0.00022 | 0.000168 |
| rs8041057 | T | C | -0.023221 | 0.0027217 | T | C | -9.8E-05 | 0.000162 |
| rs8072215 | G | A | -0.033891 | 0.0029142 | G | A | -0.00012 | 0.000173 |
| rs854796 | A | G | 0.015472 | 0.0026544 | A | G | 4.06E-06 | 0.000158 |
| rs872926 | A | G | -0.035827 | 0.0030367 | A | G | 0.000239 | 0.00018 |
| rs879620 | T | C | -0.014193 | 0.0025411 | T | C | -2.5E-05 | 0.000151 |
| rs900400 | C | T | 0.022534 | 0.0025159 | C | T | -0.00013 | 0.00015 |
| rs9262066 | T | G | 0.015398 | 0.0026645 | T | G | -8.5E-05 | 0.000158 |
| rs9265945 | G | A | -0.023445 | 0.0025002 | G | A | -0.00016 | 0.000149 |
| rs930340 | A | G | -0.018877 | 0.0031599 | A | G | -0.00033 | 0.000188 |
| rs9391997 | G | A | -0.016206 | 0.0024757 | G | A | -8.2E-05 | 0.000147 |
| rs9638180 | G | A | -0.024469 | 0.0030912 | G | A | -0.00022 | 0.000184 |
| rs9917677 | C | T | -0.015506 | 0.0028295 | C | T | -1.9E-05 | 0.000168 |
| rs992367 | G | A | 0.015386 | 0.0025211 | G | A | -0.00012 | 0.00015 |
| rs9976946 | T | C | 0.038971 | 0.0064436 | T | C | 0.000213 | 0.000382 |

SNP, single nucleotide polymorphism; HF, heart failure; EA, effect allele; OA, other allele; SE, standard error

**Supplementary Table S3.** SNP serum albumin and SNP VTE associations (per Effect Allele) of the instruments of serum albumin after data harmonization and outlier removal

| SNP | SNP-Serum Albumin | | | | SNP-VTE | | | |
| --- | --- | --- | --- | --- | --- | --- | --- | --- |
|  | EA | OA | Beta | SE | EA | OA | Beta | SE |
| rs10004084 | C | T | 0.030092 | 0.003327 | C | T | 0.0002 | 0.000357 |
| rs10042492 | T | C | -0.014739 | 0.002478 | T | C | 8E-05 | 0.000266 |
| rs10143115 | A | G | -0.014661 | 0.002502 | A | G | -2E-04 | 0.000269 |
| rs10213692 | C | T | 0.019841 | 0.002898 | C | T | 7E-05 | 0.000311 |
| rs10236582 | C | T | -0.022167 | 0.002761 | C | T | 0.0002 | 0.000296 |
| rs1030098 | C | T | 0.020357 | 0.002543 | C | T | -7E-05 | 0.000273 |
| rs10419198 | T | C | -0.073908 | 0.002842 | T | C | 8E-05 | 0.000305 |
| rs10431419 | A | C | -0.016538 | 0.002956 | A | C | -1E-04 | 0.000318 |
| rs1043312 | G | T | 0.014211 | 0.002586 | G | T | -9E-05 | 0.000278 |
| rs10456852 | T | C | 0.026322 | 0.0037 | T | C | -7E-04 | 0.000397 |
| rs1079290 | A | T | 0.015474 | 0.002497 | A | T | 6E-05 | 0.000268 |
| rs10793127 | A | G | 0.024629 | 0.00427 | A | G | 0.0002 | 0.000459 |
| rs10863570 | C | T | 0.022492 | 0.002827 | C | T | -6E-05 | 0.000303 |
| rs10919543 | G | A | -0.019189 | 0.002669 | G | A | 0.0001 | 0.000287 |
| rs11012732 | G | A | -0.023341 | 0.002621 | G | A | 0.0009 | 0.000281 |
| rs11057273 | C | T | -0.028641 | 0.004247 | C | T | -4E-04 | 0.000456 |
| rs11074901 | A | G | 0.01473 | 0.002664 | A | G | -2E-04 | 0.000286 |
| rs11078597 | C | T | 0.065424 | 0.003154 | C | T | 0.0002 | 0.000339 |
| rs11088253 | T | C | 0.013768 | 0.002467 | T | C | 6E-05 | 0.000265 |
| rs1110659 | C | T | 0.02205 | 0.002891 | C | T | 0.0001 | 0.000311 |
| rs111443054 | TCACACA | T | -0.028485 | 0.003188 | TCACACA | T | 0.0008 | 0.000343 |
| rs111443054 | TCACACA | T | -0.028485 | 0.003188 | AGT | A | -4E-04 | 0.000478 |
| rs11208706 | G | T | 0.021997 | 0.002555 | G | T | 0.0005 | 0.000274 |
| rs11217135 | T | C | -0.024493 | 0.002478 | T | C | 0.0002 | 0.000266 |
| rs11264233 | A | G | 0.019323 | 0.002458 | A | G | -2E-04 | 0.000264 |
| rs113177823 | A | G | 0.0428 | 0.005493 | A | G | -3E-04 | 0.00059 |
| rs114949263 | C | T | 0.056977 | 0.003919 | C | T | -3E-04 | 0.000421 |
| rs11589479 | A | G | 0.045527 | 0.003316 | A | G | -6E-04 | 0.000356 |
| rs11609805 | A | G | -0.02541 | 0.002866 | A | G | 0.0006 | 0.000308 |
| rs11656541 | C | G | 0.026835 | 0.00259 | C | G | 0.0003 | 0.000278 |
| rs117127664 | T | C | 0.037526 | 0.006224 | T | C | -6E-04 | 0.000669 |
| rs11736842 | T | A | 0.015911 | 0.002556 | T | A | -4E-04 | 0.000275 |
| rs117820542 | G | C | -0.041143 | 0.007446 | G | C | 0.0011 | 0.000801 |
| rs11895352 | T | C | -0.013656 | 0.002467 | T | C | 0.0006 | 0.000265 |
| rs12215904 | T | C | -0.01996 | 0.003201 | T | C | 0.0003 | 0.000344 |
| rs1229492 | C | T | -0.017075 | 0.0028 | C | T | -2E-04 | 0.000301 |
| rs1229984 | C | T | 0.059701 | 0.008334 | C | T | -0.002 | 0.000895 |
| rs12377600 | G | A | -0.018159 | 0.002584 | G | A | 0.0002 | 0.000278 |
| rs12563096 | A | G | 0.01427 | 0.002614 | A | G | 9E-05 | 0.000281 |
| rs1260326 | C | T | -0.059001 | 0.002516 | C | T | 6E-05 | 0.00027 |
| rs12710562 | G | A | 0.026315 | 0.003187 | G | A | -7E-05 | 0.000342 |
| rs12815728 | C | T | 0.018075 | 0.002477 | C | T | -4E-04 | 0.000266 |
| rs12881869 | T | C | -0.04948 | 0.004787 | T | C | 0.0014 | 0.000514 |
| rs12924886 | T | A | 0.029495 | 0.003148 | T | A | 0.0003 | 0.000338 |
| rs13107325 | T | C | -0.055886 | 0.004682 | T | C | 0.0002 | 0.000503 |
| rs13108218 | G | A | -0.044248 | 0.002553 | G | A | -4E-05 | 0.000274 |
| rs13111128 | A | G | -0.018612 | 0.002714 | A | G | -5E-05 | 0.000291 |
| rs1331309 | G | T | 0.020886 | 0.002817 | G | T | 0.0003 | 0.000302 |
| rs13389219 | T | C | -0.029364 | 0.002518 | T | C | 0.0006 | 0.000271 |
| rs138833981 | G | C | -0.085809 | 0.009893 | G | C | 0.0011 | 0.001064 |
| rs139278099 | T | C | 0.062656 | 0.00619 | T | C | -0.002 | 0.000664 |
| rs139974673 | C | T | 0.15127 | 0.00788 | C | T | -0.003 | 0.000849 |
| rs147651823 | A | C | -0.034933 | 0.005891 | A | C | 0.001 | 0.000632 |
| rs149092986 | C | T | -0.054896 | 0.008357 | C | T | 0.0008 | 0.000897 |
| rs1497406 | G | A | 0.025056 | 0.002491 | G | A | -2E-04 | 0.000268 |
| rs1500187 | G | A | -0.023694 | 0.002472 | G | A | 0.0002 | 0.000266 |
| rs150783681 | C | G | -0.11931 | 0.008754 | C | G | 0.0018 | 0.000942 |
| rs1532085 | G | A | 0.014174 | 0.002528 | G | A | 0.0002 | 0.000271 |
| rs157936 | G | T | -0.019043 | 0.002696 | G | T | -3E-04 | 0.00029 |
| rs1593357 | T | C | -0.020357 | 0.002864 | T | C | 0.0002 | 0.000308 |
| rs16950612 | G | A | -0.025444 | 0.003943 | G | A | 6E-05 | 0.000424 |
| rs17023530 | A | T | -0.038379 | 0.005752 | A | T | 0.0001 | 0.000618 |
| rs17580 | A | T | 0.094752 | 0.005709 | A | T | 0.0012 | 0.000614 |
| rs1782455 | A | G | 0.019719 | 0.00334 | A | G | -7E-04 | 0.000359 |
| rs1791936 | A | G | 0.021346 | 0.002518 | A | G | 7E-05 | 0.00027 |
| rs1801282 | G | C | -0.050658 | 0.003774 | G | C | 0.0002 | 0.000405 |
| rs1880241 | G | A | 0.015573 | 0.002469 | G | A | 0.0003 | 0.000265 |
| rs1886839 | G | A | 0.014096 | 0.002513 | G | A | -5E-04 | 0.00027 |
| rs198426 | T | C | -0.016913 | 0.002607 | T | C | 0.0003 | 0.00028 |
| rs1986133 | T | C | 0.016108 | 0.002656 | T | C | -9E-05 | 0.000285 |
| rs1998528 | A | G | 0.015217 | 0.002586 | A | G | 0.0003 | 0.000278 |
| rs201931078 | GTGT | G | -0.016835 | 0.002705 | GTGT | G | -5E-05 | 0.00029 |
| rs2060658 | C | T | 0.016588 | 0.002478 | C | T | -1E-04 | 0.000266 |
| rs2072442 | G | C | 0.014529 | 0.002492 | G | C | 0.0002 | 0.000267 |
| rs2115868 | A | T | -0.024382 | 0.003343 | A | T | 0.0004 | 0.000359 |
| rs2169387 | G | A | 0.057547 | 0.004108 | G | A | -6E-04 | 0.000441 |
| rs2200061 | A | G | -0.016504 | 0.003013 | A | G | -7E-05 | 0.000323 |
| rs2227827 | T | C | -0.051242 | 0.005762 | T | C | -2E-04 | 0.000617 |
| rs2267867 | G | A | -0.016091 | 0.002896 | G | A | 0.0002 | 0.000311 |
| rs2303695 | T | C | -0.025668 | 0.002582 | T | C | 0.0001 | 0.000278 |
| rs2304130 | G | A | 0.02707 | 0.004395 | G | A | -4E-04 | 0.000472 |
| rs234043 | C | T | 0.016912 | 0.002733 | C | T | -2E-04 | 0.000294 |
| rs2638315 | C | G | 0.035351 | 0.00318 | C | G | 9E-05 | 0.000342 |
| rs2702571 | A | T | 0.016766 | 0.00258 | A | T | 9E-05 | 0.000277 |
| rs2710804 | C | T | -0.016474 | 0.002545 | C | T | -1E-04 | 0.000273 |
| rs2785172 | A | G | -0.019156 | 0.002527 | A | G | 0.0001 | 0.000272 |
| rs2820446 | G | C | -0.023986 | 0.002685 | G | C | 0.0003 | 0.000288 |
| rs28601761 | G | C | -0.019962 | 0.002523 | G | C | 0.0002 | 0.000271 |
| rs28687959 | T | C | 0.016421 | 0.002478 | T | C | -3E-04 | 0.000266 |
| rs28688002 | A | T | 0.027203 | 0.002951 | A | T | 0.0006 | 0.000317 |
| rs2869876 | A | C | 0.017918 | 0.003186 | A | C | -4E-04 | 0.000342 |
| rs2972145 | C | T | 0.021428 | 0.002573 | C | T | 0.0004 | 0.000276 |
| rs3099371 | T | C | 0.015872 | 0.002512 | T | C | -4E-04 | 0.00027 |
| rs34562254 | A | G | -0.037537 | 0.004139 | A | G | 5E-05 | 0.000444 |
| rs34596921 | TA | T | -0.018653 | 0.002524 | TA | T | 4E-05 | 0.000271 |
| rs34783842 | TA | T | 0.040771 | 0.002467 | TA | T | -4E-04 | 0.000265 |
| rs34931250 | T | C | 0.03341 | 0.005134 | T | C | 2E-05 | 0.000552 |
| rs35123414 | C | T | 0.021697 | 0.003622 | C | T | 0.0003 | 0.000389 |
| rs35676551 | A | C | -0.037895 | 0.00554 | A | C | 0.0006 | 0.000595 |
| rs36090025 | C | A | 0.017388 | 0.002687 | C | A | -3E-04 | 0.000289 |
| rs3740688 | T | G | 0.024193 | 0.002475 | T | G | 0.0002 | 0.000266 |
| rs3768321 | T | G | -0.028418 | 0.003099 | T | G | 1E-04 | 0.000333 |
| rs378740 | A | C | 0.01565 | 0.002779 | A | C | 9E-05 | 0.000298 |
| rs3810484 | G | A | 0.014777 | 0.002477 | G | A | -4E-04 | 0.000266 |
| rs390801 | C | T | 0.021268 | 0.002802 | C | T | -2E-04 | 0.000301 |
| rs4327724 | T | C | 0.031296 | 0.004594 | T | C | 0.0001 | 0.000494 |
| rs4410790 | C | T | 0.015033 | 0.002557 | C | T | 0.0004 | 0.000275 |
| rs4458838 | A | G | -0.015661 | 0.002581 | A | G | 0.0003 | 0.000277 |
| rs4499445 | T | G | -0.021739 | 0.002604 | T | G | 6E-05 | 0.00028 |
| rs45439091 | T | G | 0.031713 | 0.004747 | T | G | 0.0006 | 0.00051 |
| rs45512696 | T | C | 0.080056 | 0.003232 | T | C | -8E-04 | 0.000347 |
| rs459193 | G | A | 0.022894 | 0.002978 | G | A | 0.0001 | 0.00032 |
| rs4711399 | T | C | 0.021968 | 0.002966 | T | C | -3E-04 | 0.000318 |
| rs473919 | G | C | -0.018882 | 0.003124 | G | C | 0.0004 | 0.000336 |
| rs4782568 | G | C | -0.018428 | 0.002489 | G | C | 0.0005 | 0.000267 |
| rs4790875 | T | C | -0.020709 | 0.002513 | T | C | 0.001 | 0.00027 |
| rs4796307 | A | T | -0.028149 | 0.003001 | A | T | 0.0007 | 0.000322 |
| rs4804413 | T | C | 0.017594 | 0.002488 | T | C | -7E-05 | 0.000267 |
| rs4805129 | C | T | -0.021111 | 0.002546 | C | T | 0.0007 | 0.000273 |
| rs4805881 | C | A | -0.02704 | 0.002609 | C | A | 0.0007 | 0.00028 |
| rs4833945 | C | T | 0.021705 | 0.003854 | C | T | -2E-05 | 0.000414 |
| rs4946811 | C | A | 0.015897 | 0.002567 | C | A | -5E-04 | 0.000276 |
| rs4970834 | T | C | -0.025389 | 0.003169 | T | C | 0.0007 | 0.00034 |
| rs55696240 | A | G | 0.017035 | 0.002551 | A | G | -5E-05 | 0.000274 |
| rs55722786 | T | G | 0.028148 | 0.002735 | T | G | -5E-04 | 0.000294 |
| rs55724869 | C | G | 0.014174 | 0.002501 | C | G | 0.0005 | 0.000269 |
| rs55846720 | A | G | -0.014796 | 0.002479 | A | G | -3E-04 | 0.000266 |
| rs56094005 | G | A | -0.046542 | 0.006021 | G | A | 0.001 | 0.000647 |
| rs56188865 | C | T | 0.016247 | 0.002556 | C | T | 1E-05 | 0.000274 |
| rs58558667 | T | G | 0.023041 | 0.002553 | T | G | -5E-04 | 0.000274 |
| rs58579887 | C | T | 0.018348 | 0.002522 | C | T | 0.0004 | 0.000271 |
| rs59431480 | G | C | 0.085838 | 0.012738 | G | C | -0.001 | 0.001368 |
| rs59842359 | T | C | -0.022571 | 0.003073 | T | C | 0.0007 | 0.00033 |
| rs59916403 | T | G | -0.024299 | 0.002576 | T | G | 0.0001 | 0.000277 |
| rs6031847 | T | C | -0.021265 | 0.002777 | T | C | 0.0006 | 0.000298 |
| rs60644673 | T | G | -0.017542 | 0.003122 | T | G | 0.0006 | 0.000335 |
| rs6065295 | T | C | 0.015019 | 0.002491 | T | C | -4E-04 | 0.000268 |
| rs61552236 | ACT | A | -0.0161 | 0.002776 | ACT | A | 0.0006 | 0.000298 |
| rs61817641 | T | C | -0.018206 | 0.002782 | T | C | -8E-07 | 0.000299 |
| rs62053895 | A | G | -0.013849 | 0.002519 | A | G | 0.0004 | 0.00027 |
| rs62235071 | T | C | -0.040139 | 0.00522 | T | C | 0.0015 | 0.00056 |
| rs631695 | G | T | 0.015489 | 0.002494 | G | T | 0.0004 | 0.000268 |
| rs6490409 | G | A | -0.019425 | 0.003511 | G | A | 4E-05 | 0.000377 |
| rs6549406 | G | A | -0.018975 | 0.002788 | G | A | 1E-04 | 0.000299 |
| rs6567095 | A | G | 0.01941 | 0.002483 | A | G | -5E-04 | 0.000267 |
| rs667172 | A | G | 0.018923 | 0.002725 | A | G | -3E-05 | 0.000293 |
| rs6682695 | C | G | -0.023971 | 0.003321 | C | G | 0.0002 | 0.000357 |
| rs6693993 | C | G | 0.023539 | 0.00247 | C | G | -5E-05 | 0.000265 |
| rs6734238 | G | A | -0.016003 | 0.002507 | G | A | 9E-06 | 0.000269 |
| rs673751 | C | A | -0.015293 | 0.002638 | C | A | -6E-05 | 0.000283 |
| rs67694436 | T | C | 0.01512 | 0.002586 | T | C | -6E-05 | 0.000278 |
| rs6793835 | A | G | 0.016418 | 0.002803 | A | G | 2E-05 | 0.000301 |
| rs6794370 | C | A | -0.019261 | 0.003127 | C | A | -5E-04 | 0.000336 |
| rs6860245 | C | G | -0.021427 | 0.002858 | C | G | 9E-05 | 0.000307 |
| rs6871748 | C | T | 0.015224 | 0.002757 | C | T | 0.0002 | 0.000296 |
| rs6897617 | A | G | 0.018249 | 0.002716 | A | G | -9E-04 | 0.000292 |
| rs6912315 | T | C | 0.031344 | 0.005494 | T | C | 0.0003 | 0.00059 |
| rs6970593 | A | G | -0.025504 | 0.002472 | A | G | 4E-05 | 0.000266 |
| rs7031621 | A | G | -0.014376 | 0.002461 | A | G | 7E-05 | 0.000264 |
| rs71010816 | GT | G | -0.016915 | 0.002952 | GT | G | 0.0001 | 0.000317 |
| rs71587365 | ATT | A | -0.01932 | 0.002613 | ATT | A | -9E-05 | 0.000281 |
| rs72631343 | G | C | 0.021597 | 0.003685 | G | C | -5E-04 | 0.000396 |
| rs72694393 | T | G | 0.018287 | 0.002479 | T | G | -3E-04 | 0.000266 |
| rs72789541 | A | T | 0.018236 | 0.002703 | A | T | -6E-04 | 0.00029 |
| rs72793380 | A | C | 0.02248 | 0.004032 | A | C | -0.001 | 0.000433 |
| rs72818989 | C | T | 0.015766 | 0.002484 | C | T | 0.0002 | 0.000267 |
| rs72959041 | A | G | 0.051281 | 0.005716 | A | G | 0.0013 | 0.000614 |
| rs73038384 | T | C | -0.052059 | 0.007016 | T | C | 0.0009 | 0.000755 |
| rs73234873 | T | C | -0.014986 | 0.00271 | T | C | 0.0004 | 0.000291 |
| rs73263719 | A | G | 0.019352 | 0.00348 | A | G | 0.0003 | 0.000374 |
| rs7366884 | C | T | 0.018621 | 0.002762 | C | T | 3E-05 | 0.000297 |
| rs7402977 | A | G | -0.016271 | 0.002803 | A | G | -8E-05 | 0.000301 |
| rs74502455 | G | C | -0.02087 | 0.002464 | G | C | 0.0001 | 0.000265 |
| rs74538877 | C | G | 0.031123 | 0.005498 | C | G | -1E-03 | 0.000591 |
| rs74780677 | G | A | 0.093012 | 0.010096 | G | A | -1E-03 | 0.001084 |
| rs75070309 | A | C | 0.03197 | 0.003228 | A | C | 8E-05 | 0.000347 |
| rs7591567 | C | T | 0.015616 | 0.002704 | C | T | -5E-04 | 0.00029 |
| rs76006845 | G | GT | -0.016361 | 0.002874 | G | GT | 0.0002 | 0.000308 |
| rs76895963 | G | T | -0.071224 | 0.009449 | G | T | 0.0003 | 0.001016 |
| rs7731045 | C | T | -0.014222 | 0.002543 | C | T | -6E-05 | 0.000273 |
| rs77542162 | G | A | -0.19345 | 0.008216 | G | A | 0.0034 | 0.000882 |
| rs77849807 | G | A | 0.11375 | 0.00989 | G | A | -7E-04 | 0.001061 |
| rs78444263 | T | C | -0.03521 | 0.004796 | T | C | 0.0002 | 0.000515 |
| rs78961851 | C | T | -0.024799 | 0.004077 | C | T | -9E-04 | 0.000438 |
| rs79295634 | G | A | 0.03133 | 0.004938 | G | A | -3E-04 | 0.00053 |
| rs800545 | G | A | -0.016169 | 0.002833 | G | A | 0.0003 | 0.000304 |
| rs8041057 | T | C | -0.023221 | 0.002722 | T | C | 7E-05 | 0.000292 |
| rs8072215 | G | A | -0.033891 | 0.002914 | G | A | 5E-06 | 0.000313 |
| rs854796 | A | G | 0.015472 | 0.002654 | A | G | -2E-04 | 0.000285 |
| rs872926 | A | G | -0.035827 | 0.003037 | A | G | -6E-05 | 0.000326 |
| rs879620 | T | C | -0.014193 | 0.002541 | T | C | 0.0002 | 0.000273 |
| rs900400 | C | T | 0.022534 | 0.002516 | C | T | -6E-04 | 0.00027 |
| rs9262066 | T | G | 0.015398 | 0.002665 | T | G | -1E-04 | 0.000286 |
| rs9265945 | G | A | -0.023445 | 0.0025 | G | A | -2E-04 | 0.000268 |
| rs930340 | A | G | -0.018877 | 0.00316 | A | G | 0.0003 | 0.000339 |
| rs9391997 | G | A | -0.016206 | 0.002476 | G | A | 0.0002 | 0.000266 |
| rs9638180 | G | A | -0.024469 | 0.003091 | G | A | 0.0004 | 0.000332 |
| rs9917677 | C | T | -0.015506 | 0.00283 | C | T | 0.0007 | 0.000304 |
| rs992367 | G | A | 0.015386 | 0.002521 | G | A | 0.0002 | 0.000271 |
| rs9976946 | T | C | 0.038971 | 0.006444 | T | C | -4E-04 | 0.00069 |

SNP, single nucleotide polymorphism; VTE, venous thromboembolism; EA, effect allele; OA, other allele; SE, standard error

**Supplementary Table S4.** SNP serum albumin and SNP Stroke associations (per Effect Allele) of the instruments of serum albumin after data harmonization and outlier removal

| SNP | SNP-Serum Albumin | | | | Stroke | | | |
| --- | --- | --- | --- | --- | --- | --- | --- | --- |
|  | EA | OA | Beta | SE | EA | OA | Beta | SE |
| rs10004084 | C | T | 0.030092 | 0.003327 | C | T | -0.00042 | 0.00041 |
| rs10042492 | T | C | -0.01474 | 0.002478 | T | C | 0.000434 | 0.000306 |
| rs10143115 | A | G | -0.01466 | 0.002502 | A | G | 0.000735 | 0.000308 |
| rs10213692 | C | T | 0.019841 | 0.002898 | C | T | 0.000123 | 0.000357 |
| rs10236582 | C | T | -0.02217 | 0.002761 | C | T | 0.000153 | 0.00034 |
| rs1030098 | C | T | 0.020357 | 0.002543 | C | T | -0.00023 | 0.000313 |
| rs10419198 | T | C | -0.07391 | 0.002842 | T | C | 0.000358 | 0.000351 |
| rs10431419 | A | C | -0.01654 | 0.002956 | A | C | -0.00039 | 0.000365 |
| rs1043312 | G | T | 0.014211 | 0.002586 | G | T | -1E-05 | 0.000319 |
| rs10456852 | T | C | 0.026322 | 0.0037 | T | C | 0.0003 | 0.000456 |
| rs1079290 | A | T | 0.015474 | 0.002497 | A | T | -0.00035 | 0.000308 |
| rs10793127 | A | G | 0.024629 | 0.00427 | A | G | 0.00027 | 0.000526 |
| rs10863570 | C | T | 0.022492 | 0.002827 | C | T | -8.7E-05 | 0.000348 |
| rs10919543 | G | A | -0.01919 | 0.002669 | G | A | -0.00029 | 0.000329 |
| rs11012732 | G | A | -0.02334 | 0.002621 | G | A | 0.000901 | 0.000323 |
| rs11057273 | C | T | -0.02864 | 0.004247 | C | T | -0.00039 | 0.000523 |
| rs11074901 | A | G | 0.01473 | 0.002664 | A | G | -0.0003 | 0.000328 |
| rs11078597 | C | T | 0.065424 | 0.003154 | C | T | -0.00057 | 0.000389 |
| rs11088253 | T | C | 0.013768 | 0.002467 | T | C | 0.000332 | 0.000304 |
| rs1110659 | C | T | 0.02205 | 0.002891 | C | T | 4.26E-05 | 0.000356 |
| rs111443054 | TCACACA | T | -0.02849 | 0.003188 | TCACACA | T | 0.00043 | 0.000393 |
| rs111443054 | TCACACA | T | -0.02849 | 0.003188 | AGT | A | 0.000107 | 0.000548 |
| rs11208706 | G | T | 0.021997 | 0.002555 | G | T | 0.00033 | 0.000315 |
| rs11217135 | T | C | -0.02449 | 0.002478 | T | C | 5.49E-06 | 0.000305 |
| rs11264233 | A | G | 0.019323 | 0.002458 | A | G | 0.000376 | 0.000303 |
| rs113177823 | A | G | 0.0428 | 0.005493 | A | G | 0.000143 | 0.000677 |
| rs114949263 | C | T | 0.056977 | 0.003919 | C | T | -0.00116 | 0.000483 |
| rs11589479 | A | G | 0.045527 | 0.003316 | A | G | 0.00021 | 0.000409 |
| rs11609805 | A | G | -0.02541 | 0.002866 | A | G | 0.000298 | 0.000353 |
| rs11656541 | C | G | 0.026835 | 0.00259 | C | G | 0.000175 | 0.000319 |
| rs117127664 | T | C | 0.037526 | 0.006224 | T | C | 0.000469 | 0.000767 |
| rs11736842 | T | A | 0.015911 | 0.002556 | T | A | -0.00034 | 0.000315 |
| rs117820542 | G | C | -0.04114 | 0.007446 | G | C | 0.001072 | 0.000919 |
| rs11895352 | T | C | -0.01366 | 0.002467 | T | C | -0.00016 | 0.000304 |
| rs11928797 | A | C | 0.027873 | 0.003829 | A | C | -0.00072 | 0.000472 |
| rs12215904 | T | C | -0.01996 | 0.003201 | T | C | -0.00088 | 0.000395 |
| rs1229492 | C | T | -0.01708 | 0.0028 | C | T | 0.000107 | 0.000345 |
| rs1229984 | C | T | 0.059701 | 0.008334 | C | T | 0.002264 | 0.001027 |
| rs12377600 | G | A | -0.01816 | 0.002584 | G | A | 0.000297 | 0.000319 |
| rs12563096 | A | G | 0.01427 | 0.002614 | A | G | -0.00017 | 0.000322 |
| rs1260326 | C | T | -0.059 | 0.002516 | C | T | -0.00036 | 0.00031 |
| rs12710562 | G | A | 0.026315 | 0.003187 | G | A | -7.3E-05 | 0.000393 |
| rs12815728 | C | T | 0.018075 | 0.002477 | C | T | -0.00025 | 0.000305 |
| rs12881869 | T | C | -0.04948 | 0.004787 | T | C | -1.5E-05 | 0.00059 |
| rs12924886 | T | A | 0.029495 | 0.003148 | T | A | -0.0003 | 0.000388 |
| rs13107325 | T | C | -0.05589 | 0.004682 | T | C | -0.00122 | 0.000577 |
| rs13108218 | G | A | -0.04425 | 0.002553 | G | A | 0.000221 | 0.000315 |
| rs13111128 | A | G | -0.01861 | 0.002714 | A | G | 0.000117 | 0.000334 |
| rs1331309 | G | T | 0.020886 | 0.002817 | G | T | 0.000598 | 0.000347 |
| rs13389219 | T | C | -0.02936 | 0.002518 | T | C | 0.000166 | 0.000311 |
| rs138833981 | G | C | -0.08581 | 0.009893 | G | C | -0.00128 | 0.001221 |
| rs139278099 | T | C | 0.062656 | 0.00619 | T | C | 0.000607 | 0.000762 |
| rs139974673 | C | T | 0.15127 | 0.00788 | C | T | -0.0009 | 0.000974 |
| rs147651823 | A | C | -0.03493 | 0.005891 | A | C | 6.78E-05 | 0.000726 |
| rs149092986 | C | T | -0.0549 | 0.008357 | C | T | -0.00095 | 0.00103 |
| rs1497406 | G | A | 0.025056 | 0.002491 | G | A | -8.5E-06 | 0.000307 |
| rs1500187 | G | A | -0.02369 | 0.002472 | G | A | 9.99E-05 | 0.000305 |
| rs150783681 | C | G | -0.11931 | 0.008754 | C | G | -0.00017 | 0.001081 |
| rs1532085 | G | A | 0.014174 | 0.002528 | G | A | -8.6E-05 | 0.000311 |
| rs157936 | G | T | -0.01904 | 0.002696 | G | T | 7.4E-05 | 0.000332 |
| rs1593357 | T | C | -0.02036 | 0.002864 | T | C | 0.00029 | 0.000353 |
| rs16950612 | G | A | -0.02544 | 0.003943 | G | A | -0.0005 | 0.000486 |
| rs17023530 | A | T | -0.03838 | 0.005752 | A | T | -0.00033 | 0.000709 |
| rs17580 | A | T | 0.094752 | 0.005709 | A | T | -0.00024 | 0.000705 |
| rs1782455 | A | G | 0.019719 | 0.00334 | A | G | -0.00064 | 0.000412 |
| rs1791936 | A | G | 0.021346 | 0.002518 | A | G | 0.000103 | 0.00031 |
| rs1801282 | G | C | -0.05066 | 0.003774 | G | C | 0.000112 | 0.000465 |
| rs1880241 | G | A | 0.015573 | 0.002469 | G | A | -0.00047 | 0.000304 |
| rs1886839 | G | A | 0.014096 | 0.002513 | G | A | 5.91E-05 | 0.00031 |
| rs198426 | T | C | -0.01691 | 0.002607 | T | C | -3.5E-07 | 0.000321 |
| rs1986133 | T | C | 0.016108 | 0.002656 | T | C | 5.86E-06 | 0.000327 |
| rs1998528 | A | G | 0.015217 | 0.002586 | A | G | -0.0004 | 0.000319 |
| rs201931078 | GTGT | G | -0.01684 | 0.002705 | GTGT | G | 0.000382 | 0.000333 |
| rs2060658 | C | T | 0.016588 | 0.002478 | C | T | -0.00042 | 0.000305 |
| rs2072442 | G | C | 0.014529 | 0.002492 | G | C | -0.0002 | 0.000307 |
| rs2115868 | A | T | -0.02438 | 0.003343 | A | T | 0.000666 | 0.000412 |
| rs2169387 | G | A | 0.057547 | 0.004108 | G | A | 4.8E-05 | 0.000506 |
| rs2200061 | A | G | -0.0165 | 0.003013 | A | G | 1.07E-05 | 0.000371 |
| rs2227827 | T | C | -0.05124 | 0.005762 | T | C | -0.00089 | 0.000709 |
| rs2267867 | G | A | -0.01609 | 0.002896 | G | A | -6.9E-05 | 0.000357 |
| rs2303695 | T | C | -0.02567 | 0.002582 | T | C | 0.000507 | 0.000319 |
| rs2304130 | G | A | 0.02707 | 0.004395 | G | A | 9.77E-05 | 0.000542 |
| rs234043 | C | T | 0.016912 | 0.002733 | C | T | -2.3E-05 | 0.000337 |
| rs2638315 | C | G | 0.035351 | 0.00318 | C | G | -0.00085 | 0.000392 |
| rs2702571 | A | T | 0.016766 | 0.00258 | A | T | -0.00031 | 0.000318 |
| rs2710804 | C | T | -0.01647 | 0.002545 | C | T | 0.00043 | 0.000314 |
| rs2785172 | A | G | -0.01916 | 0.002527 | A | G | 0.000201 | 0.000312 |
| rs2820446 | G | C | -0.02399 | 0.002685 | G | C | -0.00021 | 0.000331 |
| rs28601761 | G | C | -0.01996 | 0.002523 | G | C | 0.000104 | 0.000311 |
| rs28687959 | T | C | 0.016421 | 0.002478 | T | C | -0.0002 | 0.000306 |
| rs28688002 | A | T | 0.027203 | 0.002951 | A | T | 6.65E-05 | 0.000364 |
| rs2869876 | A | C | 0.017918 | 0.003186 | A | C | -0.00045 | 0.000393 |
| rs28929474 | T | C | 0.26514 | 0.008734 | T | C | -0.0001 | 0.001076 |
| rs2972145 | C | T | 0.021428 | 0.002573 | C | T | 0.000305 | 0.000317 |
| rs3099371 | T | C | 0.015872 | 0.002512 | T | C | -4.2E-06 | 0.00031 |
| rs3184504 | C | T | 0.021388 | 0.002463 | C | T | -0.00043 | 0.000303 |
| rs34562254 | A | G | -0.03754 | 0.004139 | A | G | 0.000785 | 0.00051 |
| rs34596921 | TA | T | -0.01865 | 0.002524 | TA | T | 0.00016 | 0.000311 |
| rs34783842 | TA | T | 0.040771 | 0.002467 | TA | T | -0.00061 | 0.000304 |
| rs34931250 | T | C | 0.03341 | 0.005134 | T | C | -0.00061 | 0.000633 |
| rs35123414 | C | T | 0.021697 | 0.003622 | C | T | 0.000354 | 0.000447 |
| rs35676551 | A | C | -0.0379 | 0.00554 | A | C | -0.00088 | 0.000683 |
| rs36090025 | C | A | 0.017388 | 0.002687 | C | A | 1.04E-05 | 0.000331 |
| rs3740688 | T | G | 0.024193 | 0.002475 | T | G | -0.00033 | 0.000305 |
| rs3768321 | T | G | -0.02842 | 0.003099 | T | G | -0.00069 | 0.000382 |
| rs378740 | A | C | 0.01565 | 0.002779 | A | C | -0.00023 | 0.000342 |
| rs3810484 | G | A | 0.014777 | 0.002477 | G | A | -0.00034 | 0.000305 |
| rs390801 | C | T | 0.021268 | 0.002802 | C | T | 0.00017 | 0.000345 |
| rs4327724 | T | C | 0.031296 | 0.004594 | T | C | -0.00038 | 0.000567 |
| rs4410790 | C | T | 0.015033 | 0.002557 | C | T | 0.000424 | 0.000315 |
| rs4458838 | A | G | -0.01566 | 0.002581 | A | G | 0.000876 | 0.000318 |
| rs4499445 | T | G | -0.02174 | 0.002604 | T | G | 0.000374 | 0.000321 |
| rs45439091 | T | G | 0.031713 | 0.004747 | T | G | -0.00019 | 0.000585 |
| rs45512696 | T | C | 0.080056 | 0.003232 | T | C | -0.00041 | 0.000399 |
| rs459193 | G | A | 0.022894 | 0.002978 | G | A | -0.00021 | 0.000367 |
| rs4711399 | T | C | 0.021968 | 0.002966 | T | C | -0.00017 | 0.000365 |
| rs473919 | G | C | -0.01888 | 0.003124 | G | C | 0.000346 | 0.000385 |
| rs4782568 | G | C | -0.01843 | 0.002489 | G | C | -6.6E-05 | 0.000307 |
| rs4790875 | T | C | -0.02071 | 0.002513 | T | C | 0.000134 | 0.00031 |
| rs4796307 | A | T | -0.02815 | 0.003001 | A | T | 0.000538 | 0.00037 |
| rs4804413 | T | C | 0.017594 | 0.002488 | T | C | 0.00022 | 0.000307 |
| rs4805129 | C | T | -0.02111 | 0.002546 | C | T | -1.3E-05 | 0.000314 |
| rs4805881 | C | A | -0.02704 | 0.002609 | C | A | 0.000748 | 0.000322 |
| rs4833945 | C | T | 0.021705 | 0.003854 | C | T | -0.00016 | 0.000476 |
| rs4946811 | C | A | 0.015897 | 0.002567 | C | A | 0.000158 | 0.000316 |
| rs4970834 | T | C | -0.02539 | 0.003169 | T | C | -0.00085 | 0.00039 |
| rs55696240 | A | G | 0.017035 | 0.002551 | A | G | 0.000275 | 0.000314 |
| rs55722786 | T | G | 0.028148 | 0.002735 | T | G | -0.00032 | 0.000337 |
| rs55724869 | C | G | 0.014174 | 0.002501 | C | G | 0.000382 | 0.000308 |
| rs55846720 | A | G | -0.0148 | 0.002479 | A | G | -0.00044 | 0.000306 |
| rs56094005 | G | A | -0.04654 | 0.006021 | G | A | -0.00028 | 0.000742 |
| rs56188865 | C | T | 0.016247 | 0.002556 | C | T | 8.18E-05 | 0.000315 |
| rs58558667 | T | G | 0.023041 | 0.002553 | T | G | 4E-05 | 0.000315 |
| rs58579887 | C | T | 0.018348 | 0.002522 | C | T | -0.00018 | 0.000311 |
| rs59431480 | G | C | 0.085838 | 0.012738 | G | C | 0.000404 | 0.00157 |
| rs59842359 | T | C | -0.02257 | 0.003073 | T | C | -0.00034 | 0.000379 |
| rs59916403 | T | G | -0.0243 | 0.002576 | T | G | 0.000255 | 0.000317 |
| rs6031847 | T | C | -0.02127 | 0.002777 | T | C | -0.00029 | 0.000342 |
| rs60644673 | T | G | -0.01754 | 0.003122 | T | G | -3E-05 | 0.000385 |
| rs6065295 | T | C | 0.015019 | 0.002491 | T | C | -0.0005 | 0.000307 |
| rs61552236 | ACT | A | -0.0161 | 0.002776 | ACT | A | 0.000209 | 0.000342 |
| rs61817641 | T | C | -0.01821 | 0.002782 | T | C | 0.000379 | 0.000343 |
| rs61983272 | C | G | -0.03286 | 0.002844 | C | G | 0.000258 | 0.000351 |
| rs62053895 | A | G | -0.01385 | 0.002519 | A | G | -0.00012 | 0.00031 |
| rs62235071 | T | C | -0.04014 | 0.00522 | T | C | 0.000135 | 0.000643 |
| rs631695 | G | T | 0.015489 | 0.002494 | G | T | 0.000319 | 0.000307 |
| rs6490409 | G | A | -0.01943 | 0.003511 | G | A | 0.000302 | 0.000432 |
| rs6549406 | G | A | -0.01898 | 0.002788 | G | A | -0.00012 | 0.000344 |
| rs6567095 | A | G | 0.01941 | 0.002483 | A | G | -0.0001 | 0.000306 |
| rs667172 | A | G | 0.018923 | 0.002725 | A | G | 0.000186 | 0.000336 |
| rs6682695 | C | G | -0.02397 | 0.003321 | C | G | 0.000326 | 0.000409 |
| rs6693993 | C | G | 0.023539 | 0.00247 | C | G | -0.00013 | 0.000304 |
| rs6734238 | G | A | -0.016 | 0.002507 | G | A | 3.08E-05 | 0.000309 |
| rs673751 | C | A | -0.01529 | 0.002638 | C | A | 5.63E-05 | 0.000325 |
| rs67694436 | T | C | 0.01512 | 0.002586 | T | C | 0.000254 | 0.000319 |
| rs6793835 | A | G | 0.016418 | 0.002803 | A | G | -0.00074 | 0.000345 |
| rs6794370 | C | A | -0.01926 | 0.003127 | C | A | -9.2E-06 | 0.000385 |
| rs6860245 | C | G | -0.02143 | 0.002858 | C | G | -0.00058 | 0.000352 |
| rs6871748 | C | T | 0.015224 | 0.002757 | C | T | 0.000388 | 0.00034 |
| rs6897617 | A | G | 0.018249 | 0.002716 | A | G | -0.00022 | 0.000335 |
| rs6912315 | T | C | 0.031344 | 0.005494 | T | C | 0.00042 | 0.000677 |
| rs6970593 | A | G | -0.0255 | 0.002472 | A | G | 7.24E-05 | 0.000305 |
| rs7031621 | A | G | -0.01438 | 0.002461 | A | G | 0.000348 | 0.000303 |
| rs71010816 | GT | G | -0.01692 | 0.002952 | GT | G | -7.6E-05 | 0.000364 |
| rs71587365 | ATT | A | -0.01932 | 0.002613 | ATT | A | -1.1E-05 | 0.000322 |
| rs72631343 | G | C | 0.021597 | 0.003685 | G | C | -2.8E-05 | 0.000455 |
| rs72694393 | T | G | 0.018287 | 0.002479 | T | G | -0.00027 | 0.000306 |
| rs72789541 | A | T | 0.018236 | 0.002703 | A | T | 9.42E-06 | 0.000333 |
| rs72793380 | A | C | 0.02248 | 0.004032 | A | C | 0.000597 | 0.000497 |
| rs72818989 | C | T | 0.015766 | 0.002484 | C | T | -4E-05 | 0.000306 |
| rs72959041 | A | G | 0.051281 | 0.005716 | A | G | -0.00105 | 0.000705 |
| rs73038384 | T | C | -0.05206 | 0.007016 | T | C | 0.000577 | 0.000867 |
| rs73234873 | T | C | -0.01499 | 0.00271 | T | C | 3.35E-05 | 0.000334 |
| rs73263719 | A | G | 0.019352 | 0.00348 | A | G | -0.0002 | 0.000429 |
| rs7366884 | C | T | 0.018621 | 0.002762 | C | T | 0.000126 | 0.000341 |
| rs7402977 | A | G | -0.01627 | 0.002803 | A | G | -3E-05 | 0.000345 |
| rs74502455 | G | C | -0.02087 | 0.002464 | G | C | 0.000544 | 0.000304 |
| rs74538877 | C | G | 0.031123 | 0.005498 | C | G | -0.0003 | 0.000678 |
| rs74780677 | G | A | 0.093012 | 0.010096 | G | A | 2.65E-05 | 0.001244 |
| rs75070309 | A | C | 0.03197 | 0.003228 | A | C | -8.3E-07 | 0.000398 |
| rs7591567 | C | T | 0.015616 | 0.002704 | C | T | -0.00039 | 0.000333 |
| rs76006845 | G | GT | -0.01636 | 0.002874 | G | GT | -0.00039 | 0.000354 |
| rs76895963 | G | T | -0.07122 | 0.009449 | G | T | -0.00091 | 0.001166 |
| rs7731045 | C | T | -0.01422 | 0.002543 | C | T | -0.00017 | 0.000314 |
| rs77542162 | G | A | -0.19345 | 0.008216 | G | A | 3.17E-05 | 0.001013 |
| rs77849807 | G | A | 0.11375 | 0.00989 | G | A | -0.00041 | 0.001218 |
| rs78444263 | T | C | -0.03521 | 0.004796 | T | C | 1.79E-05 | 0.000591 |
| rs78961851 | C | T | -0.0248 | 0.004077 | C | T | 0.000498 | 0.000502 |
| rs79295634 | G | A | 0.03133 | 0.004938 | G | A | -0.001 | 0.000609 |
| rs800545 | G | A | -0.01617 | 0.002833 | G | A | 0.000567 | 0.000349 |
| rs8041057 | T | C | -0.02322 | 0.002722 | T | C | 0.000161 | 0.000335 |
| rs8072215 | G | A | -0.03389 | 0.002914 | G | A | 0.000117 | 0.000359 |
| rs854796 | A | G | 0.015472 | 0.002654 | A | G | 0.000468 | 0.000327 |
| rs872926 | A | G | -0.03583 | 0.003037 | A | G | -0.00019 | 0.000374 |
| rs879620 | T | C | -0.01419 | 0.002541 | T | C | 0.000222 | 0.000313 |
| rs900400 | C | T | 0.022534 | 0.002516 | C | T | -0.00055 | 0.00031 |
| rs9262066 | T | G | 0.015398 | 0.002665 | T | G | 0.000261 | 0.000328 |
| rs9265945 | G | A | -0.02345 | 0.0025 | G | A | 0.000133 | 0.000308 |
| rs930340 | A | G | -0.01888 | 0.00316 | A | G | -0.0005 | 0.00039 |
| rs9391997 | G | A | -0.01621 | 0.002476 | G | A | -0.00016 | 0.000305 |
| rs9638180 | G | A | -0.02447 | 0.003091 | G | A | 0.000526 | 0.000381 |
| rs9917677 | C | T | -0.01551 | 0.00283 | C | T | 0.000203 | 0.000349 |
| rs992367 | G | A | 0.015386 | 0.002521 | G | A | 0.000102 | 0.000311 |
| rs9976946 | T | C | 0.038971 | 0.006444 | T | C | 0.001262 | 0.000792 |

SNP, single nucleotide polymorphism; EA, effect allele; OA, other allele; SE, standard error

**Supplementary Table S5.** SNP serum albumin and SNP AF associations (per Effect Allele) of the instruments of serum albumin after data harmonization and outlier removal

| SNP | SNP-Serum Albumin | | | | SNP-AF | | | |
| --- | --- | --- | --- | --- | --- | --- | --- | --- |
|  | EA | OA | Beta | SE | EA | OA | Beta | SE |
| rs10004084 | C | T | 0.030092 | 0.003327 | C | T | -0.0056 | 0.0089 |
| rs10042492 | T | C | -0.01474 | 0.002478 | T | C | -0.0007 | 0.0067 |
| rs10143115 | A | G | -0.01466 | 0.002502 | A | G | -0.0124 | 0.0094 |
| rs10213692 | C | T | 0.019841 | 0.002898 | C | T | 0.0084 | 0.008 |
| rs10236582 | C | T | -0.02217 | 0.002761 | C | T | -0.008 | 0.0074 |
| rs1030098 | C | T | 0.020357 | 0.002543 | C | T | 0.0025 | 0.0068 |
| rs10419198 | T | C | -0.07391 | 0.002842 | T | C | 0.0087 | 0.0079 |
| rs10431419 | A | C | -0.01654 | 0.002956 | A | C | 0.0146 | 0.008 |
| rs1043312 | G | T | 0.014211 | 0.002586 | G | T | 0.0087 | 0.007 |
| rs10456852 | T | C | 0.026322 | 0.0037 | T | C | -0.0094 | 0.0103 |
| rs1079290 | A | T | 0.015474 | 0.002497 | A | T | -0.0028 | 0.0067 |
| rs10793127 | A | G | 0.024629 | 0.00427 | A | G | -0.0004 | 0.0146 |
| rs10863570 | C | T | 0.022492 | 0.002827 | C | T | -0.0056 | 0.0077 |
| rs10919543 | G | A | -0.01919 | 0.002669 | G | A | -0.0004 | 0.0075 |
| rs11012732 | G | A | -0.02334 | 0.002621 | G | A | 0.0082 | 0.0071 |
| rs11057273 | C | T | -0.02864 | 0.004247 | C | T | -0.0145 | 0.0112 |
| rs11074901 | A | G | 0.01473 | 0.002664 | A | G | -0.0061 | 0.0072 |
| rs11078597 | C | T | 0.065424 | 0.003154 | C | T | -0.0135 | 0.0086 |
| rs11088253 | T | C | 0.013768 | 0.002467 | T | C | 0.0004 | 0.0067 |
| rs1110659 | C | T | 0.02205 | 0.002891 | C | T | 0.0005 | 0.0081 |
| rs11208706 | G | T | 0.021997 | 0.002555 | G | T | 0.0172 | 0.0172 |
| rs11217135 | T | C | -0.02449 | 0.002478 | T | C | -0.0101 | 0.0167 |
| rs11264233 | A | G | 0.019323 | 0.002458 | A | G | -0.0069 | 0.0067 |
| rs113177823 | A | G | 0.0428 | 0.005493 | A | G | 0.016 | 0.0169 |
| rs114949263 | C | T | 0.056977 | 0.003919 | C | T | -0.0069 | 0.0108 |
| rs11589479 | A | G | 0.045527 | 0.003316 | A | G | -0.0217 | 0.0094 |
| rs11609805 | A | G | -0.02541 | 0.002866 | A | G | -0.0044 | 0.0084 |
| rs11656541 | C | G | 0.026835 | 0.00259 | C | G | -0.001 | 0.0071 |
| rs117127664 | T | C | 0.037526 | 0.006224 | T | C | 0.0187 | 0.017 |
| rs11736842 | T | A | 0.015911 | 0.002556 | T | A | -0.0023 | 0.0069 |
| rs117820542 | G | C | -0.04114 | 0.007446 | G | C | -0.0463 | 0.0214 |
| rs11895352 | T | C | -0.01366 | 0.002467 | T | C | -0.0023 | 0.0066 |
| rs11928797 | A | C | 0.027873 | 0.003829 | A | C | 0.0144 | 0.0109 |
| rs12215904 | T | C | -0.01996 | 0.003201 | T | C | -0.0043 | 0.0085 |
| rs1229492 | C | T | -0.01708 | 0.0028 | C | T | 0.0007 | 0.0074 |
| rs1229984 | C | T | 0.059701 | 0.008334 | C | T | 0.0389 | 0.0227 |
| rs12377600 | G | A | -0.01816 | 0.002584 | G | A | -0.0084 | 0.0069 |
| rs12563096 | A | G | 0.01427 | 0.002614 | A | G | 0.0066 | 0.0225 |
| rs1260326 | C | T | -0.059 | 0.002516 | C | T | 0.0017 | 0.0068 |
| rs12710562 | G | A | 0.026315 | 0.003187 | G | A | 0.0022 | 0.0089 |
| rs12815728 | C | T | 0.018075 | 0.002477 | C | T | -0.0031 | 0.0067 |
| rs12881869 | T | C | -0.04948 | 0.004787 | T | C | -0.0102 | 0.0134 |
| rs12924886 | T | A | 0.029495 | 0.003148 | T | A | -0.011 | 0.0085 |
| rs13107325 | T | C | -0.05589 | 0.004682 | T | C | 0.013 | 0.0142 |
| rs13108218 | G | A | -0.04425 | 0.002553 | G | A | 0.0001 | 0.007 |
| rs13111128 | A | G | -0.01861 | 0.002714 | A | G | 0.009 | 0.011 |
| rs1331309 | G | T | 0.020886 | 0.002817 | G | T | 0.0021 | 0.0102 |
| rs13389219 | T | C | -0.02936 | 0.002518 | T | C | 0.0024 | 0.0067 |
| rs138833981 | G | C | -0.08581 | 0.009893 | G | C | 0.0152 | 0.0323 |
| rs139278099 | T | C | 0.062656 | 0.00619 | T | C | 0.0047 | 0.0195 |
| rs139974673 | C | T | 0.15127 | 0.00788 | C | T | -0.0131 | 0.0213 |
| rs147651823 | A | C | -0.03493 | 0.005891 | A | C | 0.0306 | 0.0152 |
| rs149092986 | C | T | -0.0549 | 0.008357 | C | T | -0.0185 | 0.0255 |
| rs1497406 | G | A | 0.025056 | 0.002491 | G | A | -0.0009 | 0.0067 |
| rs1500187 | G | A | -0.02369 | 0.002472 | G | A | -0.0022 | 0.0067 |
| rs150783681 | C | G | -0.11931 | 0.008754 | C | G | 0.065 | 0.026 |
| rs1532085 | G | A | 0.014174 | 0.002528 | G | A | -0.0158 | 0.0068 |
| rs157936 | G | T | -0.01904 | 0.002696 | G | T | 0.0147 | 0.0073 |
| rs1593357 | T | C | -0.02036 | 0.002864 | T | C | -0.0143 | 0.0077 |
| rs16950612 | G | A | -0.02544 | 0.003943 | G | A | -0.0005 | 0.0118 |
| rs17023530 | A | T | -0.03838 | 0.005752 | A | T | -0.0061 | 0.0152 |
| rs17580 | A | T | 0.094752 | 0.005709 | A | T | -0.0099 | 0.0175 |
| rs1782455 | A | G | 0.019719 | 0.00334 | A | G | -0.0034 | 0.0088 |
| rs1791936 | A | G | 0.021346 | 0.002518 | A | G | 0.0036 | 0.0069 |
| rs1801282 | G | C | -0.05066 | 0.003774 | G | C | 0.0327 | 0.0101 |
| rs1880241 | G | A | 0.015573 | 0.002469 | G | A | -0.0142 | 0.0067 |
| rs1886839 | G | A | 0.014096 | 0.002513 | G | A | -0.0045 | 0.0068 |
| rs198426 | T | C | -0.01691 | 0.002607 | T | C | 0.0109 | 0.0071 |
| rs1986133 | T | C | 0.016108 | 0.002656 | T | C | 0.0029 | 0.0072 |
| rs1998528 | A | G | 0.015217 | 0.002586 | A | G | -0.009 | 0.007 |
| rs2060658 | C | T | 0.016588 | 0.002478 | C | T | 0.0113 | 0.0067 |
| rs2072442 | G | C | 0.014529 | 0.002492 | G | C | 0.001 | 0.0067 |
| rs2115868 | A | T | -0.02438 | 0.003343 | A | T | 0.0146 | 0.0091 |
| rs2169387 | G | A | 0.057547 | 0.004108 | G | A | 0.0215 | 0.0114 |
| rs2200061 | A | G | -0.0165 | 0.003013 | A | G | 0.0144 | 0.008 |
| rs2227827 | T | C | -0.05124 | 0.005762 | T | C | 0.0513 | 0.0171 |
| rs2267867 | G | A | -0.01609 | 0.002896 | G | A | -0.0015 | 0.0078 |
| rs2303695 | T | C | -0.02567 | 0.002582 | T | C | -0.0132 | 0.007 |
| rs2304130 | G | A | 0.02707 | 0.004395 | G | A | -0.0045 | 0.0121 |
| rs234043 | C | T | 0.016912 | 0.002733 | C | T | 0.0108 | 0.0074 |
| rs2638315 | C | G | 0.035351 | 0.00318 | C | G | -0.0014 | 0.0089 |
| rs2702571 | A | T | 0.016766 | 0.00258 | A | T | -0.0067 | 0.007 |
| rs2710804 | C | T | -0.01647 | 0.002545 | C | T | -0.0074 | 0.0069 |
| rs2785172 | A | G | -0.01916 | 0.002527 | A | G | -0.0015 | 0.0068 |
| rs2820446 | G | C | -0.02399 | 0.002685 | G | C | 0.0011 | 0.0072 |
| rs28601761 | G | C | -0.01996 | 0.002523 | G | C | -0.0025 | 0.0067 |
| rs28688002 | A | T | 0.027203 | 0.002951 | A | T | -0.0051 | 0.0078 |
| rs2869876 | A | C | 0.017918 | 0.003186 | A | C | -0.0061 | 0.0092 |
| rs28929474 | T | C | 0.26514 | 0.008734 | T | C | 0.0221 | 0.0275 |
| rs2972145 | C | T | 0.021428 | 0.002573 | C | T | -0.0124 | 0.007 |
| rs3099371 | T | C | 0.015872 | 0.002512 | T | C | 0.0128 | 0.0067 |
| rs3184504 | C | T | 0.021388 | 0.002463 | C | T | -0.0038 | 0.0067 |
| rs34562254 | A | G | -0.03754 | 0.004139 | A | G | -0.0024 | 0.011 |
| rs34931250 | T | C | 0.03341 | 0.005134 | T | C | -0.0032 | 0.0145 |
| rs35123414 | C | T | 0.021697 | 0.003622 | C | T | -0.0131 | 0.0108 |
| rs35676551 | A | C | -0.0379 | 0.00554 | A | C | -0.0224 | 0.0153 |
| rs36090025 | C | A | 0.017388 | 0.002687 | C | A | -0.0124 | 0.0073 |
| rs3740688 | T | G | 0.024193 | 0.002475 | T | G | 0.0138 | 0.0067 |
| rs3768321 | T | G | -0.02842 | 0.003099 | T | G | 0.0122 | 0.0085 |
| rs378740 | A | C | 0.01565 | 0.002779 | A | C | -0.0241 | 0.0074 |
| rs3810484 | G | A | 0.014777 | 0.002477 | G | A | 0.0004 | 0.0071 |
| rs390801 | C | T | 0.021268 | 0.002802 | C | T | -0.0151 | 0.0076 |
| rs4327724 | T | C | 0.031296 | 0.004594 | T | C | 0.0192 | 0.0121 |
| rs4410790 | C | T | 0.015033 | 0.002557 | C | T | 0.0095 | 0.0069 |
| rs4458838 | A | G | -0.01566 | 0.002581 | A | G | 0.0143 | 0.0071 |
| rs4499445 | T | G | -0.02174 | 0.002604 | T | G | 0.0014 | 0.0097 |
| rs45439091 | T | G | 0.031713 | 0.004747 | T | G | 0.0126 | 0.0134 |
| rs45512696 | T | C | 0.080056 | 0.003232 | T | C | -0.014 | 0.0091 |
| rs459193 | G | A | 0.022894 | 0.002978 | G | A | -0.0004 | 0.0075 |
| rs4711399 | T | C | 0.021968 | 0.002966 | T | C | 0.0012 | 0.008 |
| rs473919 | G | C | -0.01888 | 0.003124 | G | C | -0.0044 | 0.0085 |
| rs4782568 | G | C | -0.01843 | 0.002489 | G | C | 0.0158 | 0.007 |
| rs4790875 | T | C | -0.02071 | 0.002513 | T | C | 0.0059 | 0.0068 |
| rs4796307 | A | T | -0.02815 | 0.003001 | A | T | -0.0314 | 0.0124 |
| rs4804413 | T | C | 0.017594 | 0.002488 | T | C | -0.0118 | 0.0068 |
| rs4805129 | C | T | -0.02111 | 0.002546 | C | T | 0.0009 | 0.007 |
| rs4805881 | C | A | -0.02704 | 0.002609 | C | A | 0.01 | 0.0071 |
| rs4833945 | C | T | 0.021705 | 0.003854 | C | T | -0.0011 | 0.01 |
| rs4946811 | C | A | 0.015897 | 0.002567 | C | A | -0.0015 | 0.0071 |
| rs4970834 | T | C | -0.02539 | 0.003169 | T | C | -0.0171 | 0.0085 |
| rs55696240 | A | G | 0.017035 | 0.002551 | A | G | 0.0003 | 0.0069 |
| rs55722786 | T | G | 0.028148 | 0.002735 | T | G | -0.0067 | 0.0075 |
| rs55724869 | C | G | 0.014174 | 0.002501 | C | G | -0.0033 | 0.0068 |
| rs55846720 | A | G | -0.0148 | 0.002479 | A | G | -0.0184 | 0.0067 |
| rs56094005 | G | A | -0.04654 | 0.006021 | G | A | 0.0044 | 0.0179 |
| rs56188865 | C | T | 0.016247 | 0.002556 | C | T | 0.0077 | 0.0069 |
| rs58558667 | T | G | 0.023041 | 0.002553 | T | G | -0.0037 | 0.0069 |
| rs58579887 | C | T | 0.018348 | 0.002522 | C | T | -0.015 | 0.0069 |
| rs59431480 | G | C | 0.085838 | 0.012738 | G | C | -0.014 | 0.0342 |
| rs59842359 | T | C | -0.02257 | 0.003073 | T | C | -0.0187 | 0.0083 |
| rs59916403 | T | G | -0.0243 | 0.002576 | T | G | 0.0032 | 0.007 |
| rs6031847 | T | C | -0.02127 | 0.002777 | T | C | -0.006 | 0.0076 |
| rs60644673 | T | G | -0.01754 | 0.003122 | T | G | 0.0118 | 0.0086 |
| rs6065295 | T | C | 0.015019 | 0.002491 | T | C | -0.0087 | 0.0104 |
| rs61552236 | ACT | A | -0.0161 | 0.002776 | ACT | A | -0.0026 | 0.0184 |
| rs61817641 | T | C | -0.01821 | 0.002782 | T | C | 0.0101 | 0.0107 |
| rs61983272 | C | G | -0.03286 | 0.002844 | C | G | -0.0059 | 0.0085 |
| rs62053895 | A | G | -0.01385 | 0.002519 | A | G | 0.0063 | 0.0068 |
| rs62235071 | T | C | -0.04014 | 0.00522 | T | C | 0.0446 | 0.0149 |
| rs631695 | G | T | 0.015489 | 0.002494 | G | T | -0.0067 | 0.0068 |
| rs6490409 | G | A | -0.01943 | 0.003511 | G | A | 0.0014 | 0.0091 |
| rs6549406 | G | A | -0.01898 | 0.002788 | G | A | -0.025 | 0.0075 |
| rs6567095 | A | G | 0.01941 | 0.002483 | A | G | -0.0006 | 0.0068 |
| rs667172 | A | G | 0.018923 | 0.002725 | A | G | -0.0006 | 0.0073 |
| rs6682695 | C | G | -0.02397 | 0.003321 | C | G | 0.0015 | 0.0091 |
| rs6693993 | C | G | 0.023539 | 0.00247 | C | G | -0.0088 | 0.0067 |
| rs6734238 | G | A | -0.016 | 0.002507 | G | A | 0.0008 | 0.0068 |
| rs673751 | C | A | -0.01529 | 0.002638 | C | A | -0.0136 | 0.0071 |
| rs67694436 | T | C | 0.01512 | 0.002586 | T | C | -0.0065 | 0.0071 |
| rs6793835 | A | G | 0.016418 | 0.002803 | A | G | -0.0148 | 0.0076 |
| rs6794370 | C | A | -0.01926 | 0.003127 | C | A | 0.0253 | 0.0111 |
| rs6860245 | C | G | -0.02143 | 0.002858 | C | G | -0.019 | 0.0079 |
| rs6871748 | C | T | 0.015224 | 0.002757 | C | T | 0.017 | 0.0074 |
| rs6912315 | T | C | 0.031344 | 0.005494 | T | C | -0.0265 | 0.015 |
| rs6970593 | A | G | -0.0255 | 0.002472 | A | G | -0.0116 | 0.0067 |
| rs72631343 | G | C | 0.021597 | 0.003685 | G | C | 0.0066 | 0.01 |
| rs72694393 | T | G | 0.018287 | 0.002479 | T | G | -0.0177 | 0.0095 |
| rs72789541 | A | T | 0.018236 | 0.002703 | A | T | 0.0054 | 0.0074 |
| rs72793380 | A | C | 0.02248 | 0.004032 | A | C | 0.0007 | 0.0118 |
| rs72818989 | C | T | 0.015766 | 0.002484 | C | T | -0.0064 | 0.0067 |
| rs72959041 | A | G | 0.051281 | 0.005716 | A | G | -0.0264 | 0.016 |
| rs73038384 | T | C | -0.05206 | 0.007016 | T | C | 0.0525 | 0.0199 |
| rs73234873 | T | C | -0.01499 | 0.00271 | T | C | 0.0152 | 0.0074 |
| rs73263719 | A | G | 0.019352 | 0.00348 | A | G | -0.0135 | 0.0135 |
| rs7366884 | C | T | 0.018621 | 0.002762 | C | T | -0.0055 | 0.0077 |
| rs7402977 | A | G | -0.01627 | 0.002803 | A | G | 0.0033 | 0.0075 |
| rs74502455 | G | C | -0.02087 | 0.002464 | G | C | -0.0312 | 0.0101 |
| rs74538877 | C | G | 0.031123 | 0.005498 | C | G | -0.0025 | 0.0157 |
| rs74780677 | G | A | 0.093012 | 0.010096 | G | A | 0.0665 | 0.0296 |
| rs75070309 | A | C | 0.03197 | 0.003228 | A | C | -0.0194 | 0.009 |
| rs7591567 | C | T | 0.015616 | 0.002704 | C | T | -0.0001 | 0.0072 |
| rs7731045 | C | T | -0.01422 | 0.002543 | C | T | 0.0012 | 0.0069 |
| rs77542162 | G | A | -0.19345 | 0.008216 | G | A | -0.0059 | 0.0251 |
| rs77849807 | G | A | 0.11375 | 0.00989 | G | A | -0.0144 | 0.0331 |
| rs78444263 | T | C | -0.03521 | 0.004796 | T | C | 0.0173 | 0.0139 |
| rs78961851 | C | T | -0.0248 | 0.004077 | C | T | 0.0229 | 0.0113 |
| rs79295634 | G | A | 0.03133 | 0.004938 | G | A | -0.0076 | 0.0133 |
| rs8041057 | T | C | -0.02322 | 0.002722 | T | C | 0.0084 | 0.0074 |
| rs8072215 | G | A | -0.03389 | 0.002914 | G | A | 0.0121 | 0.0078 |
| rs854796 | A | G | 0.015472 | 0.002654 | A | G | -0.0056 | 0.0073 |
| rs872926 | A | G | -0.03583 | 0.003037 | A | G | 0.0094 | 0.0083 |
| rs879620 | T | C | -0.01419 | 0.002541 | T | C | 0.0078 | 0.0069 |
| rs900400 | C | T | 0.022534 | 0.002516 | C | T | -0.0134 | 0.0068 |
| rs9265945 | G | A | -0.02345 | 0.0025 | G | A | 0.0064 | 0.0073 |
| rs930340 | A | G | -0.01888 | 0.00316 | A | G | -0.0086 | 0.0085 |
| rs9391997 | G | A | -0.01621 | 0.002476 | G | A | -0.0053 | 0.0067 |
| rs9638180 | G | A | -0.02447 | 0.003091 | G | A | 0.0095 | 0.0084 |
| rs9917677 | C | T | -0.01551 | 0.00283 | C | T | 0.0069 | 0.0077 |
| rs992367 | G | A | 0.015386 | 0.002521 | G | A | 0.0018 | 0.0068 |
| rs9976946 | T | C | 0.038971 | 0.006444 | T | C | 0.0059 | 0.0206 |

SNP, single nucleotide polymorphism; AF, atrial fibrillation; EA, effect allele; OA, other allele; SE, standard error

**Supplementary Table S6.** SNP serum albumin and SNP CAD associations (per Effect Allele) of the instruments of serum albumin after data harmonization and outlier removal

| SNP | SNP-Serum Albumin | | | | SNP-CAD | | | |
| --- | --- | --- | --- | --- | --- | --- | --- | --- |
|  | EA | OA | Beta | SE | EA | OA | Beta | SE |
| rs10004084 | C | T | 0.030092 | 0.003327 | C | T | -0.01071 | 0.011985 |
| rs10042492 | T | C | -0.01474 | 0.002478 | T | C | -0.00324 | 0.009479 |
| rs10143115 | A | G | -0.01466 | 0.002502 | A | G | 0.013643 | 0.009926 |
| rs10213692 | C | T | 0.019841 | 0.002898 | C | T | 0.011798 | 0.012509 |
| rs10236582 | C | T | -0.02217 | 0.002761 | C | T | -0.00493 | 0.010294 |
| rs1030098 | C | T | 0.020357 | 0.002543 | C | T | 0.014122 | 0.009878 |
| rs10419198 | T | C | -0.07391 | 0.002842 | T | C | 0.02123 | 0.011039 |
| rs10431419 | A | C | -0.01654 | 0.002956 | A | C | -0.00274 | 0.010836 |
| rs1043312 | G | T | 0.014211 | 0.002586 | G | T | -0.02562 | 0.009566 |
| rs10456852 | T | C | 0.026322 | 0.0037 | T | C | -0.01849 | 0.016038 |
| rs1079290 | A | T | 0.015474 | 0.002497 | A | T | -0.01623 | 0.009425 |
| rs10793127 | A | G | 0.024629 | 0.00427 | A | G | 0.009907 | 0.014845 |
| rs10863570 | C | T | 0.022492 | 0.002827 | C | T | -0.01538 | 0.011071 |
| rs10919543 | G | A | -0.01919 | 0.002669 | G | A | 0.002336 | 0.011377 |
| rs11012732 | G | A | -0.02334 | 0.002621 | G | A | 0.002305 | 0.010522 |
| rs11057273 | C | T | -0.02864 | 0.004247 | C | T | -0.01926 | 0.014268 |
| rs11074901 | A | G | 0.01473 | 0.002664 | A | G | -0.01571 | 0.010068 |
| rs11078597 | C | T | 0.065424 | 0.003154 | C | T | -0.00989 | 0.012362 |
| rs11088253 | T | C | 0.013768 | 0.002467 | T | C | 0.012274 | 0.009352 |
| rs1110659 | C | T | 0.02205 | 0.002891 | C | T | 0.029221 | 0.011398 |
| rs11208706 | G | T | 0.021997 | 0.002555 | G | T | 0.006454 | 0.009507 |
| rs11217135 | T | C | -0.02449 | 0.002478 | T | C | -0.01506 | 0.009668 |
| rs11264233 | A | G | 0.019323 | 0.002458 | A | G | -0.01915 | 0.009301 |
| rs113177823 | A | G | 0.0428 | 0.005493 | A | G | -0.01025 | 0.026819 |
| rs114949263 | C | T | 0.056977 | 0.003919 | C | T | -0.02464 | 0.01747 |
| rs11609805 | A | G | -0.02541 | 0.002866 | A | G | -0.01214 | 0.01189 |
| rs11656541 | C | G | 0.026835 | 0.00259 | C | G | 0.00182 | 0.009935 |
| rs117127664 | T | C | 0.037526 | 0.006224 | T | C | 0.007431 | 0.023419 |
| rs11736842 | T | A | 0.015911 | 0.002556 | T | A | -0.01102 | 0.009447 |
| rs117820542 | G | C | -0.04114 | 0.007446 | G | C | -0.00134 | 0.032938 |
| rs11895352 | T | C | -0.01366 | 0.002467 | T | C | 0.01377 | 0.009196 |
| rs11928797 | A | C | 0.027873 | 0.003829 | A | C | -0.00715 | 0.014313 |
| rs12215904 | T | C | -0.01996 | 0.003201 | T | C | 0.016854 | 0.012169 |
| rs1229492 | C | T | -0.01708 | 0.0028 | C | T | -0.00784 | 0.010331 |
| rs1229984 | C | T | 0.059701 | 0.008334 | C | T | 0.033254 | 0.018648 |
| rs12377600 | G | A | -0.01816 | 0.002584 | G | A | -0.00725 | 0.009455 |
| rs1260326 | C | T | -0.059 | 0.002516 | C | T | 0.003257 | 0.00962 |
| rs12710562 | G | A | 0.026315 | 0.003187 | G | A | -0.01048 | 0.013 |
| rs12815728 | C | T | 0.018075 | 0.002477 | C | T | 0.000198 | 0.009344 |
| rs12881869 | T | C | -0.04948 | 0.004787 | T | C | -0.02686 | 0.021469 |
| rs12924886 | T | A | 0.029495 | 0.003148 | T | A | 0.035779 | 0.011103 |
| rs13107325 | T | C | -0.05589 | 0.004682 | T | C | -0.00667 | 0.022347 |
| rs13108218 | G | A | -0.04425 | 0.002553 | G | A | -0.00999 | 0.010083 |
| rs13111128 | A | G | -0.01861 | 0.002714 | A | G | -0.02213 | 0.009879 |
| rs1331309 | G | T | 0.020886 | 0.002817 | G | T | -0.02455 | 0.011182 |
| rs13389219 | T | C | -0.02936 | 0.002518 | T | C | -0.02378 | 0.010119 |
| rs138833981 | G | C | -0.08581 | 0.009893 | G | C | 0.02936 | 0.049003 |
| rs139278099 | T | C | 0.062656 | 0.00619 | T | C | 0.013606 | 0.033393 |
| rs139974673 | C | T | 0.15127 | 0.00788 | C | T | 0.004151 | 0.030963 |
| rs147651823 | A | C | -0.03493 | 0.005891 | A | C | 0.020674 | 0.020265 |
| rs149092986 | C | T | -0.0549 | 0.008357 | C | T | 0.008597 | 0.045483 |
| rs1497406 | G | A | 0.025056 | 0.002491 | G | A | 0.027339 | 0.009513 |
| rs150783681 | C | G | -0.11931 | 0.008754 | C | G | 0.033826 | 0.039945 |
| rs1532085 | G | A | 0.014174 | 0.002528 | G | A | -0.01812 | 0.009354 |
| rs157936 | G | T | -0.01904 | 0.002696 | G | T | -0.01169 | 0.009999 |
| rs1593357 | T | C | -0.02036 | 0.002864 | T | C | -0.0053 | 0.010223 |
| rs16950612 | G | A | -0.02544 | 0.003943 | G | A | 0.004117 | 0.016312 |
| rs17023530 | A | T | -0.03838 | 0.005752 | A | T | 0.001437 | 0.021234 |
| rs17580 | A | T | 0.094752 | 0.005709 | A | T | -0.03128 | 0.027042 |
| rs1782455 | A | G | 0.019719 | 0.00334 | A | G | -0.00159 | 0.011887 |
| rs1791936 | A | G | 0.021346 | 0.002518 | A | G | -0.01682 | 0.009815 |
| rs1801282 | G | C | -0.05066 | 0.003774 | G | C | -0.00113 | 0.014127 |
| rs1880241 | G | A | 0.015573 | 0.002469 | G | A | -0.00376 | 0.009698 |
| rs1886839 | G | A | 0.014096 | 0.002513 | G | A | -0.00983 | 0.00967 |
| rs198426 | T | C | -0.01691 | 0.002607 | T | C | -0.00485 | 0.010637 |
| rs1986133 | T | C | 0.016108 | 0.002656 | T | C | 0.016024 | 0.009863 |
| rs1998528 | A | G | 0.015217 | 0.002586 | A | G | -0.00531 | 0.009885 |
| rs2060658 | C | T | 0.016588 | 0.002478 | C | T | -0.01263 | 0.009515 |
| rs2072442 | G | C | 0.014529 | 0.002492 | G | C | 0.005808 | 0.009453 |
| rs2169387 | G | A | 0.057547 | 0.004108 | G | A | 0.005655 | 0.015822 |
| rs2200061 | A | G | -0.0165 | 0.003013 | A | G | -0.0198 | 0.033381 |
| rs2227827 | T | C | -0.05124 | 0.005762 | T | C | 0.020251 | 0.025209 |
| rs2267867 | G | A | -0.01609 | 0.002896 | G | A | -0.00836 | 0.010828 |
| rs2303695 | T | C | -0.02567 | 0.002582 | T | C | -0.00849 | 0.009665 |
| rs2304130 | G | A | 0.02707 | 0.004395 | G | A | -0.02493 | 0.016451 |
| rs234043 | C | T | 0.016912 | 0.002733 | C | T | -0.00739 | 0.010439 |
| rs2638315 | C | G | 0.035351 | 0.00318 | C | G | -0.02859 | 0.012378 |
| rs2702571 | A | T | 0.016766 | 0.00258 | A | T | -0.00731 | 0.01012 |
| rs2710804 | C | T | -0.01647 | 0.002545 | C | T | -0.00152 | 0.009797 |
| rs2785172 | A | G | -0.01916 | 0.002527 | A | G | -0.00082 | 0.00963 |
| rs2820446 | G | C | -0.02399 | 0.002685 | G | C | -0.00702 | 0.010301 |
| rs28687959 | T | C | 0.016421 | 0.002478 | T | C | -0.00582 | 0.009211 |
| rs28688002 | A | T | 0.027203 | 0.002951 | A | T | -0.01123 | 0.010581 |
| rs28929474 | T | C | 0.26514 | 0.008734 | T | C | -0.14659 | 0.045171 |
| rs3099371 | T | C | 0.015872 | 0.002512 | T | C | 0.00363 | 0.009328 |
| rs34562254 | A | G | -0.03754 | 0.004139 | A | G | -0.00449 | 0.014058 |
| rs34931250 | T | C | 0.03341 | 0.005134 | T | C | 0.065473 | 0.025378 |
| rs35123414 | C | T | 0.021697 | 0.003622 | C | T | -0.01589 | 0.015448 |
| rs35676551 | A | C | -0.0379 | 0.00554 | A | C | -0.02342 | 0.030269 |
| rs36090025 | C | A | 0.017388 | 0.002687 | C | A | 0.034959 | 0.010543 |
| rs3740688 | T | G | 0.024193 | 0.002475 | T | G | 0.00662 | 0.009529 |
| rs3768321 | T | G | -0.02842 | 0.003099 | T | G | 0.015352 | 0.013117 |
| rs378740 | A | C | 0.01565 | 0.002779 | A | C | 0.010584 | 0.010151 |
| rs3810484 | G | A | 0.014777 | 0.002477 | G | A | 0.012948 | 0.012171 |
| rs390801 | C | T | 0.021268 | 0.002802 | C | T | -0.00253 | 0.010104 |
| rs4327724 | T | C | 0.031296 | 0.004594 | T | C | -0.02564 | 0.019825 |
| rs4410790 | C | T | 0.015033 | 0.002557 | C | T | 0.027305 | 0.009664 |
| rs4458838 | A | G | -0.01566 | 0.002581 | A | G | 0.005936 | 0.009913 |
| rs4499445 | T | G | -0.02174 | 0.002604 | T | G | 0.02548 | 0.01035 |
| rs45439091 | T | G | 0.031713 | 0.004747 | T | G | 0.034632 | 0.014804 |
| rs45512696 | T | C | 0.080056 | 0.003232 | T | C | 0.012873 | 0.013882 |
| rs459193 | G | A | 0.022894 | 0.002978 | G | A | 0.026366 | 0.010157 |
| rs4711399 | T | C | 0.021968 | 0.002966 | T | C | -0.02602 | 0.011141 |
| rs473919 | G | C | -0.01888 | 0.003124 | G | C | 0.003766 | 0.011811 |
| rs4782568 | G | C | -0.01843 | 0.002489 | G | C | 0.017848 | 0.010608 |
| rs4790875 | T | C | -0.02071 | 0.002513 | T | C | 0.027743 | 0.009535 |
| rs4796307 | A | T | -0.02815 | 0.003001 | A | T | -0.02347 | 0.011507 |
| rs4804413 | T | C | 0.017594 | 0.002488 | T | C | 0.002245 | 0.009343 |
| rs4805129 | C | T | -0.02111 | 0.002546 | C | T | -0.00435 | 0.009817 |
| rs4805881 | C | A | -0.02704 | 0.002609 | C | A | -0.02834 | 0.009695 |
| rs4833945 | C | T | 0.021705 | 0.003854 | C | T | 0.01191 | 0.014492 |
| rs4946811 | C | A | 0.015897 | 0.002567 | C | A | 0.020652 | 0.010089 |
| rs55696240 | A | G | 0.017035 | 0.002551 | A | G | 0.019272 | 0.009881 |
| rs55722786 | T | G | 0.028148 | 0.002735 | T | G | 0.013573 | 0.011986 |
| rs55724869 | C | G | 0.014174 | 0.002501 | C | G | 0.000317 | 0.010412 |
| rs55846720 | A | G | -0.0148 | 0.002479 | A | G | 0.03223 | 0.009673 |
| rs56094005 | G | A | -0.04654 | 0.006021 | G | A | 0.013201 | 0.027776 |
| rs56188865 | C | T | 0.016247 | 0.002556 | C | T | -0.01525 | 0.009615 |
| rs58558667 | T | G | 0.023041 | 0.002553 | T | G | -0.00116 | 0.009671 |
| rs58579887 | C | T | 0.018348 | 0.002522 | C | T | -0.00144 | 0.010165 |
| rs59842359 | T | C | -0.02257 | 0.003073 | T | C | -0.01637 | 0.011339 |
| rs59916403 | T | G | -0.0243 | 0.002576 | T | G | 0.002497 | 0.009855 |
| rs6031847 | T | C | -0.02127 | 0.002777 | T | C | 0.007238 | 0.010824 |
| rs60644673 | T | G | -0.01754 | 0.003122 | T | G | -0.02203 | 0.013249 |
| rs6065295 | T | C | 0.015019 | 0.002491 | T | C | 0.0078 | 0.009566 |
| rs61817641 | T | C | -0.01821 | 0.002782 | T | C | 0.023094 | 0.011398 |
| rs61983272 | C | G | -0.03286 | 0.002844 | C | G | 0.032393 | 0.014272 |
| rs62053895 | A | G | -0.01385 | 0.002519 | A | G | 0.002523 | 0.009867 |
| rs62235071 | T | C | -0.04014 | 0.00522 | T | C | 0.013991 | 0.023791 |
| rs631695 | G | T | 0.015489 | 0.002494 | G | T | 0.005329 | 0.009617 |
| rs6490409 | G | A | -0.01943 | 0.003511 | G | A | 0.000562 | 0.011563 |
| rs6549406 | G | A | -0.01898 | 0.002788 | G | A | -0.01446 | 0.010603 |
| rs6567095 | A | G | 0.01941 | 0.002483 | A | G | -0.00424 | 0.009588 |
| rs667172 | A | G | 0.018923 | 0.002725 | A | G | 0.005963 | 0.010139 |
| rs6682695 | C | G | -0.02397 | 0.003321 | C | G | 0.000449 | 0.0125 |
| rs6693993 | C | G | 0.023539 | 0.00247 | C | G | -0.02505 | 0.009282 |
| rs6734238 | G | A | -0.016 | 0.002507 | G | A | -0.02277 | 0.009957 |
| rs673751 | C | A | -0.01529 | 0.002638 | C | A | 0.001375 | 0.009678 |
| rs67694436 | T | C | 0.01512 | 0.002586 | T | C | -0.00289 | 0.010873 |
| rs6794370 | C | A | -0.01926 | 0.003127 | C | A | -0.00123 | 0.011185 |
| rs6860245 | C | G | -0.02143 | 0.002858 | C | G | -0.01062 | 0.010646 |
| rs6871748 | C | T | 0.015224 | 0.002757 | C | T | -0.00096 | 0.010707 |
| rs6897617 | A | G | 0.018249 | 0.002716 | A | G | -0.01055 | 0.011001 |
| rs6912315 | T | C | 0.031344 | 0.005494 | T | C | -0.00536 | 0.018597 |
| rs6970593 | A | G | -0.0255 | 0.002472 | A | G | 0.004075 | 0.009468 |
| rs7031621 | A | G | -0.01438 | 0.002461 | A | G | -0.00622 | 0.010266 |
| rs72631343 | G | C | 0.021597 | 0.003685 | G | C | -0.01414 | 0.013097 |
| rs72694393 | T | G | 0.018287 | 0.002479 | T | G | 0.009805 | 0.01029 |
| rs72789541 | A | T | 0.018236 | 0.002703 | A | T | 0.014604 | 0.01018 |
| rs72793380 | A | C | 0.02248 | 0.004032 | A | C | -0.00392 | 0.020447 |
| rs72818989 | C | T | 0.015766 | 0.002484 | C | T | 0.009929 | 0.009699 |
| rs72959041 | A | G | 0.051281 | 0.005716 | A | G | 0.013346 | 0.026655 |
| rs73038384 | T | C | -0.05206 | 0.007016 | T | C | 0.022922 | 0.034457 |
| rs73234873 | T | C | -0.01499 | 0.00271 | T | C | -0.01185 | 0.011789 |
| rs73263719 | A | G | 0.019352 | 0.00348 | A | G | -0.0069 | 0.013031 |
| rs7366884 | C | T | 0.018621 | 0.002762 | C | T | -0.00073 | 0.01092 |
| rs7402977 | A | G | -0.01627 | 0.002803 | A | G | -0.00624 | 0.010649 |
| rs74502455 | G | C | -0.02087 | 0.002464 | G | C | 0.010805 | 0.009463 |
| rs74538877 | C | G | 0.031123 | 0.005498 | C | G | -0.00797 | 0.026199 |
| rs74780677 | G | A | 0.093012 | 0.010096 | G | A | -0.04928 | 0.039569 |
| rs75070309 | A | C | 0.03197 | 0.003228 | A | C | 0.001554 | 0.013501 |
| rs7591567 | C | T | 0.015616 | 0.002704 | C | T | -0.03029 | 0.010414 |
| rs76895963 | G | T | -0.07122 | 0.009449 | G | T | -6.7E-05 | 0.047423 |
| rs7731045 | C | T | -0.01422 | 0.002543 | C | T | -0.01032 | 0.00991 |
| rs77542162 | G | A | -0.19345 | 0.008216 | G | A | 0.015801 | 0.043855 |
| rs78444263 | T | C | -0.03521 | 0.004796 | T | C | 0.004322 | 0.022957 |
| rs78961851 | C | T | -0.0248 | 0.004077 | C | T | -0.02399 | 0.019684 |
| rs79295634 | G | A | 0.03133 | 0.004938 | G | A | -0.04106 | 0.016416 |
| rs800545 | G | A | -0.01617 | 0.002833 | G | A | 0.002498 | 0.010403 |
| rs8041057 | T | C | -0.02322 | 0.002722 | T | C | -0.01768 | 0.010099 |
| rs854796 | A | G | 0.015472 | 0.002654 | A | G | 0.004347 | 0.009991 |
| rs872926 | A | G | -0.03583 | 0.003037 | A | G | 0.014971 | 0.012071 |
| rs879620 | T | C | -0.01419 | 0.002541 | T | C | -0.00294 | 0.009732 |
| rs900400 | C | T | 0.022534 | 0.002516 | C | T | 0.012519 | 0.009613 |
| rs9265945 | G | A | -0.02345 | 0.0025 | G | A | -0.01261 | 0.010975 |
| rs930340 | A | G | -0.01888 | 0.00316 | A | G | 0.011981 | 0.012066 |
| rs9391997 | G | A | -0.01621 | 0.002476 | G | A | 0.009369 | 0.009383 |
| rs9638180 | G | A | -0.02447 | 0.003091 | G | A | -0.01215 | 0.012792 |
| rs9917677 | C | T | -0.01551 | 0.00283 | C | T | -0.00057 | 0.01146 |
| rs992367 | G | A | 0.015386 | 0.002521 | G | A | 0.012238 | 0.009591 |
| rs9976946 | T | C | 0.038971 | 0.006444 | T | C | 0.049254 | 0.032988 |

SNP, single nucleotide polymorphism; CAD, coronary artery disease; EA, effect allele; OA, other allele; SE, standard error

**Supplementary Table S7.** SNP serum albumin and SNP T2DM associations (per Effect Allele) of the instruments of serum albumin after data harmonization and outlier removal

| SNP | SNP-Serum Albumin | | | | SNP-T2DM | | | |
| --- | --- | --- | --- | --- | --- | --- | --- | --- |
|  | EA | OA | Beta | SE | EA | OA | Beta | SE |
| rs10004084 | C | T | 0.030092 | 0.003327 | C | T | 0.0168 | 0.0104 |
| rs10042492 | T | C | -0.01474 | 0.002478 | T | C | -0.0099 | 0.0079 |
| rs10143115 | A | G | -0.01466 | 0.002502 | A | G | 0.0078 | 0.0078 |
| rs1030098 | C | T | 0.020357 | 0.002543 | C | T | -0.0167 | 0.0074 |
| rs10431419 | A | C | -0.01654 | 0.002956 | A | C | -0.0006 | 0.0093 |
| rs1043312 | G | T | 0.014211 | 0.002586 | G | T | -0.0025 | 0.0083 |
| rs1079290 | A | T | 0.015474 | 0.002497 | A | T | -0.0018 | 0.008 |
| rs10793127 | A | G | 0.024629 | 0.00427 | A | G | 0.0208 | 0.0135 |
| rs10863570 | C | T | 0.022492 | 0.002827 | C | T | -0.0094 | 0.009 |
| rs11012732 | G | A | -0.02334 | 0.002621 | G | A | -0.0022 | 0.0083 |
| rs11057273 | C | T | -0.02864 | 0.004247 | C | T | -0.0036 | 0.0134 |
| rs11074901 | A | G | 0.01473 | 0.002664 | A | G | 0.0045 | 0.0084 |
| rs11088253 | T | C | 0.013768 | 0.002467 | T | C | -0.0002 | 0.0079 |
| rs1110659 | C | T | 0.02205 | 0.002891 | C | T | -0.0269 | 0.0093 |
| rs11208706 | G | T | 0.021997 | 0.002555 | G | T | -0.0075 | 0.0081 |
| rs11217135 | T | C | -0.02449 | 0.002478 | T | C | -0.0095 | 0.0079 |
| rs11264233 | A | G | 0.019323 | 0.002458 | A | G | -0.0088 | 0.0077 |
| rs11609805 | A | G | -0.02541 | 0.002866 | A | G | 0.0001 | 0.0092 |
| rs11656541 | C | G | 0.026835 | 0.00259 | C | G | -0.0069 | 0.0082 |
| rs117127664 | T | C | 0.037526 | 0.006224 | T | C | 0.0109 | 0.0203 |
| rs11736842 | T | A | 0.015911 | 0.002556 | T | A | 0.0097 | 0.0082 |
| rs11895352 | T | C | -0.01366 | 0.002467 | T | C | -0.0047 | 0.0078 |
| rs11928797 | A | C | 0.027873 | 0.003829 | A | C | -0.0072 | 0.0122 |
| rs12215904 | T | C | -0.01996 | 0.003201 | T | C | 0.0057 | 0.0102 |
| rs1229492 | C | T | -0.01708 | 0.0028 | C | T | -0.0098 | 0.0082 |
| rs12377600 | G | A | -0.01816 | 0.002584 | G | A | 0.0064 | 0.0082 |
| rs12563096 | A | G | 0.01427 | 0.002614 | A | G | 0.011 | 0.0086 |
| rs12815728 | C | T | 0.018075 | 0.002477 | C | T | -0.0172 | 0.0079 |
| rs12881869 | T | C | -0.04948 | 0.004787 | T | C | -0.008 | 0.0155 |
| rs12924886 | T | A | 0.029495 | 0.003148 | T | A | 0.0238 | 0.0099 |
| rs13111128 | A | G | -0.01861 | 0.002714 | A | G | -0.0098 | 0.0082 |
| rs1331309 | G | T | 0.020886 | 0.002817 | G | T | 0.0072 | 0.0082 |
| rs1497406 | G | A | 0.025056 | 0.002491 | G | A | -0.0041 | 0.008 |
| rs1500187 | G | A | -0.02369 | 0.002472 | G | A | 0.0201 | 0.0079 |
| rs1532085 | G | A | 0.014174 | 0.002528 | G | A | 0.0229 | 0.0074 |
| rs157936 | G | T | -0.01904 | 0.002696 | G | T | -0.0025 | 0.0085 |
| rs1593357 | T | C | -0.02036 | 0.002864 | T | C | -0.0324 | 0.0092 |
| rs16950612 | G | A | -0.02544 | 0.003943 | G | A | 0.0246 | 0.0124 |
| rs17023530 | A | T | -0.03838 | 0.005752 | A | T | -0.0331 | 0.0185 |
| rs1782455 | A | G | 0.019719 | 0.00334 | A | G | 0.0165 | 0.0106 |
| rs1880241 | G | A | 0.015573 | 0.002469 | G | A | -0.0068 | 0.0078 |
| rs1886839 | G | A | 0.014096 | 0.002513 | G | A | -0.0066 | 0.008 |
| rs198426 | T | C | -0.01691 | 0.002607 | T | C | 0.0158 | 0.0083 |
| rs1986133 | T | C | 0.016108 | 0.002656 | T | C | 0.0056 | 0.0085 |
| rs1998528 | A | G | 0.015217 | 0.002586 | A | G | -0.0013 | 0.0082 |
| rs2060658 | C | T | 0.016588 | 0.002478 | C | T | 0.0212 | 0.0072 |
| rs2072442 | G | C | 0.014529 | 0.002492 | G | C | -0.0037 | 0.0079 |
| rs2115868 | A | T | -0.02438 | 0.003343 | A | T | -0.0021 | 0.0107 |
| rs2200061 | A | G | -0.0165 | 0.003013 | A | G | -0.0109 | 0.0096 |
| rs2227827 | T | C | -0.05124 | 0.005762 | T | C | -0.0205 | 0.019 |
| rs2267867 | G | A | -0.01609 | 0.002896 | G | A | 0.0054 | 0.0093 |
| rs2303695 | T | C | -0.02567 | 0.002582 | T | C | 0.0066 | 0.0082 |
| rs234043 | C | T | 0.016912 | 0.002733 | C | T | 0.0154 | 0.0087 |
| rs2638315 | C | G | 0.035351 | 0.00318 | C | G | -0.0031 | 0.0099 |
| rs2702571 | A | T | 0.016766 | 0.00258 | A | T | -0.0091 | 0.0081 |
| rs2710804 | C | T | -0.01647 | 0.002545 | C | T | 0.0029 | 0.0081 |
| rs2785172 | A | G | -0.01916 | 0.002527 | A | G | -0.0039 | 0.0079 |
| rs28601761 | G | C | -0.01996 | 0.002523 | G | C | 0.0072 | 0.008 |
| rs28687959 | T | C | 0.016421 | 0.002478 | T | C | 0.0042 | 0.0079 |
| rs28688002 | A | T | 0.027203 | 0.002951 | A | T | 0.0027 | 0.0094 |
| rs3099371 | T | C | 0.015872 | 0.002512 | T | C | -0.0014 | 0.0079 |
| rs34562254 | A | G | -0.03754 | 0.004139 | A | G | -0.0042 | 0.013 |
| rs3740688 | T | G | 0.024193 | 0.002475 | T | G | -0.0167 | 0.008 |
| rs390801 | C | T | 0.021268 | 0.002802 | C | T | 0.0014 | 0.0091 |
| rs4410790 | C | T | 0.015033 | 0.002557 | C | T | 0.0157 | 0.0086 |
| rs4458838 | A | G | -0.01566 | 0.002581 | A | G | -0.0129 | 0.0083 |
| rs4499445 | T | G | -0.02174 | 0.002604 | T | G | -0.0047 | 0.0082 |
| rs45439091 | T | G | 0.031713 | 0.004747 | T | G | -0.0339 | 0.0151 |
| rs45512696 | T | C | 0.080056 | 0.003232 | T | C | -0.0005 | 0.0105 |
| rs4711399 | T | C | 0.021968 | 0.002966 | T | C | 0.0064 | 0.0094 |
| rs473919 | G | C | -0.01888 | 0.003124 | G | C | 0.0161 | 0.0099 |
| rs4790875 | T | C | -0.02071 | 0.002513 | T | C | -0.0187 | 0.008 |
| rs4796307 | A | T | -0.02815 | 0.003001 | A | T | -0.0072 | 0.0096 |
| rs4804413 | T | C | 0.017594 | 0.002488 | T | C | 0.0272 | 0.0084 |
| rs4833945 | C | T | 0.021705 | 0.003854 | C | T | 0.0074 | 0.0119 |
| rs4970834 | T | C | -0.02539 | 0.003169 | T | C | 0.0112 | 0.0092 |
| rs55696240 | A | G | 0.017035 | 0.002551 | A | G | 0.0058 | 0.0082 |
| rs55722786 | T | G | 0.028148 | 0.002735 | T | G | 0.0051 | 0.0087 |
| rs55724869 | C | G | 0.014174 | 0.002501 | C | G | 0.0026 | 0.008 |
| rs56188865 | C | T | 0.016247 | 0.002556 | C | T | -0.0046 | 0.0081 |
| rs58558667 | T | G | 0.023041 | 0.002553 | T | G | 0.0046 | 0.0081 |
| rs59842359 | T | C | -0.02257 | 0.003073 | T | C | -0.0051 | 0.0098 |
| rs59916403 | T | G | -0.0243 | 0.002576 | T | G | -0.0025 | 0.0082 |
| rs6031847 | T | C | -0.02127 | 0.002777 | T | C | -0.0115 | 0.0088 |
| rs60644673 | T | G | -0.01754 | 0.003122 | T | G | 0.0098 | 0.0099 |
| rs6065295 | T | C | 0.015019 | 0.002491 | T | C | 0.0016 | 0.008 |
| rs61817641 | T | C | -0.01821 | 0.002782 | T | C | -0.0094 | 0.009 |
| rs62053895 | A | G | -0.01385 | 0.002519 | A | G | 0.0064 | 0.0081 |
| rs62235071 | T | C | -0.04014 | 0.00522 | T | C | 0.0285 | 0.018 |
| rs6490409 | G | A | -0.01943 | 0.003511 | G | A | -0.0013 | 0.0111 |
| rs6549406 | G | A | -0.01898 | 0.002788 | G | A | 0.0046 | 0.0089 |
| rs6567095 | A | G | 0.01941 | 0.002483 | A | G | 0.0042 | 0.0079 |
| rs667172 | A | G | 0.018923 | 0.002725 | A | G | -0.007 | 0.0087 |
| rs6693993 | C | G | 0.023539 | 0.00247 | C | G | 0.0088 | 0.0079 |
| rs6734238 | G | A | -0.016 | 0.002507 | G | A | 0.0037 | 0.0079 |
| rs673751 | C | A | -0.01529 | 0.002638 | C | A | 0.0012 | 0.0084 |
| rs6793835 | A | G | 0.016418 | 0.002803 | A | G | 0.028 | 0.0088 |
| rs6860245 | C | G | -0.02143 | 0.002858 | C | G | -0.0347 | 0.0091 |
| rs6871748 | C | T | 0.015224 | 0.002757 | C | T | 0.0029 | 0.0088 |
| rs6897617 | A | G | 0.018249 | 0.002716 | A | G | 0.0112 | 0.0086 |
| rs6912315 | T | C | 0.031344 | 0.005494 | T | C | -0.0159 | 0.0173 |
| rs6970593 | A | G | -0.0255 | 0.002472 | A | G | -0.0062 | 0.0078 |
| rs72631343 | G | C | 0.021597 | 0.003685 | G | C | -0.0025 | 0.0117 |
| rs72789541 | A | T | 0.018236 | 0.002703 | A | T | -0.015 | 0.0086 |
| rs73234873 | T | C | -0.01499 | 0.00271 | T | C | 0.0118 | 0.0086 |
| rs73263719 | A | G | 0.019352 | 0.00348 | A | G | -0.015 | 0.0121 |
| rs7366884 | C | T | 0.018621 | 0.002762 | C | T | 0.0083 | 0.0088 |
| rs7402977 | A | G | -0.01627 | 0.002803 | A | G | -0.0261 | 0.0092 |
| rs75070309 | A | C | 0.03197 | 0.003228 | A | C | -0.0036 | 0.0104 |
| rs7591567 | C | T | 0.015616 | 0.002704 | C | T | -0.0064 | 0.0085 |
| rs7731045 | C | T | -0.01422 | 0.002543 | C | T | -0.0005 | 0.0081 |
| rs79295634 | G | A | 0.03133 | 0.004938 | G | A | -0.0315 | 0.0157 |
| rs800545 | G | A | -0.01617 | 0.002833 | G | A | 0.0149 | 0.009 |
| rs8041057 | T | C | -0.02322 | 0.002722 | T | C | -0.0261 | 0.0086 |
| rs854796 | A | G | 0.015472 | 0.002654 | A | G | -0.0157 | 0.0091 |
| rs872926 | A | G | -0.03583 | 0.003037 | A | G | 0.0051 | 0.0098 |
| rs900400 | C | T | 0.022534 | 0.002516 | C | T | 0.0163 | 0.008 |
| rs930340 | A | G | -0.01888 | 0.00316 | A | G | 0.0091 | 0.0092 |
| rs9391997 | G | A | -0.01621 | 0.002476 | G | A | 0.0117 | 0.0078 |
| rs9638180 | G | A | -0.02447 | 0.003091 | G | A | 0.0168 | 0.0098 |
| rs992367 | G | A | 0.015386 | 0.002521 | G | A | -0.0181 | 0.0081 |
| rs9976946 | T | C | 0.038971 | 0.006444 | T | C | 0.0141 | 0.0223 |

SNP, single nucleotide polymorphism; T2DM, Type 2 diabetes; EA, effect allele; OA, other allele; SE, standard error

**Supplementary Table S8.** SNP serum albumin and SNP PHD associations (per Effect Allele) of the instruments of serum albumin after data harmonization and outlier removal

| SNP | SNP-Serum Albumin | | | | SNP-PHD | | | |
| --- | --- | --- | --- | --- | --- | --- | --- | --- |
|  | EA | OA | Beta | SE | EA | OA | Beta | SE |
| rs10004084 | C | T | 0.030092 | 0.003327 | C | T | -0.0534 | 0.0324 |
| rs10042492 | T | C | -0.01474 | 0.002478 | T | C | 0.0251 | 0.0219 |
| rs10143115 | A | G | -0.01466 | 0.002502 | A | G | 0.0173 | 0.022 |
| rs10213692 | C | T | 0.019841 | 0.002898 | C | T | 0.0272 | 0.0291 |
| rs10236582 | C | T | -0.02217 | 0.002761 | C | T | -0.0401 | 0.0261 |
| rs1030098 | C | T | 0.020357 | 0.002543 | C | T | -0.0288 | 0.0234 |
| rs10419198 | T | C | -0.07391 | 0.002842 | T | C | -0.0125 | 0.0249 |
| rs10431419 | A | C | -0.01654 | 0.002956 | A | C | 0.0164 | 0.0245 |
| rs1043312 | G | T | 0.014211 | 0.002586 | G | T | -0.0067 | 0.0228 |
| rs10456852 | T | C | 0.026322 | 0.0037 | T | C | -0.0203 | 0.0373 |
| rs1079290 | A | T | 0.015474 | 0.002497 | A | T | -0.0008 | 0.0219 |
| rs10793127 | A | G | 0.024629 | 0.00427 | A | G | -0.0154 | 0.0355 |
| rs10863570 | C | T | 0.022492 | 0.002827 | C | T | 0.0009 | 0.0242 |
| rs10919543 | G | A | -0.01919 | 0.002669 | G | A | -0.0026 | 0.0249 |
| rs11012732 | G | A | -0.02334 | 0.002621 | G | A | 0.0251 | 0.0235 |
| rs11057273 | C | T | -0.02864 | 0.004247 | C | T | -0.0693 | 0.0345 |
| rs11074901 | A | G | 0.01473 | 0.002664 | A | G | 0.0482 | 0.0228 |
| rs11078597 | C | T | 0.065424 | 0.003154 | C | T | -0.016 | 0.0279 |
| rs11088253 | T | C | 0.013768 | 0.002467 | T | C | 0.0212 | 0.0218 |
| rs1110659 | C | T | 0.02205 | 0.002891 | C | T | -0.0064 | 0.0276 |
| rs11208706 | G | T | 0.021997 | 0.002555 | G | T | 0.0166 | 0.0219 |
| rs11217135 | T | C | -0.02449 | 0.002478 | T | C | -0.0363 | 0.0219 |
| rs11264233 | A | G | 0.019323 | 0.002458 | A | G | 0.0237 | 0.0219 |
| rs113177823 | A | G | 0.0428 | 0.005493 | A | G | -0.1006 | 0.0459 |
| rs114949263 | C | T | 0.056977 | 0.003919 | C | T | -0.0313 | 0.0406 |
| rs11589479 | A | G | 0.045527 | 0.003316 | A | G | -0.058 | 0.0316 |
| rs11609805 | A | G | -0.02541 | 0.002866 | A | G | 0.0201 | 0.0256 |
| rs11656541 | C | G | 0.026835 | 0.00259 | C | G | 0.0442 | 0.0221 |
| rs117127664 | T | C | 0.037526 | 0.006224 | T | C | 0.0653 | 0.0423 |
| rs11736842 | T | A | 0.015911 | 0.002556 | T | A | -0.0145 | 0.0221 |
| rs117820542 | G | C | -0.04114 | 0.007446 | G | C | -0.0323 | 0.0953 |
| rs11895352 | T | C | -0.01366 | 0.002467 | T | C | -0.0136 | 0.0222 |
| rs11928797 | A | C | 0.027873 | 0.003829 | A | C | 0.0157 | 0.0338 |
| rs12215904 | T | C | -0.01996 | 0.003201 | T | C | -0.0191 | 0.0299 |
| rs1229492 | C | T | -0.01708 | 0.0028 | C | T | 0.0447 | 0.025 |
| rs1229984 | C | T | 0.059701 | 0.008334 | C | T | 0.1065 | 0.149 |
| rs12377600 | G | A | -0.01816 | 0.002584 | G | A | 0.0093 | 0.0235 |
| rs12563096 | A | G | 0.01427 | 0.002614 | A | G | -0.0036 | 0.024 |
| rs1260326 | C | T | -0.059 | 0.002516 | C | T | -0.0211 | 0.023 |
| rs12710562 | G | A | 0.026315 | 0.003187 | G | A | -0.0329 | 0.0296 |
| rs12815728 | C | T | 0.018075 | 0.002477 | C | T | 0.0315 | 0.0219 |
| rs12881869 | T | C | -0.04948 | 0.004787 | T | C | 0.0581 | 0.0372 |
| rs12924886 | T | A | 0.029495 | 0.003148 | T | A | 0.0384 | 0.0276 |
| rs13107325 | T | C | -0.05589 | 0.004682 | T | C | 0.0368 | 0.0921 |
| rs13108218 | G | A | -0.04425 | 0.002553 | G | A | -0.0013 | 0.0234 |
| rs13111128 | A | G | -0.01861 | 0.002714 | A | G | -0.0259 | 0.0228 |
| rs1331309 | G | T | 0.020886 | 0.002817 | G | T | 0.0388 | 0.0231 |
| rs13389219 | T | C | -0.02936 | 0.002518 | T | C | 0.009 | 0.023 |
| rs138833981 | G | C | -0.08581 | 0.009893 | G | C | 0.066 | 0.1154 |
| rs139278099 | T | C | 0.062656 | 0.00619 | T | C | -0.106 | 0.1046 |
| rs139974673 | C | T | 0.15127 | 0.00788 | C | T | -0.1645 | 0.1515 |
| rs147651823 | A | C | -0.03493 | 0.005891 | A | C | 0.0413 | 0.0537 |
| rs149092986 | C | T | -0.0549 | 0.008357 | C | T | 0.1945 | 0.0792 |
| rs1497406 | G | A | 0.025056 | 0.002491 | G | A | -0.0147 | 0.0234 |
| rs1500187 | G | A | -0.02369 | 0.002472 | G | A | -0.012 | 0.0219 |
| rs150783681 | C | G | -0.11931 | 0.008754 | C | G | 0.0083 | 0.0842 |
| rs1532085 | G | A | 0.014174 | 0.002528 | G | A | -0.0076 | 0.0221 |
| rs157936 | G | T | -0.01904 | 0.002696 | G | T | 0.0492 | 0.0225 |
| rs1593357 | T | C | -0.02036 | 0.002864 | T | C | 0.0273 | 0.0274 |
| rs16950612 | G | A | -0.02544 | 0.003943 | G | A | 0.1052 | 0.0408 |
| rs17023530 | A | T | -0.03838 | 0.005752 | A | T | 0.0786 | 0.0538 |
| rs17580 | A | T | 0.094752 | 0.005709 | A | T | -0.0901 | 0.1154 |
| rs1782455 | A | G | 0.019719 | 0.00334 | A | G | -0.0232 | 0.029 |
| rs1791936 | A | G | 0.021346 | 0.002518 | A | G | 0.0093 | 0.0226 |
| rs1801282 | G | C | -0.05066 | 0.003774 | G | C | -0.0207 | 0.029 |
| rs1880241 | G | A | 0.015573 | 0.002469 | G | A | 0.0269 | 0.0224 |
| rs1886839 | G | A | 0.014096 | 0.002513 | G | A | 0.0203 | 0.022 |
| rs198426 | T | C | -0.01691 | 0.002607 | T | C | 0.0198 | 0.0238 |
| rs1986133 | T | C | 0.016108 | 0.002656 | T | C | 0.0167 | 0.0249 |
| rs1998528 | A | G | 0.015217 | 0.002586 | A | G | -0.0537 | 0.023 |
| rs2060658 | C | T | 0.016588 | 0.002478 | C | T | -0.0358 | 0.0219 |
| rs2072442 | G | C | 0.014529 | 0.002492 | G | C | -0.0149 | 0.0223 |
| rs2115868 | A | T | -0.02438 | 0.003343 | A | T | -0.0128 | 0.0336 |
| rs2169387 | G | A | 0.057547 | 0.004108 | G | A | -0.0432 | 0.0326 |
| rs2200061 | A | G | -0.0165 | 0.003013 | A | G | -0.0512 | 0.0244 |
| rs2227827 | T | C | -0.05124 | 0.005762 | T | C | -0.0341 | 0.0543 |
| rs2267867 | G | A | -0.01609 | 0.002896 | G | A | 0.0121 | 0.0248 |
| rs2303695 | T | C | -0.02567 | 0.002582 | T | C | 0.0368 | 0.0225 |
| rs2304130 | G | A | 0.02707 | 0.004395 | G | A | 0.0285 | 0.0468 |
| rs234043 | C | T | 0.016912 | 0.002733 | C | T | -0.0023 | 0.0249 |
| rs2638315 | C | G | 0.035351 | 0.00318 | C | G | -0.0467 | 0.0284 |
| rs2702571 | A | T | 0.016766 | 0.00258 | A | T | 0.0452 | 0.0244 |
| rs2710804 | C | T | -0.01647 | 0.002545 | C | T | 0.0112 | 0.0228 |
| rs2785172 | A | G | -0.01916 | 0.002527 | A | G | -0.0002 | 0.0226 |
| rs2820446 | G | C | -0.02399 | 0.002685 | G | C | 0.0107 | 0.0241 |
| rs28601761 | G | C | -0.01996 | 0.002523 | G | C | -0.0018 | 0.0222 |
| rs28687959 | T | C | 0.016421 | 0.002478 | T | C | 0.0247 | 0.0222 |
| rs28688002 | A | T | 0.027203 | 0.002951 | A | T | -0.0282 | 0.0249 |
| rs2869876 | A | C | 0.017918 | 0.003186 | A | C | -0.0231 | 0.0263 |
| rs2972145 | C | T | 0.021428 | 0.002573 | C | T | 0.0152 | 0.0227 |
| rs3099371 | T | C | 0.015872 | 0.002512 | T | C | 0.0041 | 0.0225 |
| rs3184504 | C | T | 0.021388 | 0.002463 | C | T | -0.0208 | 0.0222 |
| rs34562254 | A | G | -0.03754 | 0.004139 | A | G | -0.0278 | 0.0365 |
| rs34931250 | T | C | 0.03341 | 0.005134 | T | C | -0.0781 | 0.0443 |
| rs35123414 | C | T | 0.021697 | 0.003622 | C | T | -0.0711 | 0.0285 |
| rs35676551 | A | C | -0.0379 | 0.00554 | A | C | 0.064 | 0.0447 |
| rs36090025 | C | A | 0.017388 | 0.002687 | C | A | -0.0137 | 0.0276 |
| rs3740688 | T | G | 0.024193 | 0.002475 | T | G | 0.0581 | 0.0247 |
| rs3768321 | T | G | -0.02842 | 0.003099 | T | G | 0.0078 | 0.0296 |
| rs378740 | A | C | 0.01565 | 0.002779 | A | C | -0.0402 | 0.0223 |
| rs3810484 | G | A | 0.014777 | 0.002477 | G | A | -0.0178 | 0.0225 |
| rs390801 | C | T | 0.021268 | 0.002802 | C | T | -0.0214 | 0.0245 |
| rs4327724 | T | C | 0.031296 | 0.004594 | T | C | -0.0065 | 0.0353 |
| rs4410790 | C | T | 0.015033 | 0.002557 | C | T | -0.006 | 0.0232 |
| rs4458838 | A | G | -0.01566 | 0.002581 | A | G | 0.0159 | 0.0232 |
| rs4499445 | T | G | -0.02174 | 0.002604 | T | G | -0.0196 | 0.0256 |
| rs45439091 | T | G | 0.031713 | 0.004747 | T | G | 0.0953 | 0.0664 |
| rs45512696 | T | C | 0.080056 | 0.003232 | T | C | 0 | 0.0305 |
| rs459193 | G | A | 0.022894 | 0.002978 | G | A | -0.0581 | 0.0234 |
| rs4711399 | T | C | 0.021968 | 0.002966 | T | C | -0.0356 | 0.0251 |
| rs473919 | G | C | -0.01888 | 0.003124 | G | C | 0.0274 | 0.027 |
| rs4782568 | G | C | -0.01843 | 0.002489 | G | C | -0.0325 | 0.0222 |
| rs4790875 | T | C | -0.02071 | 0.002513 | T | C | 0.0291 | 0.0223 |
| rs4796307 | A | T | -0.02815 | 0.003001 | A | T | -0.0015 | 0.0302 |
| rs4804413 | T | C | 0.017594 | 0.002488 | T | C | -0.0025 | 0.0223 |
| rs4805129 | C | T | -0.02111 | 0.002546 | C | T | 0.0161 | 0.0235 |
| rs4805881 | C | A | -0.02704 | 0.002609 | C | A | -0.0188 | 0.023 |
| rs4833945 | C | T | 0.021705 | 0.003854 | C | T | 0.0009 | 0.0321 |
| rs4946811 | C | A | 0.015897 | 0.002567 | C | A | -0.0386 | 0.0221 |
| rs4970834 | T | C | -0.02539 | 0.003169 | T | C | 0.0655 | 0.0275 |
| rs55696240 | A | G | 0.017035 | 0.002551 | A | G | -0.0324 | 0.0226 |
| rs55722786 | T | G | 0.028148 | 0.002735 | T | G | -0.0631 | 0.0263 |
| rs55724869 | C | G | 0.014174 | 0.002501 | C | G | 0.0229 | 0.0223 |
| rs55846720 | A | G | -0.0148 | 0.002479 | A | G | -0.0042 | 0.0222 |
| rs56094005 | G | A | -0.04654 | 0.006021 | G | A | 0.0345 | 0.0517 |
| rs56188865 | C | T | 0.016247 | 0.002556 | C | T | 0.0522 | 0.0229 |
| rs58558667 | T | G | 0.023041 | 0.002553 | T | G | -0.0732 | 0.0221 |
| rs58579887 | C | T | 0.018348 | 0.002522 | C | T | 0.0085 | 0.0224 |
| rs59431480 | G | C | 0.085838 | 0.012738 | G | C | 0.1665 | 0.1678 |
| rs59916403 | T | G | -0.0243 | 0.002576 | T | G | 0.0117 | 0.0221 |
| rs6031847 | T | C | -0.02127 | 0.002777 | T | C | 0.0428 | 0.0258 |
| rs60644673 | T | G | -0.01754 | 0.003122 | T | G | -0.0139 | 0.0263 |
| rs6065295 | T | C | 0.015019 | 0.002491 | T | C | -0.0223 | 0.0218 |
| rs61552236 | ACT | A | -0.0161 | 0.002776 | ACT | A | 0.0207 | 0.0247 |
| rs61817641 | T | C | -0.01821 | 0.002782 | T | C | 0.0517 | 0.0249 |
| rs61983272 | C | G | -0.03286 | 0.002844 | C | G | -0.0141 | 0.0238 |
| rs62053895 | A | G | -0.01385 | 0.002519 | A | G | -0.0121 | 0.0233 |
| rs62235071 | T | C | -0.04014 | 0.00522 | T | C | 0.0046 | 0.061 |
| rs631695 | G | T | 0.015489 | 0.002494 | G | T | -0.0134 | 0.0222 |
| rs6490409 | G | A | -0.01943 | 0.003511 | G | A | -0.0149 | 0.0262 |
| rs6549406 | G | A | -0.01898 | 0.002788 | G | A | -0.0366 | 0.023 |
| rs6567095 | A | G | 0.01941 | 0.002483 | A | G | -0.0098 | 0.0222 |
| rs667172 | A | G | 0.018923 | 0.002725 | A | G | -0.0034 | 0.0243 |
| rs6693993 | C | G | 0.023539 | 0.00247 | C | G | 0.0083 | 0.0219 |
| rs6734238 | G | A | -0.016 | 0.002507 | G | A | -0.0193 | 0.0238 |
| rs673751 | C | A | -0.01529 | 0.002638 | C | A | 0.0109 | 0.0229 |
| rs67694436 | T | C | 0.01512 | 0.002586 | T | C | -0.0537 | 0.0224 |
| rs6793835 | A | G | 0.016418 | 0.002803 | A | G | 0.0033 | 0.027 |
| rs6794370 | C | A | -0.01926 | 0.003127 | C | A | 0.0123 | 0.0255 |
| rs6860245 | C | G | -0.02143 | 0.002858 | C | G | -0.0295 | 0.0301 |
| rs6871748 | C | T | 0.015224 | 0.002757 | C | T | -0.0072 | 0.0232 |
| rs6897617 | A | G | 0.018249 | 0.002716 | A | G | -0.0422 | 0.0223 |
| rs6912315 | T | C | 0.031344 | 0.005494 | T | C | -0.012 | 0.0836 |
| rs6970593 | A | G | -0.0255 | 0.002472 | A | G | 0.0037 | 0.0222 |
| rs7031621 | A | G | -0.01438 | 0.002461 | A | G | -0.0288 | 0.0221 |
| rs72631343 | G | C | 0.021597 | 0.003685 | G | C | 0.038 | 0.0313 |
| rs72694393 | T | G | 0.018287 | 0.002479 | T | G | -0.0187 | 0.0225 |
| rs72789541 | A | T | 0.018236 | 0.002703 | A | T | -0.0074 | 0.0235 |
| rs72793380 | A | C | 0.02248 | 0.004032 | A | C | 0.0138 | 0.0384 |
| rs72818989 | C | T | 0.015766 | 0.002484 | C | T | 0.0292 | 0.022 |
| rs72959041 | A | G | 0.051281 | 0.005716 | A | G | -0.008 | 0.0451 |
| rs73038384 | T | C | -0.05206 | 0.007016 | T | C | 0.0511 | 0.0548 |
| rs73234873 | T | C | -0.01499 | 0.00271 | T | C | 0.0189 | 0.0265 |
| rs73263719 | A | G | 0.019352 | 0.00348 | A | G | -0.0107 | 0.0366 |
| rs7366884 | C | T | 0.018621 | 0.002762 | C | T | 0.0037 | 0.0256 |
| rs7402977 | A | G | -0.01627 | 0.002803 | A | G | -0.0041 | 0.0244 |
| rs74502455 | G | C | -0.02087 | 0.002464 | G | C | -0.0154 | 0.0219 |
| rs74538877 | C | G | 0.031123 | 0.005498 | C | G | 0.0016 | 0.0457 |
| rs74780677 | G | A | 0.093012 | 0.010096 | G | A | -0.1147 | 0.0424 |
| rs75070309 | A | C | 0.03197 | 0.003228 | A | C | 0.0098 | 0.0321 |
| rs7591567 | C | T | 0.015616 | 0.002704 | C | T | -0.0017 | 0.0235 |
| rs76895963 | G | T | -0.07122 | 0.009449 | G | T | 0.0923 | 0.0652 |
| rs7731045 | C | T | -0.01422 | 0.002543 | C | T | 0.0181 | 0.0223 |
| rs77542162 | G | A | -0.19345 | 0.008216 | G | A | 0.0735 | 0.1345 |
| rs77849807 | G | A | 0.11375 | 0.00989 | G | A | 0.0074 | 0.0703 |
| rs78444263 | T | C | -0.03521 | 0.004796 | T | C | 0.0109 | 0.0354 |
| rs78961851 | C | T | -0.0248 | 0.004077 | C | T | -0.0221 | 0.0415 |
| rs79295634 | G | A | 0.03133 | 0.004938 | G | A | -0.073 | 0.0404 |
| rs800545 | G | A | -0.01617 | 0.002833 | G | A | 0.0024 | 0.0246 |
| rs8041057 | T | C | -0.02322 | 0.002722 | T | C | 0.0049 | 0.0235 |
| rs8072215 | G | A | -0.03389 | 0.002914 | G | A | 0.0143 | 0.0255 |
| rs854796 | A | G | 0.015472 | 0.002654 | A | G | 0.0057 | 0.0239 |
| rs872926 | A | G | -0.03583 | 0.003037 | A | G | 0.0131 | 0.0287 |
| rs879620 | T | C | -0.01419 | 0.002541 | T | C | 0.0028 | 0.0222 |
| rs900400 | C | T | 0.022534 | 0.002516 | C | T | -0.0692 | 0.0235 |
| rs9265945 | G | A | -0.02345 | 0.0025 | G | A | -0.0329 | 0.0233 |
| rs930340 | A | G | -0.01888 | 0.00316 | A | G | -0.0066 | 0.0292 |
| rs9391997 | G | A | -0.01621 | 0.002476 | G | A | 0.0099 | 0.0218 |
| rs9638180 | G | A | -0.02447 | 0.003091 | G | A | 0.0458 | 0.0285 |
| rs9917677 | C | T | -0.01551 | 0.00283 | C | T | -0.0083 | 0.0263 |
| rs992367 | G | A | 0.015386 | 0.002521 | G | A | -0.0133 | 0.0233 |
| rs9976946 | T | C | 0.038971 | 0.006444 | T | C | -0.0612 | 0.0846 |

SNP, single nucleotide polymorphism; PHD, pulmonary heart disease; EA, effect allele; OA, other allele; SE, standard error

**Supplementary Table S9.** MR results and pleiotropy test

| **Outcomes** | **IVW** | | | **MR egger** | | | **weighted median** | | | **intercept of pleiotropy** | **p for pleiotropy** | **Cochrane's Q value** |
| --- | --- | --- | --- | --- | --- | --- | --- | --- | --- | --- | --- | --- |
|  | **OR** | **95% CI** | **p** | **OR** | **95% CI** | **p** | **OR** | **95% CI** | **p** |  |  |  |
| VTE | 0.993 | 0.991, 0.995 | 4.32E-12 | 0.993 | 0.989, 0.997 | 0.0005 | 0.994 | 0.991, 0.997 | 0.0001 | 7.80E-06 | 0.88 | 253.98 |
| Stroke | 0.997 | 0.995, 0.999 | 0.002 | 0.999 | 0.995, 1.002 | 0.4619 | 0.997 | 0.994, 1.000 | 0.0745 | -4.85E-05 | 0.33 | 190.75 |
| AF | 0.922 | 0.870, 0.977 | 0.006 | 0.927 | 0.829, 1.037 | 0.1876 | 0.917 | 0.847, 0.993 | 0.0322 | -0.000177548 | 0.91 | 300.61 |
| HF | 1.001 | 1.000, 1.002 | 0.293 | 1.001 | 0.999, 1.002 | 0.5718 | 1.000 | 0.998, 1.002 | 0.9512 | -5.05E-07 | 0.98 | 230.54 |
| CAD | 1.001 | 0.917, 1.093 | 0.976 | 0.878 | 0.738, 1.045 | 0.1446 | 0.950 | 0.849, 1.062 | 0.3650 | 0.003934505 | 0.09 | 305.04 |
| T2DM | 1.045 | 0.940, 1.093 | 0.416 | 0.962 | 0.702, 1.319 | 0.8098 | 0.996 | 0.866, 1.145 | 0.9520 | 0.001884258 | 0.59 | 233.58 |
| PHD | 0.737 | 0.622, 0.874 | 0.0004343 | 0.701 | 0.489, 1.010 | 0.0579 | 0.8011 | 0.629, 1.043 | 0.1017 | 0.001360955 | 0.77 | 234.48 |

OR, Odds ratio; CI, Confidence interval; IVW, inverse-variance weighted; VTE, Venous Throm-boembolism.AF, atrial fibrillation; HF, heart failure; CAD, coronary artery disease; T2DM, type 2 diabetes; PHD, pulmonary heart disease.

**Supplementary Figure S1**


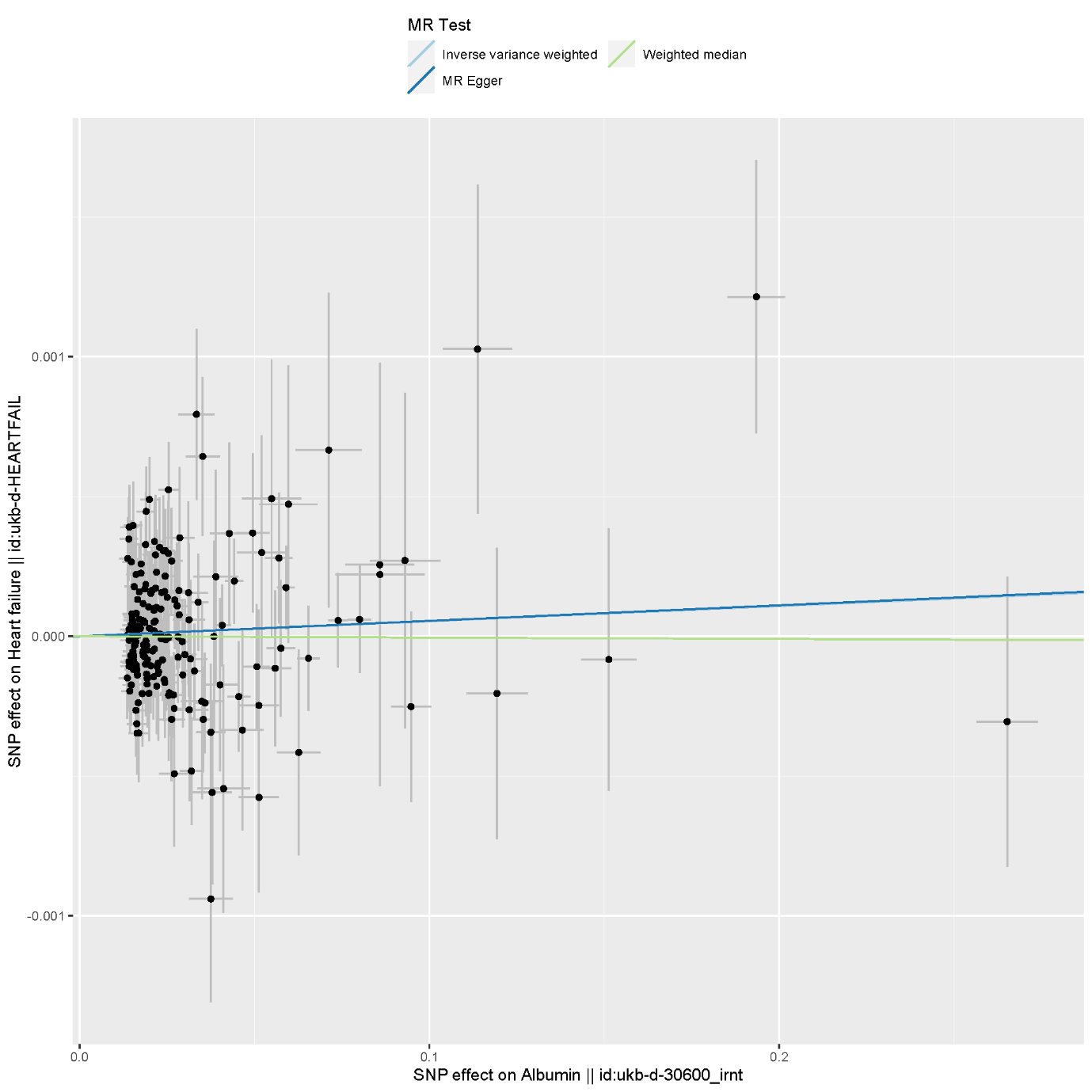


**Supplementary Figure S2**


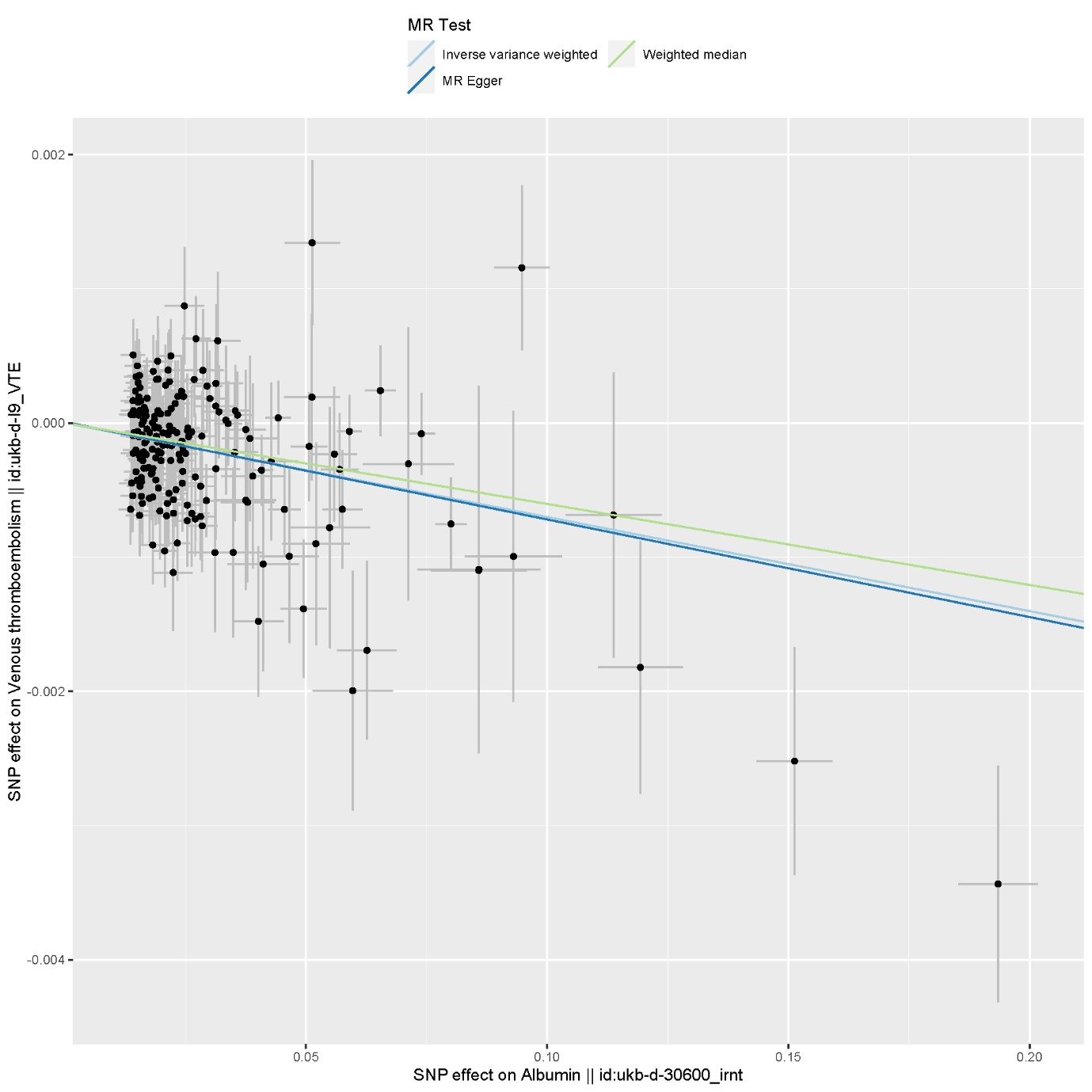


**Supplementary Figure S3**


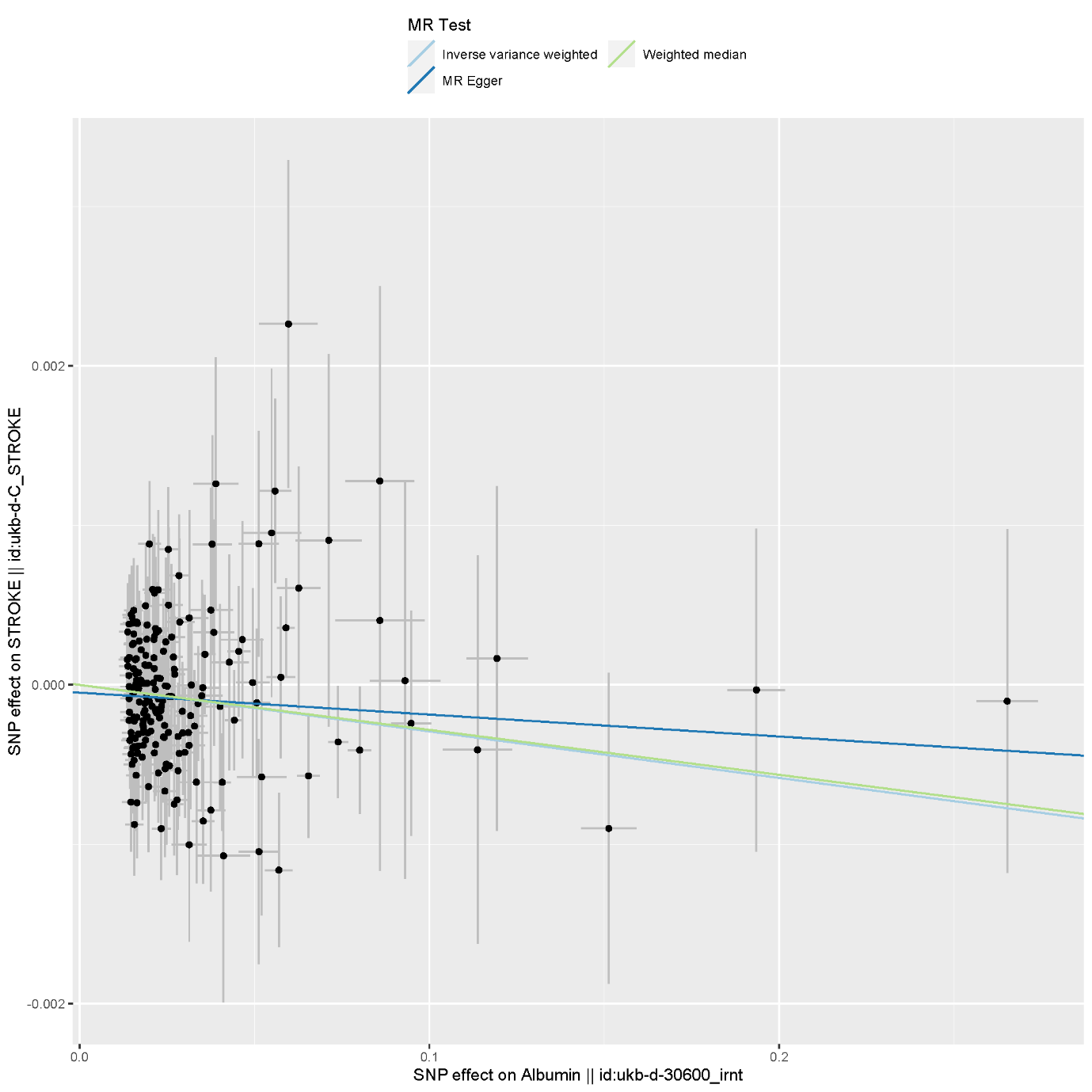


**Supplementary Figure S4**


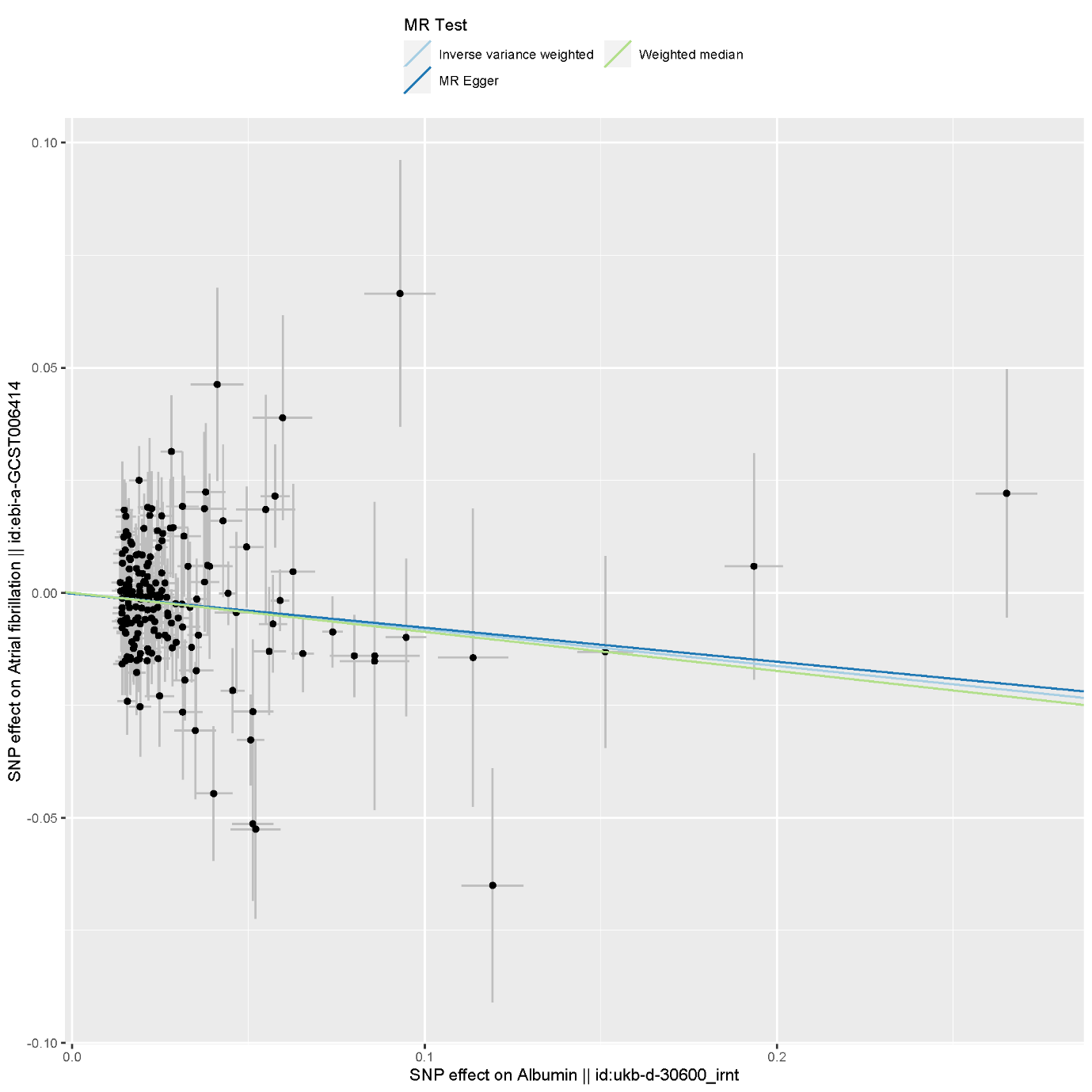


**Supplementary Figure S5**


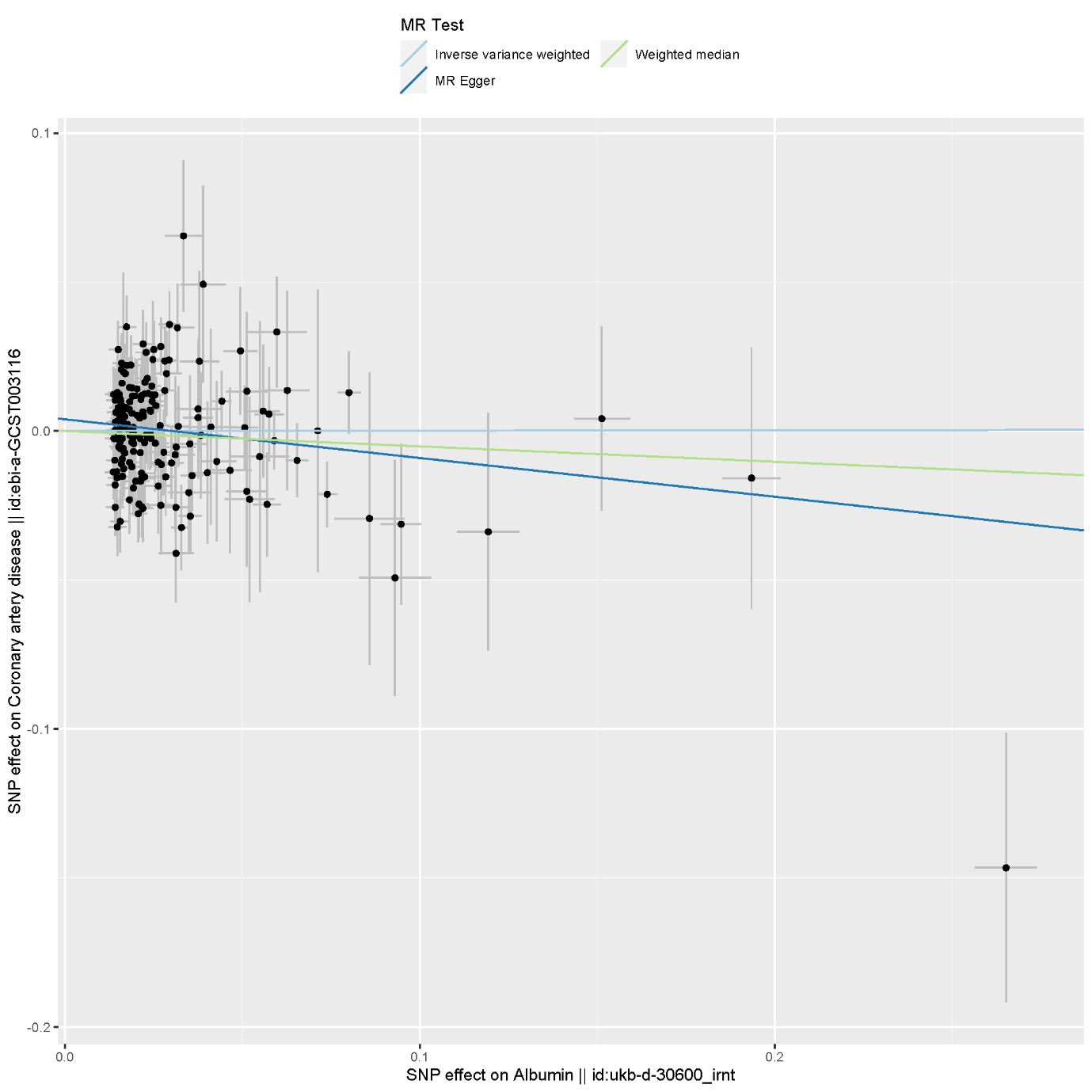


**Supplementary Figure S6**


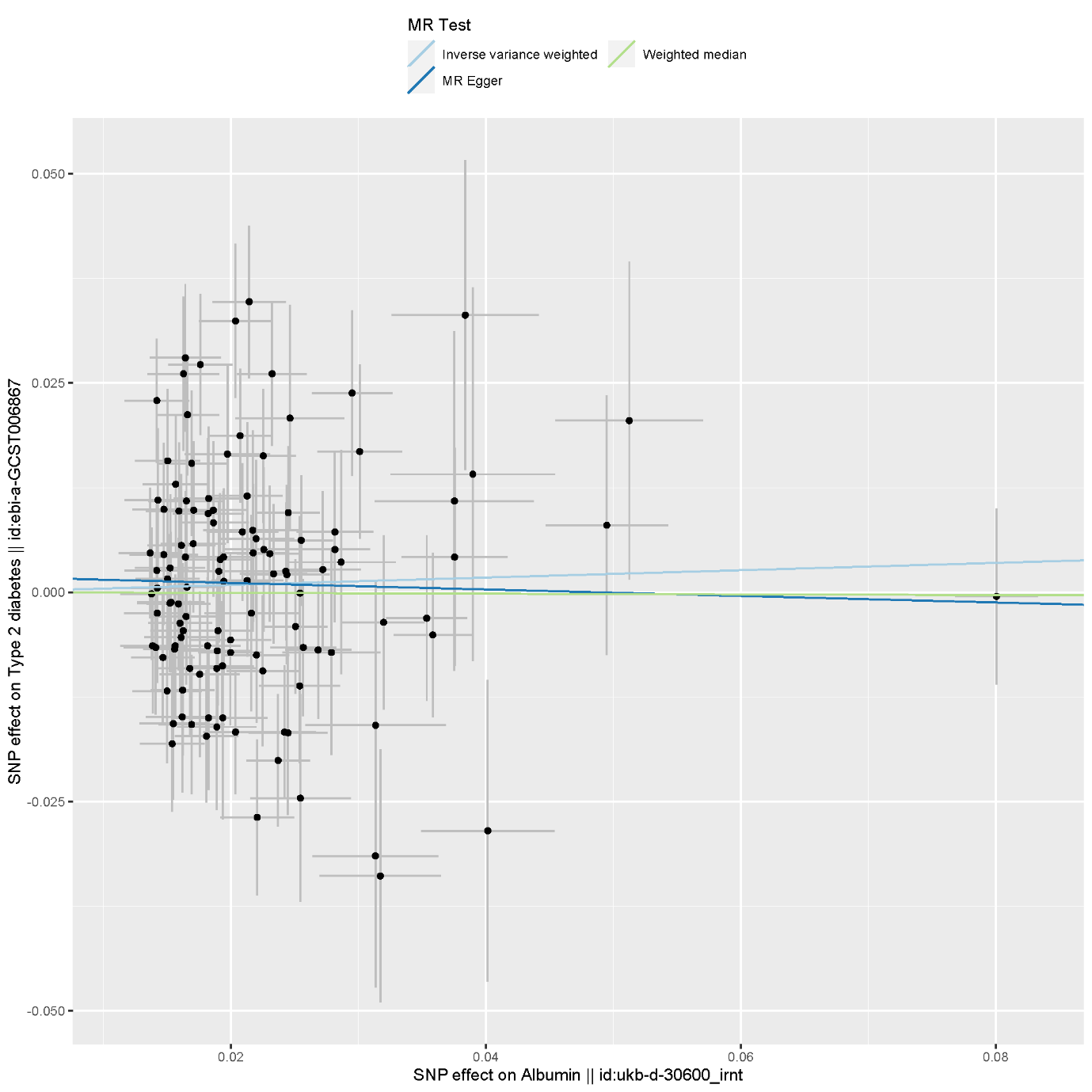


**Supplementary Figure S7**


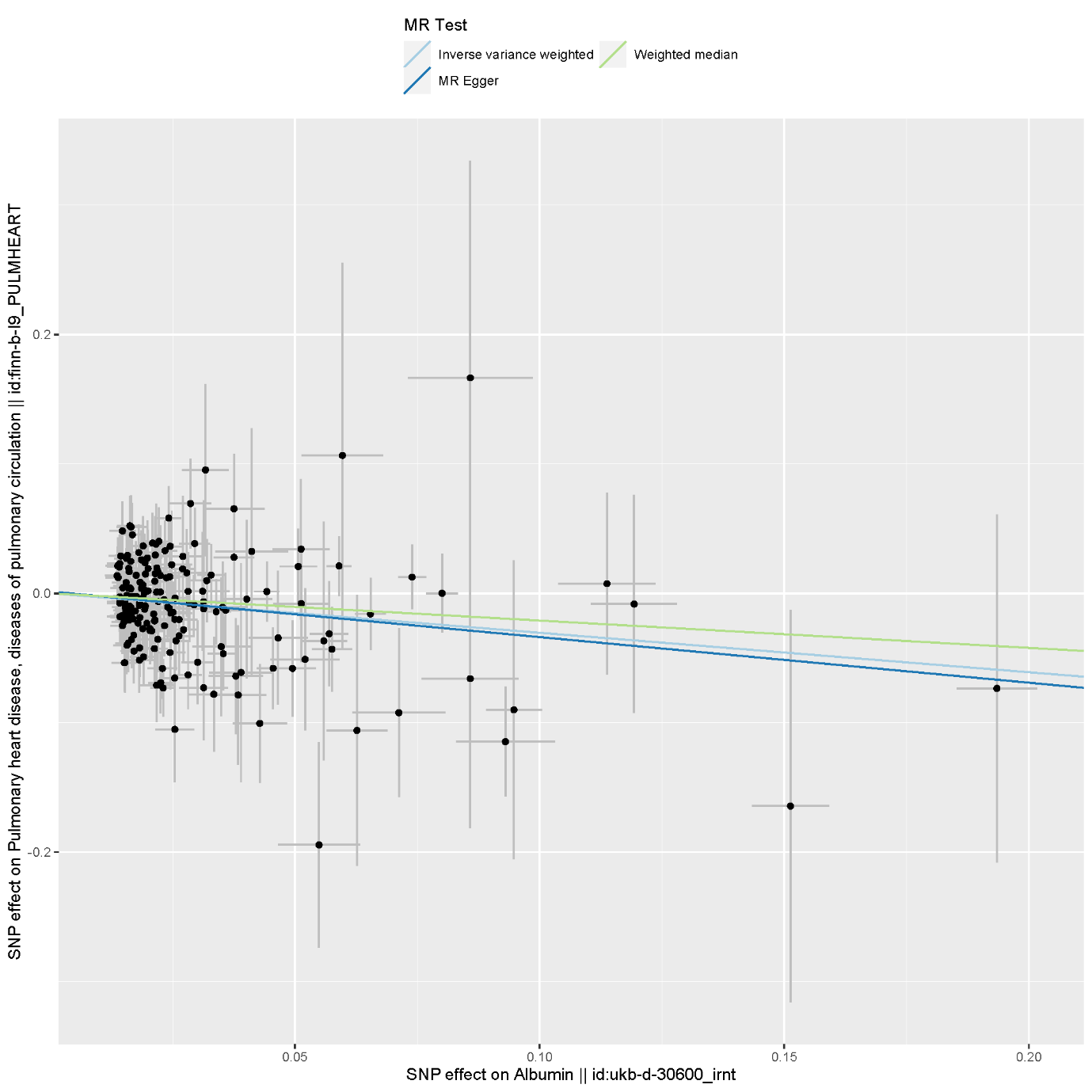


**Supplementary Figure S8**


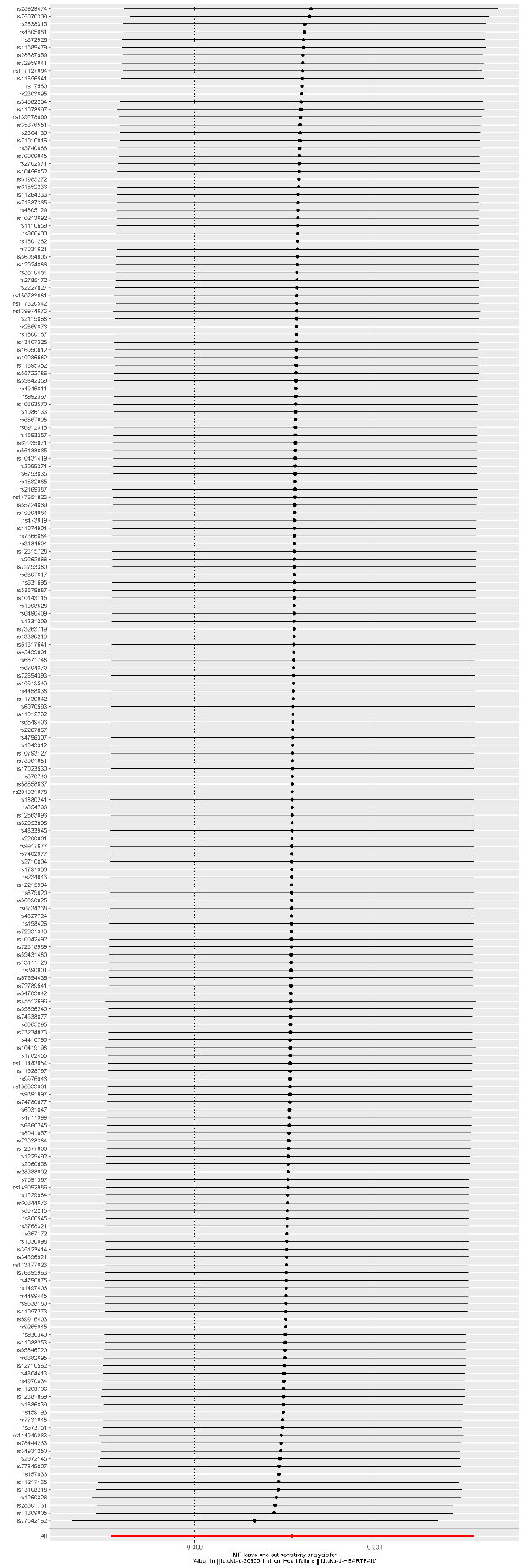


**Supplementary Figure S9**


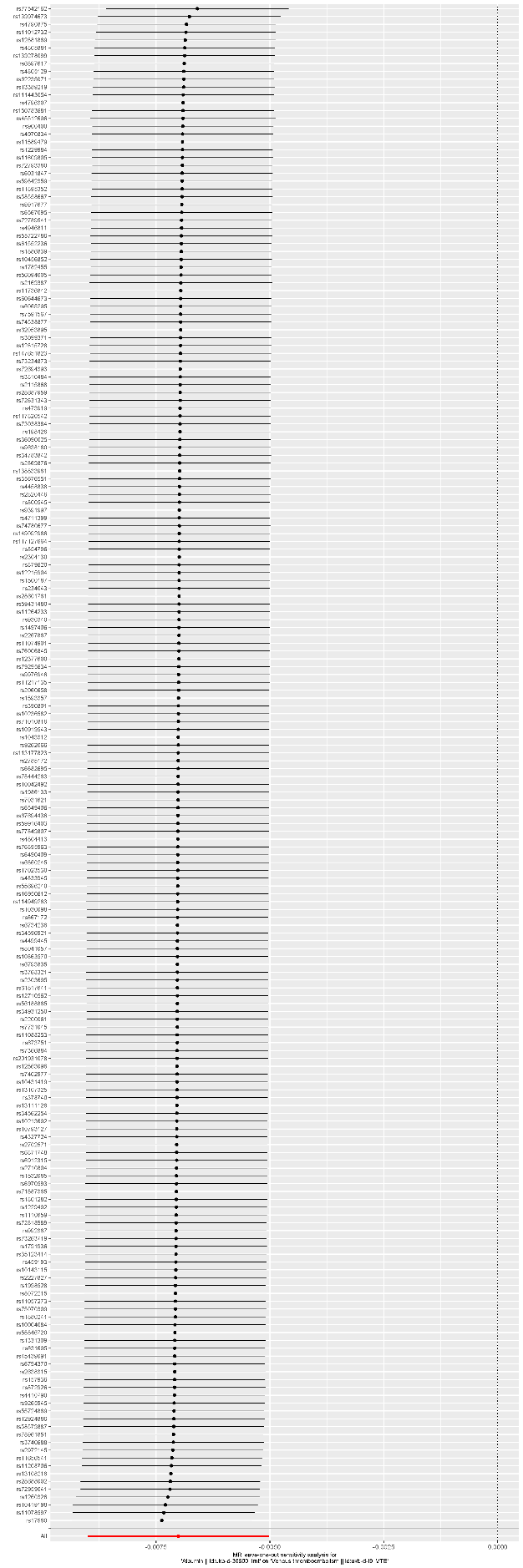


**Supplementary Figure S10**


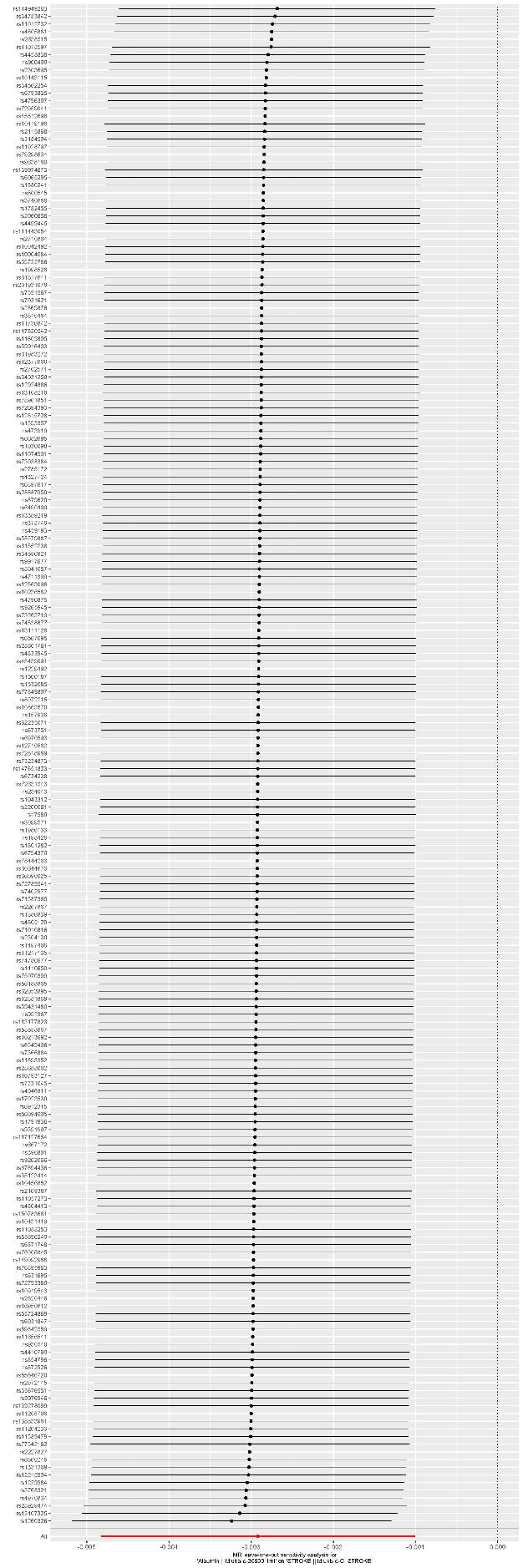


**Supplementary Figure S11**


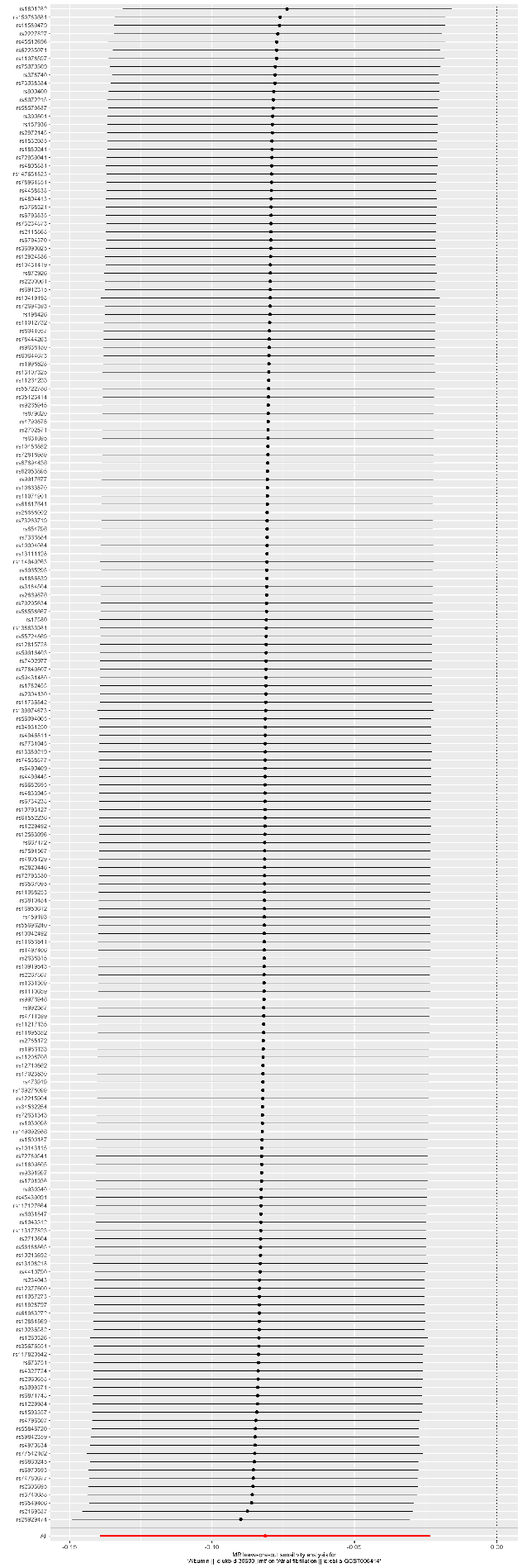


**Supplementary Figure S12**


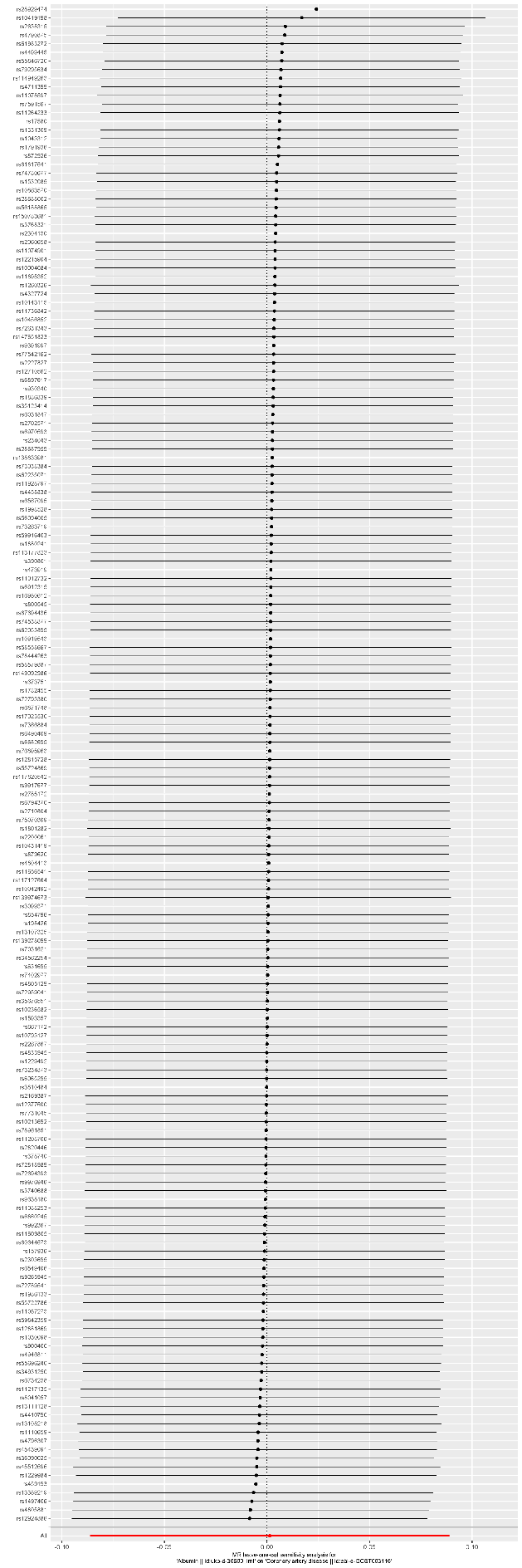


**Supplementary Figure S13**


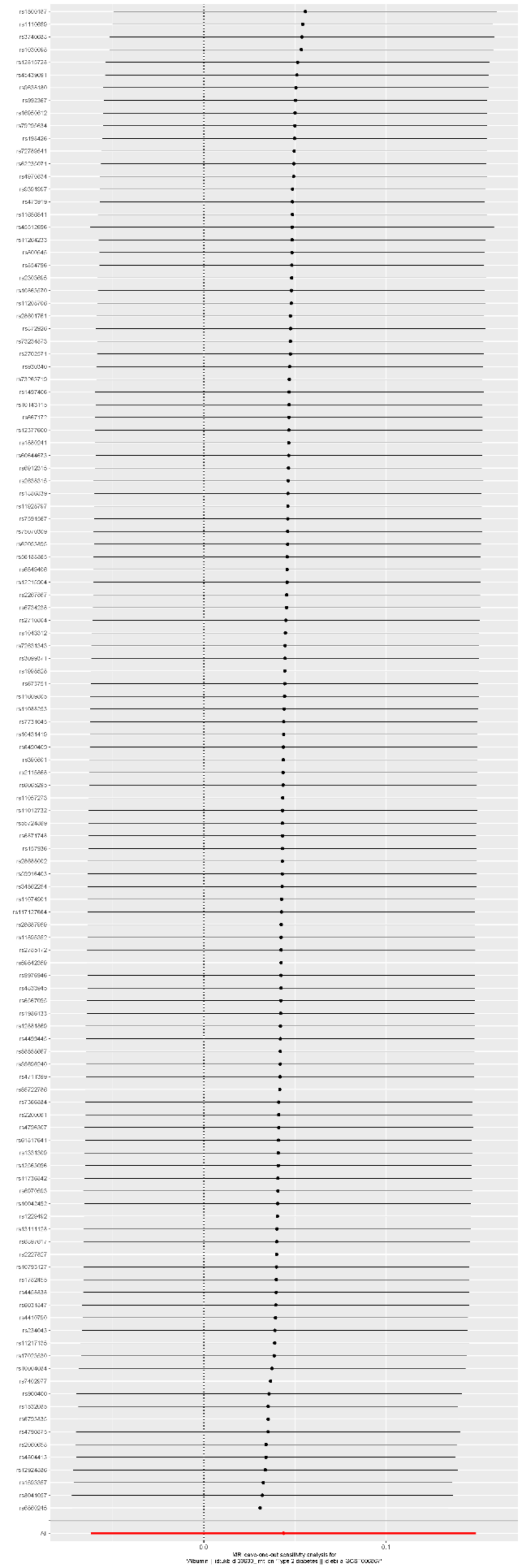


**Supplementary Figure S14**


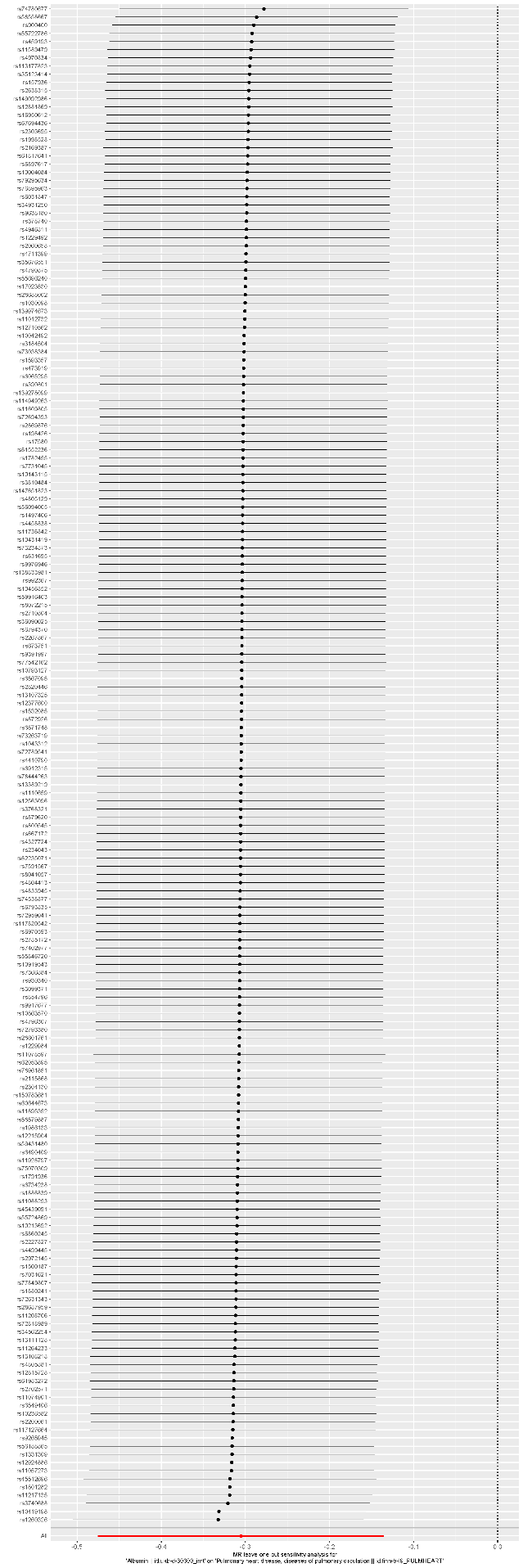

Supplement: Supplementary file 1 — Supplementary Material 1. [file 12872_2024_3873_MOESM1_ESM.docx]
